# A randomized study to evaluate safety and immunogenicity of the BNT162b2 COVID-19 vaccine in healthy Japanese adults

**DOI:** 10.1038/s41467-021-27316-2

**Published:** 2021-12-14

**Authors:** Miwa Haranaka, James Baber, Yoichiro Ogama, Masako Yamaji, Masakazu Aizawa, Osamu Kogawara, Ingrid Scully, Eleni Lagkadinou, Ӧzlem Türeci, Uğur Şahin, Philip R. Dormitzer, William C. Gruber, Stephen Lockhart

**Affiliations:** 1 SOUSEIKAI PS Clinic, Fukuoka, Japan; 2 Vaccine Clinical Research, Pfizer Inc, Sydney, NSW Australia; 3 SOUSEIKAI Sumida Hospital, Tokyo, Japan; 4 Pfizer R&D Japan G.K., Tokyo, Japan; 5 Vaccine Research and Development, Pfizer Inc, Pearl River, NY USA; 6 BioNTech, Mainz, Germany; 7 Vaccine Research and Development, Pfizer Inc, Hurley, UK

**Keywords:** Phase I trials, RNA vaccines, SARS-CoV-2

## Abstract

We report interim safety and immunogenicity findings from an ongoing phase 1/2 study of BNT162b2 in healthy Japanese adults. Participants were randomized 3:1 to receive 2 intramuscular injections of 30 μg BNT162b2 or placebo 21 days apart. Overall, 160 individuals were randomized: 119 received BNT162b2, and 41 received placebo. Participants were stratified by age: 20–64 years (*n* = 130) and 65–85 years (*n* = 30). More than 97% of BNT162b2 recipients received 2 doses. Local reactions and systemic events were generally transient and mild to moderate. Severe adverse events were uncommon; there were no serious adverse events. One month after dose 2, SARS-CoV-2 50% serum neutralizing geometric mean titers were 571 and 366, and geometric mean fold rises were 55.8 and 36.6, in the younger and older age groups, respectively. In summary, BNT162b2 has an acceptable safety profile and produces a robust immune response, regardless of age, in Japanese adults. (ClinicalTrials.gov, NCT04588480).

## Abstract (web summary)

Here the authors provide the interim analysis of an ongoing phase 1/2 study of the BNT162b2 vaccine in healthy Japanese adults. They report mainly mild to moderate local reactions and no serious adverse events as well as good antibody induction one month after the second dose.

## Introduction

Since the start of the coronavirus disease 2019 (COVID-19) pandemic, more than 247 million people have been infected globally, and more than 5 million have died^1^. In Japan, as of November 4, 2021, more than 1.7 million cases have been reported, with more than 18,000 deaths^1^. BNT162b2 and other vaccines against severe acute respiratory syndrome coronavirus 2 (SARS-CoV-2) have been authorized for emergency use around the world, including special approval in Japan^1–4^. BNT162b2 is fully licensed for immunization in the United States, from 16 years of age^5^, and authorized in Japan in individuals ≥12 years of age^6^.

BNT162b2 is a lipid nanoparticle formulation that contains nucleoside-modified messenger RNA that encodes the conformationally stabilized full-length SARS-CoV-2 viral spike glycoprotein^7,8^. Trials in healthy adults show that 2 doses of 30 µg BNT162b2 21 days apart elicit high neutralizing antibody titers and robust, antigen-specific Th1 CD4+ and interferon-γ+ CD8+ T-cell anti‒SARS-CoV-2 responses^9,10^. Data have been previously reported from phase 2/3 of a pivotal global phase 1/2/3 randomized controlled trial (conducted in the United States, Argentina, Brazil, South Africa, Germany, and Turkey) to evaluate the safety, immunogenicity, and efficacy of 2 doses of 30 µg BNT162b2 in preventing COVID-19 in participants ≥12 years of age^11,12^. The safety profile in the pivotal trial was favorable, with generally short-term mild to moderate local reactogenicity, including fatigue and headache^11,12^. Vaccine efficacy from 7 days after the second dose was 95% in participants ≥16 years old, including the subgroup of participants ≥65 years old^11^. In adolescents 12–15 years of age, observed vaccine efficacy was 100% from 7 days after the second dose^12^. Longer-term data show that up to 6 months after dose 2, vaccine efficacy remains high (>90%), including in participants ≥65 years old^13^.

Here we report interim safety and immunogenicity findings through 1 month after dose 2 from an ongoing phase 1/2 study (NCT04588480) of BNT162b2 in Japanese adults 20–85 years of age.

## Results

### Participants

**Fig. 1:**
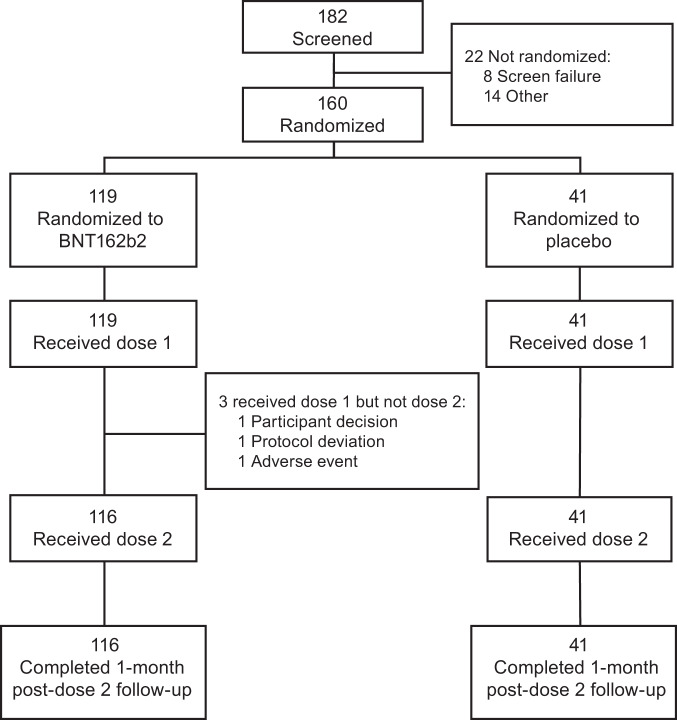
Study flow diagram. Includes all screened and randomized participants. Participants who received dose 1 but not dose 2 continued to be evaluated for safety and immunogenicity.

Between October 21, 2020, and November 10, 2020, 160 individuals were randomized at 2 sites (1 hospital and 1 clinic) in Japan; 119 received BNT162b2 and 41 received placebo (Fig. 1). More than 97% of BNT162b2 recipients received 2 doses. All participants were Japanese, 51% were male, and the mean age was 46 years (range 20–76 years); 130 participants were in the younger age group (20–64 years of age), and 30 participants were in the older age group (65–85 years of age) (Table 1). The most commonly reported comorbidities across all participants were dyslipidemia (4/119 [3.4%] BNT162b2 recipients; 2/41 [4.9%] placebo recipients) and hypertension (2/119 [1.7%] BNT162b2 recipients; 2/41 [4.9%] placebo recipients).

**Table 1:** Demographic characteristics of participants.

Characteristic	BNT162b2		Placebo		Overall
	20–64 years of age (*N* = 97)	65–85 years of age (*N* = 22)	20–64 years of age (*N* = 33)	65–85 years of age (*N* = 8)	(*N* = 160)
Male, *n* (%)	50 (51.5)	9 (40.9)	16 (48.5)	6 (75.0)	81 (50.6)
Race, *n* (%)					
Asian	97 (100.0)	22 (100.0)	33 (100.0)	8 (100.0)	160 (100.0)
Ethnicity, *n* (%)					
Non-Hispanic/non-Latinx	97 (100.0)	22 (100.0)	33 (100.0)	8 (100.0)	160 (100.0)
Racial designation, *n* (%)					
Japanese	97 (100.0)	22 (100.0)	33 (100.0)	8 (100.0)	160 (100.0)
Age at vaccination, years					
Mean (SD)	41.5 (12.83)	70.2 (3.26)	38.3 (13.20)	71.3 (3.20)	46.3 (16.55)
Median (range)	43.0 (20–63)	71.5 (65–74)	37.0 (20–60)	70.5 (67–76)	47.0 (20–76)

Results are for the safety population.

*SD* standard deviation.

### Safety

The safety population included all participants who received ≥1 dose of study intervention.

### Reactogenicity

**Fig. 2:**
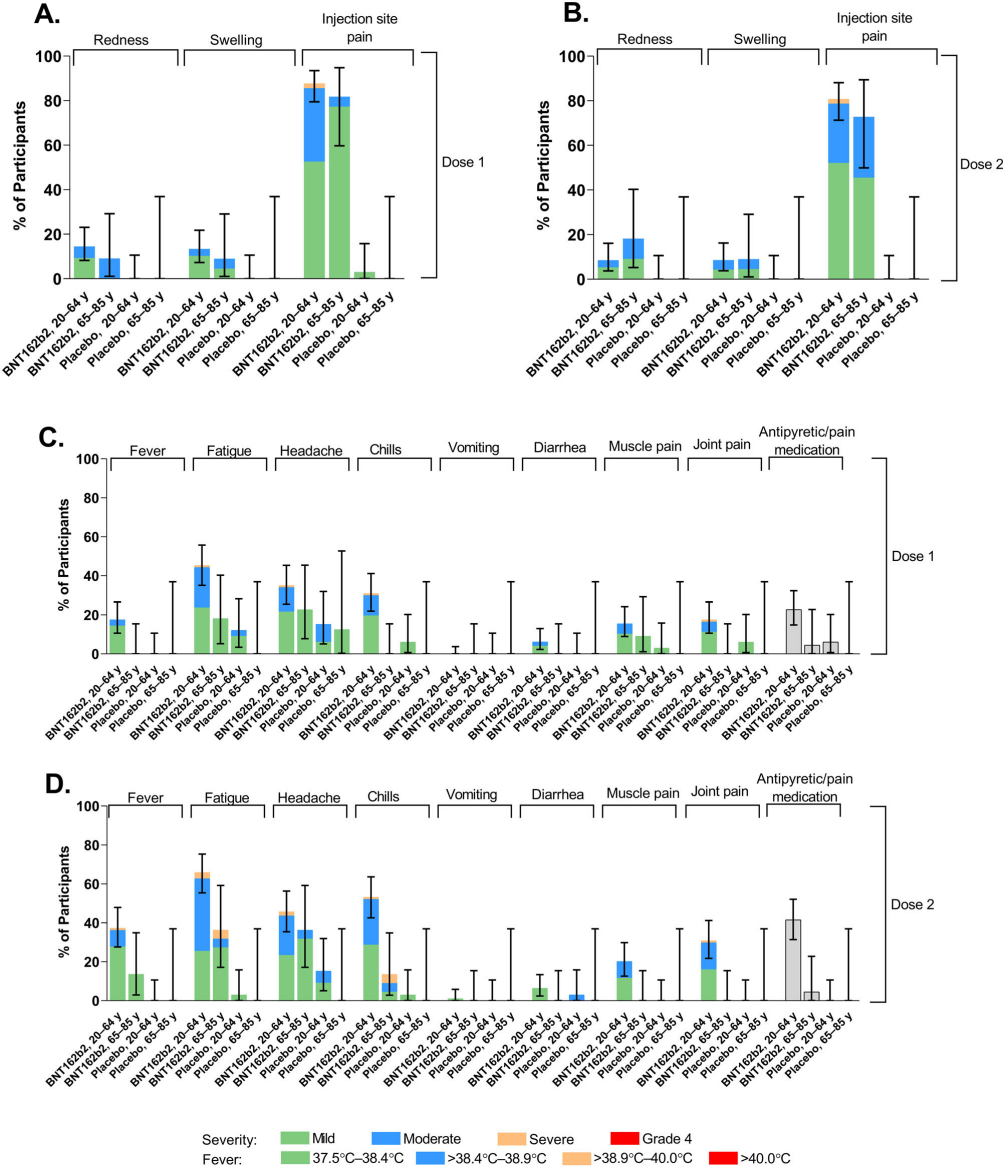
Local reactions and systemic events reported within 7 days after administration of BNT162b2 or placebo in participants 20–64 years of age and 65–85 years of age. **A**, **B** Local reactions after doses 1 and 2, respectively. **C**, **D** Systemic events after doses 1 and 2, respectively. Results are for the safety population (20–64 years of age: *n* = 97 for BNT162b2, and *n* = 33 for placebo; 65–85 years of age: *n* = 22 for BNT162b2, and *n* = 8 for placebo). Pain at injection site was graded as mild (does not interfere with activity), moderate (interferes with activity), severe (prevents daily activity), or grade 4 (led to emergency department visit or hospitalization). Redness and swelling were graded as mild (>2.0–5.0 cm in diameter), moderate (>5.0–10.0 cm in diameter), severe (>10.0 cm in diameter), or grade 4 (necrosis or exfoliative dermatitis for redness and necrosis for swelling). Fever categories are shown in the key. Fatigue, headache, chills, and new or worsened muscle or joint pain were graded as mild (does not interfere with activity), moderate (some interference with activity), or severe (prevents daily activity). Vomiting was graded as mild (1–2 times in 24 h), moderate (>2 times in 24 h), or severe (requires intravenous hydration), and diarrhea as mild (2–3 loose stools in 24 h), moderate (4–5 loose stools in 24 h) or severe (≥6 loose stools in 24 h). Grade 4 for all systemic events indicated an emergency department visit or hospitalization. No participant experienced a grade 4 local reaction or systemic event. Data are presented as percentages with associated 95% CIs shown as error bars for the percentage of participants experiencing any reaction.

Injection site pain was the most commonly reported local reaction after each dose of BNT162b2 regardless of age group (Fig. 2A, B). Most local reactions were mild to moderate and generally transient (median duration, 1–3.5 days); 4 BNT162b2 recipients reported severe injection site pain. Mild injection site pain was the only local reaction reported in the placebo group (by 1 participant). Fatigue, headache, and chills were the most frequently reported systemic events; these were generally transient (median duration, 1–2 days), mild to moderate in severity, and more commonly reported in the younger age group (Fig. 2C, D). Severe systemic events after dose 1 of BNT162b2 occurred in 1 participant (1.0%); this participant experienced severe headache, chills, fatigue, and new or worsened joint pain, all of which were also reported as AEs. After dose 2 of BNT162b2, severe fatigue was reported by 4 participants (3.4%, 1 in the older age group and 3 in the younger age group); severe headache was reported by 2 participants (1.7%, both in the younger group); severe chills were reported by 2 participants (1.7%, 1 in each age group); and severe new or worsened joint pain was reported by 1 participant (0.9%, in the younger age group). There were no reports of fever >40 °C, and only 1 participant reported fever >38.9 °C. Analgesic or antipyretic medications were more commonly taken by BNT162b2 recipients than by placebo recipients after each dose (Fig. 2C, D).

### Adverse events

Adverse events (AEs) from dose 1 through 1 month after dose 2 were reported by 10.1% of BNT162b2 and 7.3% of placebo recipients (Table 2). AEs reported by more than 1 participant in either group were nasopharyngitis (BNT162b2, *n* = 3 [2.5 %]; placebo, *n* = 1 [2.4 %]) and headache (BNT162b2, *n* = 2 [1.7 %]; placebo, *n* = 1 [2.4 %]). There were no immediate AEs within 30 min of vaccination. There were no serious AEs, no life-threatening AEs, and no deaths through 1 month after dose 2. There was no reported lymphadenopathy, and there were no diagnoses of COVID-19.

**Table 2:** Participants reporting at least 1 adverse event from dose 1 through 1 month after dose 2.

Adverse event	BNT162b2 (*N*^a^ = 119) *n*^b^ (%)	Placebo (*N*^a^ = 41) *n*^b^ (%)
Any event	12 (10.1)	3 (7.3)
Related^c^	2 (1.7)	0
Severe^d^	1 (0.8)	0
Life threatening	0	0
Any serious adverse event	0	0
Related^c^	0	0
Severe^d^	0	0
Life threatening	0	0
Any adverse event leading to discontinuation	1 (0.8)	0
Related^c,d^	1 (0.8)	0
Severe^d^	1 (0.8)	0
Life threatening	0	0
Death	0	0

Results are for the safety population.

^a^Number of participants in the specified group. This value is the denominator for the percentage calculations.

^b^Number of participants reporting ≥1 occurrence of the specified event category. For “any event”, *n* = the number of participants reporting ≥1 occurrence of any event.

^c^Assessed by the investigator as related to investigational product; injection site pain, headache, chills, fatigue, and new or worsened joint pain in 1 participant, and erythema multiforme in 1 participant.

^d^Injection site pain, headache, chills, fatigue, and new or worsened joint pain in 1 participant.

As reported in the *Reactogenicity* section, 1 participant in the younger age group, a 25-year-old woman with no significant medical history, reported severe vaccine-related AEs, all of which started 1 day after dose 1 of BNT162b2. The AEs resolved as follows: chills after 1 day, headache and joint pain after 2 days, fatigue after 3 days, and injection site pain after 6 days. The participant discontinued the study intervention and did not receive dose 2 of BNT162b2. She continued to be followed up for safety and immunogenicity assessments.

One other AE considered vaccine related by the investigator was reported after BNT162b2 immunization. Moderate erythema multiforme occurred in a 74-year-old woman who had no relevant medical or allergic history, took no regular medications, and was not taking any medications at the time of AE onset. On the day following dose 1, she experienced mild swelling and pain at the injection site, fatigue, and headache. Two days after dose 2, she reported onset of skin rash and was treated with topical steroids, topical antihistamines, and oral antihistamines; the event resolved in 27 days. Skin biopsy confirmed the diagnosis of erythema multiforme.

### Clinical laboratory tests

The clinical laboratory subset included the first 24 participants: 12 participants in the younger age group (20–64 years of age) and 12 in the older age group (65–85 years of age). Laboratory abnormalities were uncommon, and almost all were mild. No laboratory abnormalities were reported as AEs. Transient decreases in lymphocyte counts in some BNT162b2 recipients after dose 1 resolved within 1 week.

### Immunogenicity

**Fig. 3:**
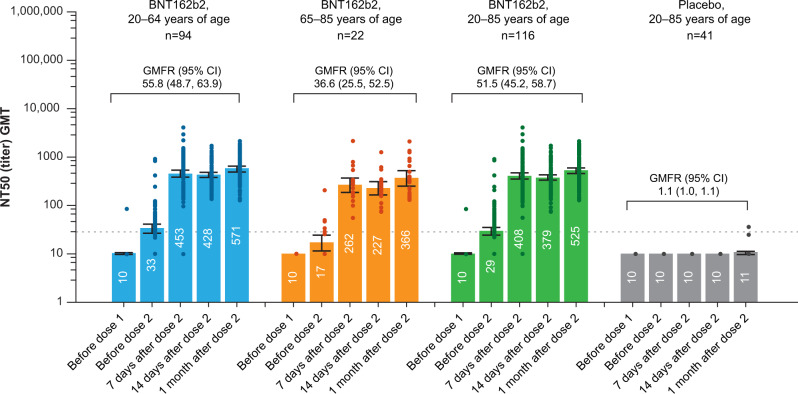
Geometric mean titers and geometric mean fold rises of SARS-CoV-2 50% neutralizing titers for participants by age group and overall. Results are for the evaluable immunogenicity population. Data are presented as geometric mean values with associated 95% CIs shown as error bars. Dots represent individual 50% neutralizing titers; individual dots correspond with discrete visits for each time point and may be superimposed. Numbers within bars are the geometric means. *n* = Number of participants with valid and determinate assay results. GMTs, GMFRs, and two-sided 95% CIs were calculated by exponentiation of the mean logarithm of the titers or fold rises and the corresponding CIs (based on the Student *t* distribution). GMFRs are from before dose 1 to 1 month after dose 2. Assay results below the LLOQ (20, dashed line) were set to 0.5 × LLOQ. CI confidence interval, GMFR geometric mean fold rise, GMT geometric mean titer, NT50 50% neutralizing titer, LLOQ lower limit of quantitation, SARS-CoV-2 severe acute respiratory syndrome coronavirus 2. In the placebo group, *n* = 41 except before dose 2, when *n* = 40.

Immunogenicity was assessed in the evaluable immunogenicity population, which includes all randomized participants who received 2 doses of BNT162b2 or placebo with ≥1 valid and determinate immunogenicity result after dose 2, blood collection within a predefined window after dose 2, and no other major protocol violations as determined by the investigator. Regardless of age, there was a robust immune response after immunization with BNT162b2: serum SARS-CoV-2 50% neutralizing geometric mean titers (GMTs) substantially increased by 7 days after dose 2, and remained elevated at all time points up to 1 month after dose 2 (Fig. 3). GMTs and associated geometric mean fold rises (GMFRs) were slightly lower in the older age group (65–85 years of age) compared with the younger age group (20–64 years of age). One month after dose 2 of BNT162b2, SARS-CoV-2 50% neutralization GMTs were 571 and 366, and GMFRs were 55.8 and 36.6 in the younger and older age groups, respectively.

## Discussion

The safety and efficacy of BNT162b2 has been established in a global pivotal trial and previously reported^11^. Subgroup analyses in the pivotal trial reported that vaccine efficacy was consistent across racial and ethnic subgroups^11^. Approximately 4% of participants in the pivotal trial were Asian race^11^. In compliance with Japanese regulatory requirements, we determined safety and immunogenicity in a Japanese population. We have shown here that 2 doses of 30 µg BNT162b2 administered 21 days apart were safe and immunogenic in healthy Japanese adults 20–85 years of age. As observed in earlier analyses^11,12^, adherence to the vaccine regimen was high, with >97% of BNT162b2 recipients receiving dose 2.

The favorable safety and tolerability profile of BNT162b2 previously observed in clinical trial participants ≥5 years old^11,12,14^ and from ongoing pharmacovigilance^15^ was also observed in healthy Japanese adults. Severe or vaccine-related AEs were uncommon. Transient decreases in blood lymphocyte counts observed in this study have been previously reported with BNT162b2^10^. Such decreases may be attributable to innate immune stimulation-related lymphocyte redistribution into lymphoid tissues^16^.

A 2-dose regimen of 30 μg BNT162b2, administered 21 days apart, elicited robust serum SARS-CoV-2 neutralization titers in younger and older Japanese adults by 7 days after dose 2, with persistence of titers to 1 month after dose 2, when the last assayed sera were drawn. The 50% neutralization GMTs in sera obtained 7 days after dose 2 in the Japanese trial (453 in 20–64-year-olds and 262 in 65–85-year-olds) were comparable to those of equivalent sera from the global clinical trial (361 in 18–55-year-olds and 149 in 65–85-year-olds)^9^. As has been previously reported with BNT162b2 and other vaccines^9,12,17^, vaccine immunogenicity decreased with age, with younger participants having higher SARS-CoV-2 50% neutralization GMTs than participants ≥65 years of age.

A limitation of the study is that safety, tolerability, and immunogenicity were not assessed in Japanese adolescents and children <20 years old. BNT162b2 has been shown to be safe and well tolerated in children as young as 5; studies in younger children are ongoing^12,14^. Immunogenicity data at 6 and 12 months after vaccination 2 are planned to be analyzed but are not yet available, as the study is ongoing. Efficacy was not assessed during this study, however, any reports of COVID-19 or SARS-CoV-2 have been collected and will be analyzed after study completion.

This study did not aim to determine the efficacy of BNT162b2 against COVID-19 in Japanese adults. The pivotal clinical trial reported vaccine efficacy of 95% among individuals ≥16 years of age beginning 7 days after dose 2^11^, and real-world data indicate that vaccine effectiveness after full immunization is 90–92% against COVID-19, and >97% against severe COVID-19^18–20^.

In summary, the safety, tolerability, and immunogenicity results reported here support the use of BNT162b2 to prevent COVID-19 in healthy Japanese adults.

## Methods

This randomized, placebo-controlled, observer-blind phase 1/2 study assessed BNT162b2 safety and immunogenicity in healthy Japanese adults (NCT04588480). This report presents data collected through the cut-off date of January 5, 2021; this is the primary analysis 1 month after dose 2 as planned in the protocol. Healthy Japanese adults 20–85 years of age, including those with stable preexisting disease, were included. Participants with known infection with hepatitis B virus or hepatitis C virus, or HIV, a history of severe allergic reactions associated with vaccination, with previous confirmed COVID-19, with diagnosis of an immunocompromising or immunodeficiency disorder, and those who were pregnant or breastfeeding were excluded. Receipt of medicines intended to prevent COVID-19, previous vaccination with any coronavirus vaccine, and treatment with immunosuppressive therapy were also exclusion criteria. Full inclusion and exclusion criteria are listed in the study protocol (Supplementary Information).

### Ethical conduct of the study

This study was conducted in accordance with the study protocol and principles derived from international guidelines including the International Council for Harmonisation Guidelines for Good Clinical Practice, the Declaration of Helsinki, and applicable laws and regulations including privacy laws. The study protocol, informed consent documents, and other relevant documents were prospectively approved by the institutional review board at Hakata Clinic. Written informed consent was obtained from all participants before enrollment and before participation in any study-related procedures.

### Study responsibilities

Pfizer was responsible for study design and conduct, data collection, analysis, and interpretation, and writing of this manuscript. Both Pfizer and BioNTech manufactured the study vaccine. BioNTech was the sponsor of the study and contributed to data interpretation and writing of the manuscript. All study data were available to all authors, who vouch for accuracy of the data and adherence of the study to the protocol.

### Procedures

Participants were recruited from healthy volunteer databases after written informed consent was obtained and the investigator confirmed eligibility according to the study protocol. Participants were randomized 3:1 (BNT162b2:placebo) using an interactive web-based response system to receive 2 intramuscular injections of 30 μg BNT162b2 or placebo (saline) 21 days apart. Participants, investigators and other study staff were blinded; staff who dispensed/administered study medication were unblinded. To evaluate vaccine-associated acute reactions, participants were observed at the study sites for 30 min after each vaccination. Study data were collected using InForm version 6.3 (Oracle, Texas, USA).

### Safety assessments

The primary safety objective was to describe the safety and tolerability of 2 doses of BNT162b2. Safety endpoints included assessment of reactogenicity (local reactions or systemic events for 7 days after each dose collected by electronic diary [e-diary; Trial Manager version 6.0, Signant Health, Pennsylvania, USA]), adverse events (AEs; reported by the participant without e-diary prompting) collected from dose 1 through 1 month after dose 2, and serious AEs (SAEs) collected from dose 1 through 12 months after dose 2 (reported to 1 month after dose 2 in this report). As this was the first study of BNT162b2 in Japan, hematology and clinical chemistry laboratory parameters up to 7 days after dose 2 were assessed in the first 24 participants (the clinical laboratory subset).

### Immunogenicity assessments

The primary immunogenicity objective was to describe the immune responses elicited by BNT162b2. The conduct of the severe acute respiratory syndrome coronavirus 2 (SARS-CoV-2) neutralization assay was reported previously^21^. The assay used a previously described strain of SARS-CoV-2 (USA_WA1/2020) that had been rescued by reverse genetics and engineered by the insertion of an mNeonGreen (*mNG*) gene into open reading frame 7 of the viral genome^22^. Compared with the wild-type virus, this reporter virus generates similar plaque morphologies and indistinguishable growth curves^21^. Viral master stocks used for the neutralization assay were grown in Vero (sans E6) cells. Serial dilutions of heat-inactivated sera were incubated with reporter virus for 1 h at 37 °C. Vero CCL81 cell monolayers were then inoculated in 96-well plates to allow accurate quantification of infected cells. Hoechst 33342 nuclear stain was used to enumerate total cell counts per well. Fluorescent virally infected foci were detected 16‒24 h after inoculation; the 50% neutralization titer was the interpolated reciprocal of the dilution yielding a 50% reduction in fluorescent viral foci^21^. Immunogenicity assessments were performed on sera obtained before doses 1 and 2, and at time points up to 1 month after dose 2. Primary immunogenicity endpoints were geometric mean titers (GMTs) of SARS-CoV-2 neutralization 1 month after dose 2, and geometric mean fold rises (GMFRs) of neutralizing titers from baseline to 1 month after dose 2. GMTs were derived by calculating the mean of the assay results after logarithm transformation, then exponentiating to express results on the original scale. Two-sided 95% CIs were obtained by natural log transformations of titers, calculating then exponentiating the 95% CI with reference to the Student’s *t* distribution. GMFRs were limited to participants with non-missing values before dose 1 and at the postvaccination time point. GMFRs were calculated as the mean of the difference of logarithmically transformed assay results (i.e., later time point − earlier time point) and exponentiation of the mean. Associated two-sided CIs were obtained using Student’s *t* distribution for the mean difference of the logarithmically transformed assay results and exponentiating the confidence limits. Secondary endpoints included GMTs of SARS-CoV-2 neutralization 7 and 14 days after dose 2, and associated GMFRs from before vaccination to each time point.

### Statistical analyses

The study size was not based on any formal hypothesis test.

The primary safety objective was evaluated by descriptive summary statistics for local reactions, systemic events, AEs, SAEs, and abnormal hematology and chemistry laboratory parameters for each vaccine group. In the primary safety objective evaluations, missing reactogenicity e-diary data were not imputed. Missing partial AE start dates were imputed. Safety endpoints are presented descriptively, with AEs and SAEs described according to terms in the Medical Dictionary for Regulatory Activities, version 23.1, for each group.

The primary immunogenicity objectives were evaluated descriptively by GMT, GMFR, and the associated 95% CIs for SARS-CoV-2 serum neutralizing titers 1 month after dose 2. Missing immunogenicity results were not imputed.

Data were analyzed using SAS version 9.4 (SAS Institute, Cary, NC).

### Reporting summary

Further information on research design is available in the Nature Research Reporting Summary linked to this article.

## Supplementary information

Supplementary Information

Reporting Summary

## Data Availability

Upon request, and subject to certain criteria, conditions, and exceptions (see https://www.pfizer.com/science/clinical-trials/trial-data-and-results for more information), Pfizer will provide access to individual de-identified participant data from Pfizer-sponsored global interventional clinical studies conducted for medicines, vaccines, and medical devices (1) for indications that have been approved in the US and/or EU or (2) in programs that have been terminated (i.e., development for all indications has been discontinued). Pfizer will also consider requests for the protocol, data dictionary, and statistical analysis plan. Data may be requested from Pfizer trials 24 months after study completion. The de-identified participant data will be made available to researchers whose proposals meet the research criteria and other conditions, and for which an exception does not apply, via a secure portal. To gain access, data requestors must enter into a data access agreement with Pfizer.
