# Supplementary material for: A randomized study to evaluate safety and immunogenicity of the BNT162b2 COVID-19 vaccine in healthy Japanese adults
Source: Nat Commun. 2021 Dec 14;12:7105. doi: 10.1038/s41467-021-27316-2 (PMC8671474; doi:10.1038/s41467-021-27316-2)
Supplement: Supplementary file 1 — Supplementary Information [file 41467_2021_27316_MOESM1_ESM.pdf]

A PHASE 1/2, PLACEBO-CONTROLLED, RANDOMIZED, AND OBSERVER-BLIND STUDY TO EVALUATE THE SAFETY, TOLERABILITY, AND IMMUNOGENICITY OF A SARS-COV-2 RNA VACCINE CANDIDATE AGAINST COVID-19 IN HEALTHY JAPANESE ADULTS

**Study Sponsor:**	BioNTech SE
**Study Conducted By:**	Pfizer Japan Inc
**Study Intervention Number:**	PF-07302048
**Study Intervention Name:**	RNA-Based COVID-19 Vaccine
**US IND Number:**	N/A
**EudraCT Number:**	N/A
**Protocol Number:**	C4591005
**Phase:**	1/2

Short Title: A Phase 1/2 Study to Evaluate the Safety, Tolerability, and Immunogenicity of an RNA Vaccine Candidate Against COVID-19 in Healthy Japanese Adults

This document and accompanying materials contain confidential information belonging to Pfizer. Except as otherwise agreed to in writing, by accepting or reviewing these documents, you agree to hold this information in confidence and not copy or disclose it to others (except where required by applicable law) or use it for unauthorized purposes. In the event of any actual or suspected breach of this obligation, Pfizer must be promptly notified.

090177e1968b9275\Approved\Approved On: 18-Mar-2021 07:58 (GMT)

Protocol Amendment Summary of Changes Table

**Document History**		
**Document**	**Version Date**	**Summary and Rationale for Changes**
Amendment 3	17 March 2021	• Transitioned this study to a postmarketing study according to the Japanese regulation.<br>• Added the possibility of administering BNT162b2 to participants who originally received placebo.<br>• Included the possibility, due to local circumstances related to the COVID-19 pandemic, that study procedures that do not require in-person participant contact may be performed by telehealth.<br>• Incorporated the PACL dated 20 November 2020.
Amendment 2	22 October 2020	• Removed S1-binding IgG assay and immunogenicity assessment from Sections 1.1, 3, 8.1, 9.2, 9.4.1, and 9.4.3, because these will not be included in analysis.<br>• Added in Section 6.3.3 that blinding can apply not just to designated CRAs but also to designated study managers to prevent potential risk of breaking the blind.
Amendment 1	17 September 2020	Following regulatory feedback:<br>• Extended the SAE reporting period to 12 months after the last dose of study intervention.<br>• Consolidated the reasons for discontinuation of study intervention.<br>• Revised an estimand of the primary safety objective to be consistent with the change in the SAE reporting period.<br>• Extended the period for collecting pregnancy details to be consistent with the change in the SAE reporting period.<br>In addition:<br>• Reflected the change in the SAE reporting period in other relevant sections.<br>• Added the number of participants in Section 4.1, because this was considered key information for the study design.
Original protocol	07 August 2020	N/A

This amendment incorporates all revisions to date, including amendments made at the request of country health authorities and IRBs/ECs.

090177e1968b9275\Approved\Approved On: 18-Mar-2021 07:58 (GMT)

TABLE OF CONTENTS

LIST OF TABLES 9
1. PROTOCOL SUMMARY 10
1.1. Synopsis 10
1.2. Schema 14
1.3. Schedule of Activities 15
1.3.1. Clinical Laboratory Subset: 16
1.3.2. Standard Participants 21
1.3.3. Administration of BNT162b2 to Those Originally Assigned to Placebo 24
2. INTRODUCTION 26
2.1. Study Rationale 26
2.2. Background 26
2.2.1. Clinical Overview 27
2.3. Benefit/Risk Assessment 27
2.3.1. Risk Assessment 28
2.3.2. Benefit Assessment 29
2.3.3. Overall Benefit/Risk Conclusion 29
3. OBJECTIVES, ESTIMANDS, AND ENDPOINTS 29
4. STUDY DESIGN 31
4.1. Overall Design 31
4.2. Scientific Rationale for Study Design 32
4.3. Justification for Dose 32
4.4. End of Study Definition 32
5. STUDY POPULATION 32
5.1. Inclusion Criteria 33
5.2. Exclusion Criteria 33
5.3. Lifestyle Considerations 35
5.3.1. Contraception 35
5.4. Screen Failures 35
5.5. Criteria for Temporarily Delaying Enrollment/Randomization/Study Intervention Administration 35

090177e1968b9275\Approved\Approved On: 18-Mar-2021 07:58 (GMT)

6. STUDY INTERVENTION 36
6.1. Study Intervention(s) Administered 36
6.1.1. Administration 37
6.2. Preparation/Handling/Storage/Accountability 37
6.2.1. Preparation and Dispensing 38
6.3. Measures to Minimize Bias: Randomization and Blinding....38
6.3.1. Allocation to Study Intervention 38
6.3.2. Blinding of Site Personnel 39
6.3.3. Blinding of the Sponsor 39
6.3.4. Breaking the Blind 40
6.4. Study Intervention Compliance....40
6.5. Concomitant Therapy 40
6.5.1. Prohibited During the Study 40
6.5.2. Permitted During the Study 41
6.6. Dose Modification....41
6.7. Intervention After the End of the Study 41
7. DISCONTINUATION OF STUDY INTERVENTION AND PARTICIPANT DISCONTINUATION/WITHDRAWAL....42
7.1. Discontinuation of Study Intervention 42
7.2. Participant Discontinuation/Withdrawal From the Study 42
7.2.1. Withdrawal of Consent....43
7.3. Lost to Follow-up 43
8. STUDY ASSESSMENTS AND PROCEDURES....44
8.1. Efficacy and/or Immunogenicity Assessments 45
8.1.1. Biological Samples 45
8.2. Safety Assessments 45
8.2.1. Clinical Safety Laboratory Assessments (Clinical Laboratory Subset Only) 46
8.2.2. Electronic Diary....46
8.2.2.1. Grading Scales....47
8.2.2.2. Local Reactions 47
8.2.2.3. Systemic Events 48

090177e1968b9275\Approved\Approved On: 18-Mar-2021 07:58 (GMT)

8.2.2.4. Fever 49
8.2.2.5. Antipyretic Medication 50
8.2.3. Surveillance of Events That Could Represent Enhanced COVID-19 Disease 50
8.2.4. Pregnancy Testing 50
8.3. Adverse Events and Serious Adverse Events 50
8.3.1. Time Period and Frequency for Collecting AE and SAE Information 51
8.3.1.1. Reporting SAEs to Pfizer Safety 52
8.3.1.2. Recording Nonserious AEs and SAEs on the CRF 52
8.3.2. Method of Detecting AEs and SAEs 52
8.3.3. Follow-up of AEs and SAEs 52
8.3.4. Regulatory Reporting Requirements for SAEs 52
8.3.5. Exposure During Pregnancy or Breastfeeding, and Occupational Exposure 53
8.3.5.1. Exposure During Pregnancy 53
8.3.5.2. Exposure During Breastfeeding 54
8.3.5.3. Occupational Exposure 55
8.3.6. Cardiovascular and Death Events 55
8.3.7. Disease-Related Events and/or Disease-Related Outcomes Not Qualifying as AEs or SAEs 55
8.3.8. Adverse Events of Special Interest 56
8.3.8.1. Lack of Efficacy 56
8.3.9. Medical Device Deficiencies 56
8.3.10. Medication Errors 56
8.4. Treatment of Overdose 57
8.5. Pharmacokinetics 57
8.6. Pharmacodynamics 57
8.7. Genetics 57
8.8. Biomarkers 58
8.9. Immunogenicity Assessments 58
8.10. Health Economics 58
8.11. Study Procedures 58

090177e1968b9275\Approved\Approved On: 18-Mar-2021 07:58 (GMT)

8.11.1. Clinical Laboratory Subset 58
8.11.1.1. Screening (0 to 28 Days Before Visit 1) 58
8.11.1.2. Visit 1 – Vaccination 1 (Day 1) 59
8.11.1.3. Visit 2 – Next-Day Follow-up Visit (Vaccination 1) (1 to 3 Days After Visit 1) 61
8.11.1.4. Visit 3 – 1-Week Follow-up Visit (Vaccination 1) (6 to 8 Days After Visit 1) 62
8.11.1.5. Visit 4 – Vaccination 2 (19 to 23 Days After Visit 1)....63
8.11.1.6. Visit 5 – 1-Week Follow-up Visit (6 to 8 Days After Visit 4) 65
8.11.1.7. Visit 6 – 2-Week Follow-up Visit (12 to 16 Days After Visit 4) 66
8.11.1.8. Visit 7 – 1-Month Follow-up Visit (28 to 35 Days After Visit 4) 67
8.11.1.9. Between Visits 7 and 8 67
8.11.1.10. Visit 8 – 6-Month Follow-up Visit (154 to 168 Days After Visit 4): Only for Those Participants Who Originally Received BNT162b2 or Placebo Recipients Who Decline BNT162b2 68
8.11.1.11. Visit 9 – 12-Month Follow-up Visit (350 to 378 Days After Visit 4): Only for Those Participants Who Originally Received BNT162b2 or Placebo Recipients Who Decline BNT162b2 68
8.11.2. Standard Participants 69
8.11.2.1. Visit 1 – Vaccination 1 (Day 1) 69
8.11.2.2. Visit 2 – Vaccination 2 (19 to 23 Days After Visit 1)....71
8.11.2.3. Visit 3 – 1-Week Follow-up Visit (6 to 8 Days After Visit 2) 73
8.11.2.4. Visit 4 – 2-Week Follow-up Visit (12 to 16 Days After Visit 2) 73
8.11.2.5. Visit 5 – 1-Month Follow-up Visit (28 to 35 Days After Visit 2) 74
8.11.2.6. Between Visits 5 and 6 75
8.11.2.7. Visit 6 – 6-Month Follow-up Visit (154 to 168 Days After Visit 2): Only for Those Participants Who Originally Received BNT162b2 or Placebo Recipients Who Decline BNT162b2 75

090177e1968b9275\Approved\Approved On: 18-Mar-2021 07:58 (GMT)

8.11.2.8. Visit 7 – 12-Month Follow-up Visit (350 to 378 Days After Visit 2): Only for Those Participants Who Originally Received BNT162b2 or Placebo Recipients Who Decline BNT162b2 75

8.12. Unscheduled Visit for a Grade 3 or Suspected Grade 4 Reaction 76

8.13. COVID-19 Disease Surveillance 77

8.13.1. Potential COVID-19 Illness Visit (Optimally Within 3 Days After Potential COVID-19 Illness Onset) 78

8.13.2. Potential COVID-19 Convalescent Visit (28 to 35 Days After Potential COVID-19 Illness Visit)....78

8.14. Communication and Use of Technology....78

8.15. SARS-CoV-2 NAAT Results From Vaccination 1 and Vaccination 2 Visits 79

8.16. Procedures for Administration of BNT162b2 to Those Originally Assigned to Placebo After Approval of Protocol Amendment 3 79

8.16.1. Visit 101 – Vaccination 3 (After Protocol Amendment 3 is Approved by the IRB)....80

8.16.2. Visit 102 – Vaccination 4 (19 to 23 Days After Visit 101)....81

8.16.3. Visit 103 – 1-Month After Vaccination 4 Follow-up Telephone Contact (28 to 35 Days After Visit 102)....82

8.16.4. Visit 104 – 6-Months After Vaccination 4 Follow-up Telephone Contact (175 to 189 Days After Visit 102)....83

9. STATISTICAL CONSIDERATIONS 83

9.1. Estimands and Statistical Hypotheses 83

9.1.1. Estimands....83

9.1.2. Statistical Hypotheses....84

9.2. Sample Size Determination....84

9.3. Analysis Sets 85

9.4. Statistical Analyses 85

9.4.1. Immunogenicity Analyses 86

9.4.2. Safety Analyses 88

9.5. Interim Analyses 89

9.5.1. Analysis Timing....89

9.6. Data Monitoring Committee or Other Independent Oversight Committee....90

090177e1968b9275\Approved\Approved On: 18-Mar-2021 07:58 (GMT)

10. SUPPORTING DOCUMENTATION AND OPERATIONAL CONSIDERATIONS 91
10.1. Appendix 1: Regulatory, Ethical, and Study Oversight Considerations 91
10.1.1. Regulatory and Ethical Considerations 91
10.1.1.1. Reporting of Safety Issues and Serious Breaches of the Protocol or ICH GCP 91
10.1.2. Financial Disclosure 92
10.1.3. Informed Consent Process 92
10.1.4. Data Protection 93
10.1.5. Dissemination of Clinical Study Data 93
10.1.6. Data Quality Assurance 95
10.1.7. Source Documents 96
10.1.8. Study and Site Start and Closure 96
10.1.9. Publication Policy 97
10.1.10. Sponsor's Qualified Medical Personnel 98
10.2. Appendix 2: Clinical Laboratory Tests 99
10.3. Appendix 3: Adverse Events: Definitions and Procedures for Recording, Evaluating, Follow-up, and Reporting 101
10.3.1. Definition of AE 101
10.3.2. Definition of SAE 102
10.3.3. Recording/Reporting and Follow-up of AEs and/or SAEs....104
10.3.4. Reporting of SAEs....108
10.4. Appendix 4: Contraceptive Guidance 109
10.4.1. Male Participant Reproductive Inclusion Criteria 109
10.4.2. Female Participant Reproductive Inclusion Criteria....109
10.4.3. Woman of Childbearing Potential 110
10.4.4. Contraception Methods....111
10.5. Appendix 5: Genetics 113
10.6. Appendix 6: Liver Safety: Suggested Actions and Follow-up Assessments 113
10.7. Appendix 7: Abbreviations 115
11. REFERENCES 118

090177e1968b9275\Approved\Approved On: 18-Mar-2021 07:58 (GMT)

LIST OF TABLES

Table 1. Local Reaction Grading Scale 48
Table 2. Systemic Event Grading Scale 49
Table 3. Scale for Fever 50
Table 4. Probability of Observing at Least 1 AE by Assumed True Event Rates 84
Table 5. Precision of Each SARS-CoV-2 Assay 85
Table 6. Laboratory Abnormality Grading Scale 99

090177e1968b9275\Approved\Approved On: 18-Mar-2021 07:58 (GMT)

1. PROTOCOL SUMMARY

1.1. Synopsis

Short Title: A Phase 1/2 Study to Evaluate the Safety, Tolerability, and Immunogenicity of an RNA Vaccine Candidate Against COVID-19 in Healthy Japanese Adults

Rationale

A pneumonia of unknown cause detected in Wuhan, China, was first reported in December 2019. On 08 January 2020, the pathogen causing this outbreak was identified as a novel coronavirus. The outbreak was declared a Public Health Emergency of International Concern on 30 January 2020. On 12 February 2020, the virus was officially named as SARS-CoV-2, and the WHO officially named the disease caused by SARS-CoV-2 as COVID-19. On 11 March 2020, the WHO upgraded the status of the COVID-19 outbreak from epidemic to pandemic, which is now spreading globally at high speed.

There are currently no licensed vaccines to prevent infection with SARS-CoV-2 or COVID-19. Given the rapid transmission of COVID-19 and incidence of disease globally, including Japan, the rapid development of an effective vaccine is of utmost importance.

BioNTech has developed RNA-based vaccine candidates using a platform approach that enables the rapid development of vaccines against emerging viral diseases, including SARS-CoV-2. Each vaccine candidate is based on a platform of nucleoside-modified messenger RNA (modRNA, BNT162b). Each vaccine candidate expresses 1 of 2 antigens: the SARS-CoV-2 full-length, P2 mutant, prefusion spike glycoprotein (P2 S) (version 9) (BNT162b2 [variant RBP020.2]: a modRNA encoding P2 S) or a trimerized SARS-CoV-2 spike glycoprotein receptor-binding domain (RBD) (version 5) (BNT162b1 [variant RBP020.3]: a modRNA encoding the RBD).

A Phase 1/2/3 study (C4591001) conducted in the United States in adults 18 to 85 years of age is intended to investigate the safety, immunogenicity, and efficacy of these prophylactic BNT162 vaccines against COVID-19. The 2 vaccine candidates (BNT162b1 and BNT162b2) at several dose levels were studied in the C4591001 study, and the vaccine candidate selected for Phase 2/3 evaluation in the C4591001 study is BNT162b2 at a dose of 30 µg.

This study is a Phase 1/2 study to evaluate the safety, tolerability, and immunogenicity of the BNT162b2 vaccine candidate selected based on the data from the C4591001 study, in Japanese adults 20 to 85 years of age.

090177e1968b9275\Approved\Approved On: 18-Mar-2021 07:58 (GMT)

Objectives, Estimands, and Endpoints

Objectives	Estimands	Endpoints
**Primary Safety:**		
To describe the safety and tolerability profiles of a prophylactic BNT162 vaccine in healthy Japanese adults after 2 doses	In participants receiving at least 1 dose of study intervention, the percentage of participants reporting:<br>• Local reactions for up to 7 days following each dose<br>• Systemic events for up to 7 days following each dose<br>• AEs from Dose 1 to 1 month after Dose 2<br>• SAEs from Dose 1 to 12 months after Dose 2<br>In addition, in a clinical laboratory subset, the percentage of participants with:<br>• Abnormal hematology and chemistry laboratory values 1 and 7 days after Dose 1; and 7 days after Dose 2<br>• Grading shifts in hematology and chemistry laboratory assessments between baseline and 1 and 7 days after Dose 1; and before Dose 2 and 7 days after Dose 2	• Local reactions (pain at the injection site, redness, and swelling)<br>• Systemic events (fever, fatigue, headache, chills, vomiting, diarrhea, new or worsened muscle pain, and new or worsened joint pain)<br>• AEs<br>• SAEs<br>• Hematology and chemistry laboratory parameters detailed in Section 10.2
**Primary Immunogenicity:**		
To describe the immune responses elicited by a prophylactic BNT162 vaccine in healthy Japanese adults	In participants complying with the key protocol criteria (evaluable participants):<br>• GMTs 1 month after Dose 2<br>• GMFR from before vaccination to 1 month after Dose 2	• SARS-CoV-2 serum neutralizing titers
**Secondary Immunogenicity:**		
To describe the immune responses elicited by a prophylactic BNT162 vaccine in healthy Japanese adults	In the evaluable participants, at baseline, 21 days after Dose 1; 7 and 14 days and 6 and 12 months after Dose 2:<br>• GMTs at each time point<br>• GMFR from before vaccination to each subsequent time point after vaccination	• SARS-CoV-2 serum neutralizing titers

090177e1968b9275\Approved\Approved On: 18-Mar-2021 07:58 (GMT)

Objectives	Estimands	Endpoints
To describe the immune responses elicited by a prophylactic BNT162 vaccine in participants with/without confirmed COVID-19 before Dose 2	In the evaluable participants, at baseline, 21 days after Dose 1; 7 and 14 days and 1, 6, and 12 months after Dose 2: • GMTs at each time point • GMFR from before vaccination to each subsequent time point after vaccination	• SARS-CoV-2 serum neutralizing titers
**Exploratory Immunogenicity:**		
To describe the immune responses elicited by a prophylactic BNT162 vaccine in participants with/without confirmed COVID-19 during the study	In the evaluable participants, at baseline, 21 days after Dose 1; 7 and 14 days and 1, 6, and 12 months after Dose 2: • GMTs at each time point • GMFR from before vaccination to each subsequent time point after vaccination	• SARS-CoV-2 serum neutralizing titers
To evaluate the immune response (non-S) to SARS-CoV-2 in participants with/without confirmed COVID-19 during the study		• SARS-CoV-2 N-binding antibody

Overall Design

This is a Phase 1/2, randomized, placebo-controlled, and observer-blind study in healthy Japanese adults.

The study will evaluate the safety, tolerability, and immunogenicity of the SARS-CoV-2 RNA vaccine candidate against COVID-19 (BNT162b2), which was the same as the vaccine candidate selected for Phase 2/3 evaluation in the C4591001 study:

- As 2 doses, separated by 21 days
- At a 30-µg dose level
- In adults 20 to 85 years of age

Local reactions (redness, swelling, and pain at the injection site), systemic events (fever, vomiting, diarrhea, headache, fatigue, chills, new or worsened muscle pain, and new or worsened joint pain), and use of antipyretic medication will be prompted for and collected by all participants in an e-diary from Day 1 through Day 7 after each administration of study intervention. AEs will be collected from the time the participant provides informed consent

090177e1968b9275\Approved\Approved On: 18-Mar-2021 07:58 (GMT)

through 1 month after Dose 2, and SAEs will be collected from the time of informed consent through 12 months after Dose 2.

As this study is the first study conducted in Japan, clinical laboratory tests will be performed in the first 24 participants (12 participants 20 to 64 years of age and 12 participants 65 to 85 years of age) (clinical laboratory subset). After randomization of all participants in the clinical laboratory subset is completed, the other participants (standard participants) will be enrolled.

Blood will be collected prior to Dose 1, prior to Dose 2, and 7 and 14 days and 1, 6, and 12 months after Dose 2 to assess immunogenicity.

The study is observer-blinded, as the physical appearance of the investigational vaccine candidates and the placebo may differ. The participant, investigator, study coordinator, and other site staff will be blinded. At the study site, only the dispenser(s)/administrator(s) are unblinded.

To facilitate rapid review of data in real time, sponsor staff will be unblinded to vaccine allocation for the participants.

From protocol amendment 3, this study is transitioned from a clinical trial to a postmarketing study according to the Japanese regulation, because BNT162b2 was approved by the MHLW on 14 February 2021.

All participants who originally received placebo will be offered the opportunity to receive BNT162b2 at their earliest convenience as part of the study (administration of BNT162b2 before the 6-month follow-up visit [Visit 8 for the clinical laboratory subset or Visit 6 for standard participants] is allowed).

Number of Participants

One hundred sixty participants will be randomly assigned in a 3:1 ratio to study intervention (candidate vaccine: 120, placebo: 40) such that approximately 144 evaluable participants (candidate vaccine: 108, placebo: 36) will be evaluated.

Intervention Groups and Duration

The study will evaluate a 2-dose (separated by 21 days) schedule of 30 μg of the investigational RNA vaccine candidate for active immunization against COVID-19, which is the same as that selected for Stage 3 of the C4591001 study. The vaccine candidate or placebo (normal saline) will be administered to a study participant.

Participants are expected to participate for up to a maximum of approximately 14 months.

090177e1968b9275\Approved\Approved On: 18-Mar-2021 07:58 (GMT)

Data Monitoring Committee or Other Independent Oversight Committee: Yes

An external DMC will be utilized for this study and will review cumulative unblinded safety data throughout the study in accordance with the charter.

Statistical Methods

The study sample size is not based on any statistical hypothesis testing. All statistical analyses of safety and immunogenicity will be descriptive.

The primary safety objective will be evaluated by descriptive summary statistics for local reactions, systemic events, AEs/SAEs, and abnormal hematology and chemistry laboratory parameters (clinical laboratory subset only), for each vaccine group. A 3-tier approach will be used to summarize AEs.

The primary immunogenicity objectives will be evaluated descriptively by GMT, GMFR, and the associated 95% CIs for SARS-CoV-2 serum neutralizing titers at 1 month after Dose 2.

Additionally, the RCDCs of SARS-CoV-2 serum neutralizing titers and SARS-CoV-2 N-binding antibody will be provided for the specified time points to provide further characterization of the immune response.

1.2. Schema

Not applicable.

090177e1968b9275\Approved\Approved On: 18-Mar-2021 07:58 (GMT)

1.3. Schedule of Activities

The SoA table provides an overview of the protocol visits and procedures. Refer to the STUDY ASSESSMENTS AND PROCEDURES section of the protocol for detailed information on each procedure and assessment required for compliance with the protocol.

The investigator may schedule visits (unplanned visits) in addition to those listed in the SoA table, in order to conduct evaluations or assessments required to protect the well-being of the participant.

Administration of BNT162b2 to Those Originally Assigned to Placebo: All participants will be informed of their original study intervention allocation (BNT162b2 or placebo) by the site staff. All participants who originally received placebo will be offered the opportunity to receive BNT162b2 at their earliest convenience as part of the study (administration of BNT162b2 before the 6-month follow-up visit [Visit 8 for the clinical laboratory subset or Visit 6 for the standard participants] is allowed). If they want to receive BNT162b2, they will move to the SoA in Section 1.3.3 for their remaining visits. All participants who originally received BNT162b2 and placebo recipients who decline BNT162b2 vaccination will continue in the study as originally planned.

090177e1968b9275\Approved\Approved On: 18-Mar-2021 07:58 (GMT)

090177e1968b9275\Approved\Approved On: 18-Mar-2021 07:58 (GMT)

1.3.1. Clinical Laboratory Subset:

Visit Number	Screening	1	2	3	4	5	6	7	8	9	Unplanned	Unplanned
**Visit Description**	**Screening**	**Vax 1**	**Next-Day Follow-up Visit (Vax 1)**	**1-Week Follow-up Visit (Vax 1)**	**Vax 2**	**1-Week Follow-up Visit (Vax 2)**	**2-Week Follow-up Visit (Vax 2)**	**1-Month Follow-up Visit**	**6-Month Follow-up Visit**	**12-Month Follow-up Visit**	**Potential COVID-19 Illness Visit[a]**	**Potential COVID-19 Convalescent Visit[a]**
**Visit Window (Days)**	**0 to 28 Days Before Visit 1**	**Day 1**	**1 to 3 Days After Visit 1**	**6 to 8 Days After Visit 1**	**19 to 23 Days After Visit 1**	**6 to 8 Days After Visit 4**	**12 to 16 Days After Visit 4**	**28 to 35 Days After Visit 4**	**154 to 168 Days After Visit 4**	**350 to 378 Days After Visit 4**	**Optimally Within 3 Days After Potential COVID-19 Illness Onset**	**28 to 35 Days After Potential COVID-19 Illness Visit**
									**ONLY FOR THOSE PARTICIPANTS ORIGINALLY ASSIGNED TO BNT162b2 OR PLACEBO RECIPIENTS WHO DECLINE BNT162b2**			
Obtain informed consent	X											
Assign participant number	X											
Obtain demography and medical history data	X											
Perform clinical assessment[b]	X	X	X	X	X							
Measure height and weight	X											
Collect blood sample for hematology and chemistry laboratory tests[c]	~10 mL		~10 mL	~10 mL	~10 mL	~10 mL						
Perform urine pregnancy test (if appropriate)[d]	X	X			X							
Collect nonstudy vaccine information	X	X	X	X	X	X	X	X	X			

**Visit Number**	**Screening**	**1**	**2**	**3**	**4**	**5**	**6**	**7**	**8**	**9**	**Unplanned**	**Unplanned**
**Visit Description**	**Screening**	**Vax 1**	**Next-Day Follow-up Visit (Vax 1)**	**1-Week Follow-up Visit (Vax 1)**	**Vax 2**	**1-Week Follow-up Visit (Vax 2)**	**2-Week Follow-up Visit (Vax 2)**	**1-Month Follow-up Visit**	**6-Month Follow-up Visit**	**12-Month Follow-up Visit**	**Potential COVID-19 Illness Visit[a]**	**Potential COVID-19 Convalescent Visit[a]**
**Visit Window (Days)**	**0 to 28 Days Before Visit 1**	**Day 1**	**1 to 3 Days After Visit 1**	**6 to 8 Days After Visit 1**	**19 to 23 Days After Visit 1**	**6 to 8 Days After Visit 4**	**12 to 16 Days After Visit 4**	**28 to 35 Days After Visit 4**	**154 to 168 Days After Visit 4**	**350 to 378 Days After Visit 4**	**Optimally Within 3 Days After Potential COVID-19 Illness Onset**	**28 to 35 Days After Potential COVID-19 Illness Visit**
									**ONLY FOR THOSE PARTICIPANTS ORIGINALLY ASSIGNED TO BNT162b2 OR PLACEBO RECIPIENTS WHO DECLINE BNT162b2**			
Confirm prohibited medication use			X	X	X	X	X	X	X	X	X	X
Confirm eligibility	X	X			X							
Review hematology and chemistry results		X		X	X	X	X					
Measure temperature (axillary)		X			X							
Review temporary delay criteria		X			X							
Confirm use of contraceptives (if appropriate)	X	X	X	X	X	X	X	X				
Obtain randomization number and study intervention allocation		X										
Collect blood sample for immunogenicity assessment[d]		~25 mL			~25 mL	~25 mL	~25 mL	~25 mL	~25 mL	~25 mL		
Obtain nasal (midturbinate) swab[d]		X			X							

090177e1968b9275\Approved\Approved On: 18-Mar-2021 07:58 (GMT)

Visit Number	Screening	1	2	3	4	5	6	7	8	9	Unplanned	Unplanned
**Visit Description**	**Screening**	**Vax 1**	**Next-Day Follow-up Visit (Vax 1)**	**1-Week Follow-up Visit (Vax 1)**	**Vax 2**	**1-Week Follow-up Visit (Vax 2)**	**2-Week Follow-up Visit (Vax 2)**	**1-Month Follow-up Visit**	**6-Month Follow-up Visit**	**12-Month Follow-up Visit**	**Potential COVID-19 Illness Visit[a]**	**Potential COVID-19 Convalescent Visit[a]**
**Visit Window (Days)**	**0 to 28 Days Before Visit 1**	**Day 1**	**1 to 3 Days After Visit 1**	**6 to 8 Days After Visit 1**	**19 to 23 Days After Visit 1**	**6 to 8 Days After Visit 4**	**12 to 16 Days After Visit 4**	**28 to 35 Days After Visit 4**	**154 to 168 Days After Visit 4**	**350 to 378 Days After Visit 4**	**Optimally Within 3 Days After Potential COVID-19 Illness Onset**	**28 to 35 Days After Potential COVID-19 Illness Visit**
									**ONLY FOR THOSE PARTICIPANTS ORIGINALLY ASSIGNED TO BNT162b2 OR PLACEBO RECIPIENTS WHO DECLINE BNT162b2**			
Administer study intervention		X			X							
Assess acute reactions for at least 30 minutes after study intervention administration		X			X							
Explain participant communication methods (including for e-diary completion), assist the participant with downloading the app, or issue provisioned device, if required		X										
Provide thermometer and measuring device		X			X							
Review reactogenicity e-diary data (daily review is optimal during the active diary period)		◄	—	►	◄	►						

090177e1968b9275\Approved\Approved On: 18-Mar-2021 07:58 (GMT)

090177e1968b9275\Approved\Approved On: 18-Mar-2021 07:58 (GMT)

Visit Number	Screening	1	2	3	4	5	6	7	8	9	Unplanned	Unplanned
**Visit Description**	**Screening**	**Vax 1**	**Next-Day Follow-up Visit (Vax 1)**	**1-Week Follow-up Visit (Vax 1)**	**Vax 2**	**1-Week Follow-up Visit (Vax 2)**	**2-Week Follow-up Visit (Vax 2)**	**1-Month Follow-up Visit**	**6-Month Follow-up Visit**	**12-Month Follow-up Visit**	**Potential COVID-19 Illness Visit[a]**	**Potential COVID-19 Convalescent Visit[a]**
**Visit Window (Days)**	**0 to 28 Days Before Visit 1**	**Day 1**	**1 to 3 Days After Visit 1**	**6 to 8 Days After Visit 1**	**19 to 23 Days After Visit 1**	**6 to 8 Days After Visit 4**	**12 to 16 Days After Visit 4**	**28 to 35 Days After Visit 4**	**154 to 168 Days After Visit 4**	**350 to 378 Days After Visit 4**	**Optimally Within 3 Days After Potential COVID-19 Illness Onset**	**28 to 35 Days After Potential COVID-19 Illness Visit**
									**ONLY FOR THOSE PARTICIPANTS ORIGINALLY ASSIGNED TO BNT162b2 OR PLACEBO RECIPIENTS WHO DECLINE BNT162b2**			
Review ongoing reactogenicity e-diary symptoms and obtain stop dates					X	X	X					
Collect AEs and SAEs as appropriate[e]	X	X	X	X	X	X	X	X	X	X	X	X
Collect e-diary or assist the participant to delete application										X		

Visit Number	Screening	1	2	3	4	5	6	7	8	9	Unplanned	Unplanned
**Visit Description**	**Screening**	**Vax 1**	**Next-Day Follow-up Visit (Vax 1)**	**1-Week Follow-up Visit (Vax 1)**	**Vax 2**	**1-Week Follow-up Visit (Vax 2)**	**2-Week Follow-up Visit (Vax 2)**	**1-Month Follow-up Visit**	**6-Month Follow-up Visit**	**12-Month Follow-up Visit**	**Potential COVID-19 Illness Visit[a]**	**Potential COVID-19 Convalescent Visit[a]**
**Visit Window (Days)**	**0 to 28 Days Before Visit 1**	**Day 1**	**1 to 3 Days After Visit 1**	**6 to 8 Days After Visit 1**	**19 to 23 Days After Visit 1**	**6 to 8 Days After Visit 4**	**12 to 16 Days After Visit 4**	**28 to 35 Days After Visit 4**	**154 to 168 Days After Visit 4**	**350 to 378 Days After Visit 4**	**Optimally Within 3 Days After Potential COVID-19 Illness Onset**	**28 to 35 Days After Potential COVID-19 Illness Visit**
									**ONLY FOR THOSE PARTICIPANTS ORIGINALLY ASSIGNED TO BNT162b2 OR PLACEBO RECIPIENTS WHO DECLINE BNT162b2**			
Collection of local test result of COVID-19 and local diagnosis of COVID-19											X	

Abbreviation: vax = vaccination.

a. The COVID-19 illness visit may be conducted as a telehealth visit.
b. Including, if indicated, a physical examination.
c. Hematology: hemoglobin, hematocrit, complete blood count with differential, and platelets. Blood chemistry: alanine aminotransferase (ALT), aspartate aminotransferase (AST), alkaline phosphatase, total bilirubin, blood urea nitrogen (BUN), and creatinine.
d. Prior to vaccination, if at a vaccination visit.
e. Details of AE and SAE reporting are described in Section 8.3.1.

090177e1968b9275\Approved\Approved On: 18-Mar-2021 07:58 (GMT)

090177e1968b9275\Approved\Approved On: 18-Mar-2021 07:58 (GMT)

1.3.2. Standard Participants

**Visit Number**	**1**	**2**	**3**	**4**	**5**	**6**	**7**	**Unplanned**	**Unplanned**
**Visit Description**	**Vaccination 1**	**Vaccination 2**	**1-Week Follow-up Visit**	**2-Week Follow-up Visit**	**1-Month Follow-up Visit**	**6-Month Follow-up Visit**	**12-Month Follow-up Visit**	**Potential COVID-19 Illness Visit**[a]	**Potential COVID-19 Convalescent Visit**[a]
**Visit Window (Days)**	**Day 1**	**19 to 23 Days After Visit 1**	**6 to 8 Days After Visit 2**	**12 to 16 Days After Visit 2**	**28 to 35 Days After Visit 2**	**154 to 168 Days After Visit 2**	**350 to 378 Days After Visit 2**	**Optimally Within 3 Days After Potential COVID-19 Illness Onset**	**28 to 35 Days After Potential COVID-19 Illness Visit**
						**ONLY FOR THOSE PARTICIPANTS ORIGINALLY ASSIGNED TO BNT162b2 OR PLACEBO RECIPIENTS WHO DECLINE BNT162b2**			
Obtain informed consent	X								
Assign participant number	X								
Obtain demography and medical history data	X								
Perform clinical assessment[b]	X								
Measure height and weight	X								
Perform urine pregnancy test (if appropriate)[c]	X	X							
Collect nonstudy vaccine information	X	X	X	X	X	X			
Confirm prohibited medication use		X	X	X	X	X	X	X	X
Confirm eligibility	X	X							
Measure temperature (axillary)	X	X							
Review temporary delay criteria	X	X							
Confirm use of contraceptives (if appropriate)	X	X	X	X	X				
Obtain randomization number and study intervention allocation	X								

Visit Number	1	2	3	4	5	6	7	Unplanned	Unplanned
**Visit Description**	**Vaccination 1**	**Vaccination 2**	**1-Week Follow-up Visit**	**2-Week Follow-up Visit**	**1-Month Follow-up Visit**	**6-Month Follow-up Visit**	**12-Month Follow-up Visit**	**Potential COVID-19 Illness Visit[a]**	**Potential COVID-19 Convalescent Visit[a]**
**Visit Window (Days)**	**Day 1**	**19 to 23 Days After Visit 1**	**6 to 8 Days After Visit 2**	**12 to 16 Days After Visit 2**	**28 to 35 Days After Visit 2**	**154 to 168 Days After Visit 2**	**350 to 378 Days After Visit 2**	**Optimally Within 3 Days After Potential COVID-19 Illness Onset**	**28 to 35 Days After Potential COVID-19 Illness Visit**
						**ONLY FOR THOSE PARTICIPANTS ORIGINALLY ASSIGNED TO BNT162b2 OR PLACEBO RECIPIENTS WHO DECLINE BNT162b2**			
Collect blood sample for immunogenicity assessment[c]	~25 mL	~25 mL	~25 mL	~25 mL	~25 mL	~25 mL	~25 mL		
Obtain nasal (midturbinate) swab[c]	X	X							
Administer study intervention	X	X							
Assess acute reactions for at least 30 minutes after study intervention administration	X	X							
Explain participant communication methods (including for e-diary completion), assist the participant with downloading the app, or issue provisioned device, if required	X								
Provide a thermometer (all participants) and measuring device	X	X							
Review reactogenicity e-diary data (daily review is optimal during the active diary period)	←→	←——	——→						
Review ongoing reactogenicity e-diary symptoms and obtain stop dates		X	X	X					
Collect AEs and SAEs as appropriate[d]	X	X	X	X	X	X	X	X	X

090177e1968b9275\Approved\Approved On: 18-Mar-2021 07:58 (GMT)

090177e1968b9275\Approved\Approved On: 18-Mar-2021 07:58 (GMT)

**Visit Number**	**1**	**2**	**3**	**4**	**5**	**6**	**7**	**Unplanned**	**Unplanned**
**Visit Description**	**Vaccination 1**	**Vaccination 2**	**1-Week Follow-up Visit**	**2-Week Follow-up Visit**	**1-Month Follow-up Visit**	**6-Month Follow-up Visit**	**12-Month Follow-up Visit**	**Potential COVID-19 Illness Visit[a]**	**Potential COVID-19 Convalescent Visit[a]**
**Visit Window (Days)**	**Day 1**	**19 to 23 Days After Visit 1**	**6 to 8 Days After Visit 2**	**12 to 16 Days After Visit 2**	**28 to 35 Days After Visit 2**	**154 to 168 Days After Visit 2**	**350 to 378 Days After Visit 2**	**Optimally Within 3 Days After Potential COVID-19 Illness Onset**	**28 to 35 Days After Potential COVID-19 Illness Visit**
						**ONLY FOR THOSE PARTICIPANTS ORIGINALLY ASSIGNED TO BNT162b2 OR PLACEBO RECIPIENTS WHO DECLINE BNT162b2**			
Collect e-diary or assist the participant to delete application							X		
Collection of local test result of COVID-19 and local diagnosis of COVID-19								X	

a. The COVID-19 illness visit may be conducted as a telehealth visit.
b. Including, if indicated, a physical examination.
c. Prior to vaccination, if at a vaccination visit.
d. Details of AE and SAE reporting are described in Section 8.3.1.

090177e1968b9275\Approved\Approved On: 18-Mar-2021 07:58 (GMT)

1.3.3. Administration of BNT162b2 to Those Originally Assigned to Placebo

All participants who originally received placebo will be offered the opportunity to receive BNT162b2 at their earliest convenience as part of the study (administration of BNT162b2 before the 6-month follow-up visit [Visit 8 for the clinical laboratory subset or Visit 6 for standard participants] is allowed).

Visit Number	101	102	103	104	Unplanned	Unplanned
Visit Description	Vaccination 3	Vaccination 4	1-Month Telephone Contact	6-Month Telephone Contact	Potential COVID-19 Illness Visit[a]	Potential COVID-19 Convalescent Visit[a]
Visit Window (Days)	After This Protocol Amendment is Approved by the IRB[b]	19 to 23 Days After Visit 101	28 to 35 Days After Visit 102	175 to 189 Days After Visit 102	Optimally Within 3 Days After Potential COVID-19 Illness Onset	28 to 35 Days After Potential COVID-19 Illness Visit
Obtain informed consent	X					
Confirm participant originally received placebo	X					
Perform urine pregnancy test (if appropriate)	X	X				
Confirm use of contraceptives (if appropriate)	X	X				
Collect nonstudy vaccine information	X	X	X	X		
Confirm prohibited medication use	X	X	X	X	X	X
Confirm eligibility	X	X				
Measure temperature (axillary)	X	X				
Review temporary delay criteria	X	X				
Collect blood sample for immunogenicity assessment[c]	~25 mL					
Obtain vaccine vial allocation via IRT	X	X				
Administer BNT162b2	X	X				
Assess acute reactions for at least 30 minutes after study intervention administration	X	X				
Collect AEs and SAEs as appropriate[d]	X	X	X	X	X	X
Contact the participant by telephone[e]			X	X		
Request the participant return the e-diary or assist the participant to delete the application				X		

090177e1968b9275\Approved\Approved On: 18-Mar-2021 07:58 (GMT)

Visit Number	101	102	103	104	Unplanned	Unplanned
**Visit Description**	**Vaccination 3**	**Vaccination 4**	**1-Month Telephone Contact**	**6-Month Telephone Contact**	**Potential COVID-19 Illness Visit[a]**	**Potential COVID-19 Convalescent Visit[a]**
**Visit Window (Days)**	**After This Protocol Amendment is Approved by the IRB[b]**	**19 to 23 Days After Visit 101**	**28 to 35 Days After Visit 102**	**175 to 189 Days After Visit 102**	**Optimally Within 3 Days After Potential COVID-19 Illness Onset**	**28 to 35 Days After Potential COVID-19 Illness Visit**
Collection of local test result of COVID-19 and local diagnosis of COVID-19					X	

Abbreviations: IRB = institutional review board; IRT = interactive response technology.

a. The COVID 19 illness visit may be conducted as a telehealth visit.
b. For all placebo recipients who wish to receive BNT162b2; may be combined with Visit 8 for the clinical laboratory subset or Visit 6 for the standard participants.
c. Only if the participant has no blood sample collected in the previous 7 days.
d. Nonserious AEs need only be recorded if the participant remains in the AE reporting period (see Section 8.3.1).
e. Visit is intended to be conducted by telephone but, if desired, may be conducted in person.

2. INTRODUCTION

The BNT162 RNA-based COVID-19 vaccines are currently being investigated for prevention of COVID-19 in healthy adults.

2.1. Study Rationale

The purpose of the study is to rapidly describe the safety, tolerability, and immunogenicity of BNT162 RNA-based COVID-19 vaccine candidate against COVID-19 in healthy Japanese adults. There are currently no licensed vaccines to prevent infection with SARS-CoV-2 or COVID-19. Given the global crisis of COVID-19 and fast expansion of the disease globally, including Japan, the rapid development of an effective vaccine is of utmost importance.

2.2. Background

In December 2019, a pneumonia outbreak of unknown cause occurred in Wuhan, China. In January 2020, it became clear that a 2019-nCoV was the underlying cause. Later in January, the genetic sequence of the 2019-nCoV became available to the World Health Organization (WHO) and public (MN908947.3), and the virus was categorized in the *Betacoronavirus* subfamily. By sequence analysis, the phylogenetic tree revealed a closer relationship to severe acute respiratory syndrome (SARS) virus isolates than to another coronavirus infecting humans, the Middle East respiratory syndrome (MERS) virus.

The outbreak was declared a Public Health Emergency of International Concern on 30 January 2020. On 12 February 2020, the virus was officially named as SARS-CoV-2, and the WHO officially named the disease caused by SARS-CoV-2 as coronavirus disease 2019 (COVID-19).

SARS-CoV-2 infections and the resulting disease, COVID-19, have spread globally, affecting a growing number of countries.

On 11 March 2020, the WHO characterized the COVID-19 outbreak as a pandemic.[1] The WHO Situation Update Report dated 30 March 2020 noted 693,224 confirmed cases with 33,106 deaths globally, including 142,081 confirmed cases with 2457 deaths in the Americas.[2] The number of confirmed cases continues to rise globally. There are currently no licensed vaccines to prevent infection with SARS-CoV-2 or COVID-19.[3]

A prophylactic, RNA-based SARS-CoV-2 vaccine provides one of the most flexible and fastest approaches available to immunize against the emerging virus.[4,5] BioNTech has developed RNA-based vaccine candidates using a platform approach that enables the rapid development of vaccines against emerging viral diseases, including SARS CoV-2.

The development of an RNA-based vaccine encoding a viral antigen, which is then expressed by the vaccine recipient as a protein capable of eliciting protective immune responses, provides significant advantages over more traditional vaccine approaches. Unlike live attenuated vaccines, RNA vaccines do not carry the risks associated with infection and may be given to people who cannot be administered live virus (eg, pregnant women and immunocompromised persons). RNA-based vaccines are manufactured via a cell-free

090177e1968b9275\Approved\Approved On: 18-Mar-2021 07:58 (GMT)

in vitro transcription process, which allows an easy and rapid production and the prospect of producing high numbers of vaccination doses within a shorter time period than achieved with traditional vaccine approaches. This capability is pivotal to enable the most effective response in outbreak scenarios.

Two SARS-CoV-2–RNA lipid nanoparticle (RNA-LNP) vaccines based on a platform of nucleoside-modified messenger RNA (modRNA, BNT162b) will be evaluated in a Phase 1/2/3 study (C4591001) conducted in the United States. Each vaccine candidate expresses 1 of 2 antigens: the SARS-CoV-2 full-length, P2 mutant, prefusion spike glycoprotein (P2 S) (version 9) (BNT162b2 [variant RBP020.2]: a modRNA encoding P2 S) or a trimerized SARS-CoV-2 spike glycoprotein-receptor binding domain (RBD) (version 5) (BNT162b1 [variant RBP020.3]: a modRNA encoding the RBD). The vaccine candidate selected for Phase 2/3 evaluation in the C4591001 study is BNT162b2.

This study will evaluate the safety, tolerability, and immunogenicity of a vaccine candidate (BNT162b2) in Japanese adults 20 to 85 years of age.

2.2.1. Clinical Overview

Prior to this study, given clinical data from other similarly formulated uRNA liposomal vaccines from BioNTech in oncology trials[6] and recent published results from clinical trials using modRNA influenza vaccines by Moderna,[7] the BNT162 vaccines were expected to have a favorable safety profile with mild, localized, and transient effects. BNT162 vaccines based on modRNA have now been administered to humans for the first time in the C4591001 study and the BNT162-01 study conducted in Germany by BioNTech, at doses between 1 µg and 100 µg. The currently available safety and immunogenicity data are presented in the BNT162 IB.

2.3. Benefit/Risk Assessment

There is an ongoing global pandemic of COVID-19 with no preventive or therapeutic options available. There are accumulating data from ongoing human clinical trials of BNT162 vaccines in the United States and Germany, with an acceptable safety and tolerability profile observed at the time of initiation of this study. In addition, available nonclinical data with these vaccines, and data from nonclinical studies and clinical trials with the same or related RNA components, or antigens, supported a favorable risk/benefit profile. Anticipated AEs after vaccination were expected to be manageable using routine symptom-driven standard of care as determined by the investigators and, as a result, the profile of these vaccine candidates supported initiation of this Phase 1/2 clinical study.

More detailed information about the known and expected benefits and risks and reasonably expected AEs of BNT162 RNA-based COVID-19 vaccines may be found in the IB, which is the SRSD for this study.

090177e1968b9275\Approved\Approved On: 18-Mar-2021 07:58 (GMT)

090177e1968b9275\Approved\Approved On: 18-Mar-2021 07:58 (GMT)

2.3.1. Risk Assessment

Potential Risk of Clinical Significance	Summary of Data/Rationale for Risk	Mitigation Strategy
**Study Intervention: BNT162 RNA-Based COVID-19 Vaccine**		
Potential for local reactions (injection site redness, injection site swelling, and injection site pain) and systemic events (fever, fatigue, headache, chills, vomiting, diarrhea, muscle pain, and joint pain) following vaccination.	These are common adverse reactions seen with other vaccines, as noted in the FDA CBER guidelines on toxicity grading scales for healthy adult volunteers enrolled in preventive vaccine clinical trials.[8]	The study employs the use of a reactogenicity e-diary to monitor local reactions and systemic events in real time. Study clinicians will closely monitor for severe reactions. In addition, investigators will contact participants and conduct unscheduled visits for any reported severe local reactions or systemic events.
Unknown AEs and laboratory abnormalities with a novel vaccine.	Accumulating safety data from ongoing clinical studies have shown an acceptable safety profile. Known laboratory abnormalities from the clinical studies conducted in the United States and Germany include transient and self-limited reduction in lymphocyte counts.	The study design includes the use of a clinical laboratory subset to monitor participants for laboratory abnormalities. Participants will be observed for 30 minutes after vaccination to assess for immediate AEs.
Potential for COVID-19 disease enhancement.	Disease enhancement has been seen following vaccination with respiratory syncytial virus (RSV), feline coronavirus, and Dengue virus vaccines.	Participants with likely previous or current COVID-19 will be excluded. All participants will be followed for SARS-CoV-2 antigen-specific antibody and SARS-CoV-2 serum neutralizing titers, and COVID-19 illness, including markers of severity.
**Study Procedures**		
Participants will be required to attend healthcare facilities during the global SARS-CoV-2 pandemic.	Without appropriate social distancing and PPE, there is a potential for increased exposure to SARS-CoV-2.	Pfizer will work with sites to ensure an appropriate COVID-19 prevention strategy. Potential COVID-19 illness visits can be conducted via telehealth, without the need for an in-person visit, if required, with the participant performing a self-swab.
Venipuncture will be performed during the study.	There is the risk of bleeding, bruising, hematoma formation, and infection at the venipuncture site.	Only appropriately qualified personnel will obtain the blood draw.

2.3.2. Benefit Assessment

Benefits to individual participants may include:

- Receipt of a potentially efficacious COVID-19 vaccine during a global pandemic
- Access to COVID-19 diagnostic and antibody testing
- Contributing to research to help others in a time of global pandemic

2.3.3. Overall Benefit/Risk Conclusion

Taking into account the measures taken to minimize risk to participants participating in this study, the potential risks identified in association with BNT162 RNA-based COVID-19 vaccine are justified by the anticipated benefits that may be afforded to healthy participants.

3. OBJECTIVES, ESTIMANDS, AND ENDPOINTS

Objectives	Estimands	Endpoints
**Primary Safety:**		
To describe the safety and tolerability profiles of a prophylactic BNT162 vaccine in healthy Japanese adults after 2 doses	In participants receiving at least 1 dose of study intervention, the percentage of participants reporting:<br>• Local reactions for up to 7 days following each dose<br>• Systemic events for up to 7 days following each dose<br>• AEs from Dose 1 to 1 month after Dose 2<br>• SAEs from Dose 1 to 12 months after Dose 2	• Local reactions (pain at the injection site, redness, and swelling)<br>• Systemic events (fever, fatigue, headache, chills, vomiting, diarrhea, new or worsened muscle pain, and new or worsened joint pain)<br>• AEs<br>• SAEs
	In addition, in a clinical laboratory subset, the percentage of participants with:<br>• Abnormal hematology and chemistry laboratory values 1 and 7 days after Dose 1; and 7 days after Dose 2<br>• Grading shifts in hematology and chemistry laboratory assessments between baseline and 1 and 7 days after Dose 1; and before Dose 2 and 7 days after Dose 2	• Hematology and chemistry laboratory parameters detailed in Section 10.2

090177e1968b9275\Approved\Approved On: 18-Mar-2021 07:58 (GMT)

Objectives	Estimands	Endpoints
**Primary Immunogenicity:**		
To describe the immune responses elicited by a prophylactic BNT162 vaccine in healthy Japanese adults	In participants complying with the key protocol criteria (evaluable participants):<br>• GMTs 1 month after Dose 2<br>• GMFR from before vaccination to 1 month after Dose 2	• SARS-CoV-2 serum neutralizing titers
**Secondary Immunogenicity:**		
To describe the immune responses elicited by a prophylactic BNT162 vaccine in healthy Japanese adults	In the evaluable participants, at baseline, 21 days after Dose 1; 7 and 14 days and 6 and 12 months after Dose 2:<br>• GMTs at each time point<br>• GMFR from before vaccination to each subsequent time point after vaccination	• SARS-CoV-2 serum neutralizing titers
To describe the immune responses elicited by a prophylactic BNT162 vaccine in participants with/without confirmed COVID-19 before Dose 2	In the evaluable participants, at baseline, 21 days after Dose 1; 7 and 14 days and 1, 6, and 12 months after Dose 2:<br>• GMTs at each time point<br>• GMFR from before vaccination to each subsequent time point after vaccination	• SARS-CoV-2 serum neutralizing titers
**Exploratory Immunogenicity:**		
To describe the immune responses elicited by prophylactic BNT162 vaccine in participants with/without confirmed COVID-19 during the study	In the evaluable participants, at baseline, 21 days after Dose 1; 7 and 14 days and 1, 6, and 12 months after Dose 2:<br>• GMTs at each time point<br>• GMFR from before vaccination to each subsequent time point after vaccination	• SARS-CoV-2 serum neutralizing titers
To evaluate the immune response (non-S) to SARS-CoV-2 in participants with/without confirmed COVID-19 during the study		• SARS-CoV-2 N-binding antibody

090177e1968b9275\Approved\Approved On: 18-Mar-2021 07:58 (GMT)

090177e1968b9275\Approved\Approved On: 18-Mar-2021 07:58 (GMT)

4. STUDY DESIGN

4.1. Overall Design

This is a Phase 1/2, randomized, placebo-controlled, and observer-blind study in healthy Japanese adults.

The study will evaluate the safety, tolerability, and immunogenicity of the SARS-CoV-2 RNA vaccine candidate against COVID-19 (BNT162b2), which is the same as the vaccine candidate selected for Phase 2/3 evaluation in the C4591001 study:

- As 2 doses, separated by 21 days
- At a 30-µg dose level
- In adults 20 to 85 years of age

Local reactions (redness, swelling, and pain at the injection site), systemic events (fever, vomiting, diarrhea, headache, fatigue, chills, new or worsened muscle pain, and new or worsened joint pain), and use of antipyretic medication will be prompted for and collected by all participants in an e-diary from Day 1 through Day 7 after each administration of study intervention. AEs will be collected from the time the participant provides informed consent through 1 month after Dose 2, and SAEs will be collected from the time of informed consent through 12 months after Dose 2.

One hundred sixty participants will be randomly assigned in a 3:1 ratio to study intervention (candidate vaccine: 120, placebo: 40), such that approximately 144 evaluable participants (candidate vaccine: 108, placebo: 36) will be evaluated.

As this study is the first study conducted in Japan, clinical laboratory tests will be performed in the first 24 participants (12 participants 20 to 64 years of age and 12 participants 65 to 85 years of age) (clinical laboratory subset). After randomization of all participants in the clinical laboratory subset is completed, the standard participants will be enrolled.

Blood will be collected prior to Dose 1, prior to Dose 2, and 7 and 14 days and 1, 6, and 12 months after Dose 2 to assess immunogenicity.

The study is observer-blinded, as the physical appearance of the investigational vaccine candidates and the placebo may differ. The participant, investigator, study coordinator, and other site staff will be blinded. At the study site, only the dispenser(s)/administrator(s) are unblinded.

To facilitate rapid review of data in real time, sponsor staff will be unblinded to vaccine allocation for the participants.

Participants are expected to participate for up to a maximum of approximately 14 months.

From protocol amendment 3, this study is transitioned from a clinical trial to a postmarketing study according to the Japanese regulation, because BNT162b2 was approved by the MHLW on 14 February 2021.

All participants will be informed of their original study intervention allocation (BNT162b2 or placebo) by the site staff. All participants who originally received placebo will be offered the opportunity to receive BNT162b2 at their earliest convenience as part of the study (administration of BNT162b2 before the 6-month follow-up visit [Visit 8 for the clinical laboratory subset or Visit 6 for the standard participants] is allowed). If they want to receive BNT162b2, they will move to the SoA in Section 1.3.3 for their remaining visits. Participants who originally received BNT162b2 and placebo recipients who decline BNT162b2 vaccination will continue in the study as originally planned.

4.2. Scientific Rationale for Study Design

Additional surveillance for COVID-19 will be conducted as part of the study, given the potential risk of disease enhancement. If a participant experiences symptoms, as detailed in Section 8.13, a COVID-19 illness and subsequent convalescent visit will occur. As part of these visits, local test results of COVID-19 and local diagnosis of COVID-19 as available will be confirmed.

Human reproductive safety data are not available for BNT162 RNA-based COVID-19 vaccines, but there is no suspicion of human teratogenicity based on the intended mechanism of action of the compound. Therefore, the use of a highly effective method of contraception is required (see Appendix 4).

4.3. Justification for Dose

The 2 vaccine candidates (BNT162b1 and BNT162b2) at several dose levels were studied in Phase 1 evaluation in the C4591001 study based on the preliminary experience in the C4591001 study and the BioNTech study (BNT162-01) conducted in Germany. The vaccine candidate selected for Phase 2/3 evaluation in the C4591001 study is BNT162b2 at a dose of 30 µg.

4.4. End of Study Definition

The end of the study is defined as the date of the last visit of the last participant in the study.

5. STUDY POPULATION

This study can fulfill its objectives only if appropriate participants are enrolled. The following eligibility criteria are designed to select participants for whom participation in the study is considered appropriate. All relevant medical and nonmedical conditions should be taken into consideration when deciding whether a particular participant is suitable for this protocol.

Prospective approval of protocol deviations to recruitment and enrollment criteria, also known as protocol waivers or exemptions, is not permitted.

090177e1968b9275\Approved\Approved On: 18-Mar-2021 07:58 (GMT)

5.1. Inclusion Criteria

Participants are eligible to be included in the study only if all of the following criteria apply:

Age and Sex:

1. Japanese male or female participants between the ages of 20 and 85 years, inclusive, at randomization.

    - Refer to Appendix 4 for reproductive criteria for male (Section 10.4.1) and female (Section 10.4.2) participants.

Type of Participant and Disease Characteristics:

2. Participants who are willing and able to comply with all scheduled visits, vaccination plan, laboratory tests, lifestyle considerations, and other study procedures.

3. Healthy participants who are determined by medical history, physical examination, and clinical judgment of the investigator to be eligible for inclusion in the study.

    Note: Healthy participants with preexisting stable disease, defined as disease not requiring significant change in therapy or hospitalization for worsening disease during the 6 weeks before enrollment, can be included.

Informed Consent:

4. Capable of giving signed informed consent as described in Appendix 1, which includes compliance with the requirements and restrictions listed in the ICD and in this protocol.

5.2. Exclusion Criteria

Participants are excluded from the study if any of the following criteria apply:

Medical Conditions:

1. Other medical or psychiatric condition including recent (within the past year) or active suicidal ideation/behavior or laboratory abnormality that may increase the risk of study participation or, in the investigator's judgment, make the participant inappropriate for the study.

2. Known infection with human immunodeficiency virus (HIV), hepatitis C virus (HCV), or hepatitis B virus (HBV).

3. History of severe adverse reaction associated with a vaccine and/or severe allergic reaction (eg, anaphylaxis) to any component of the study intervention(s).

4. Receipt of medications intended to prevent COVID-19.

090177e1968b9275\Approved\Approved On: 18-Mar-2021 07:58 (GMT)

5. Previous confirmed diagnosis of COVID-19 (refer to Section 8.15 for handling participants who have a positive SARS-CoV-2 NAAT result prior to the Vaccination 2 visit).

6. Immunocompromised individuals with known or suspected immunodeficiency, as determined by history and/or laboratory/physical examination.

7. Bleeding diathesis or condition associated with prolonged bleeding that would, in the opinion of the investigator, contraindicate intramuscular injection.

8. Women who are pregnant or breastfeeding.

Prior/Concomitant Therapy:

9. Previous vaccination with any coronavirus vaccine.

10. Individuals who receive treatment with immunosuppressive therapy, including cytotoxic agents or systemic corticosteroids, eg, for cancer or an autoimmune disease, or planned receipt throughout the study. If systemic corticosteroids have been administered short term (<14 days) for treatment of an acute illness, participants should not be enrolled into the study until corticosteroid therapy has been discontinued for at least 28 days before study intervention administration. Inhaled/nebulized, intra-articular, intrabursal, or topical (skin or eyes) corticosteroids are permitted.

11. Receipt of blood/plasma products or immunoglobulin, from 60 days before study intervention administration or planned receipt throughout the study.

Prior/Concurrent Clinical Study Experience:

12. Participation in other studies involving study intervention within 28 days prior to study entry and/or during study participation.

13. Previous participation in other studies involving study intervention containing lipid nanoparticles.

Diagnostic Assessments:

14. **Clinical laboratory subset only:** Any screening hematology and/or blood chemistry laboratory value that meets the definition of a ≥ Grade 1 abnormality.

    Note: With the exception of bilirubin, participants with any stable Grade 1 abnormalities (according to the toxicity grading scale) may be considered eligible at the discretion of the investigator. (Note: A "stable" Grade 1 laboratory abnormality is defined as a report of Grade 1 on an initial blood sample that remains ≤ Grade 1 upon repeat testing on a second sample from the same participant.)

090177e1968b9275\Approved\Approved On: 18-Mar-2021 07:58 (GMT)

Other Exclusions:

15. Investigator site staff or Pfizer employees directly involved in the conduct of the study, site staff otherwise supervised by the investigator, and their respective family members.

5.3. Lifestyle Considerations

5.3.1. Contraception

The investigator or his or her designee, in consultation with the participant, will confirm that the participant has selected an appropriate method of contraception for the individual participant and his or her partner(s) from the permitted list of contraception methods (see Appendix 4, Section 10.4.4) and will confirm that the participant has been instructed in its consistent and correct use. At time points indicated in the SoA, the investigator or designee will inform the participant of the need to use highly effective contraception consistently and correctly and document the conversation and the participant's affirmation in the participant's chart (participants need to affirm their consistent and correct use of at least 1 of the selected methods of contraception). In addition, the investigator or designee will instruct the participant to call immediately if the selected contraception method is discontinued or if pregnancy is known or suspected in the participant or partner.

5.4. Screen Failures

Screen failures are defined as participants who consent to participate in the clinical study but are not subsequently randomly assigned to study intervention. A minimal set of screen failure information is required to ensure transparent reporting of screen failure participants to meet the CONSORT publishing requirements and to respond to queries from regulatory authorities. Minimal information includes demography, screen failure details, eligibility criteria, and any SAEs.

Individuals who do not meet the criteria for participation in this study (screen failure) may be rescreened under a different participant number.

5.5. Criteria for Temporarily Delaying Enrollment/Randomization/Study Intervention Administration

The following conditions are temporary or self-limiting and a participant may be enrolled/randomized/vaccinated once the condition(s) has/have resolved and no other exclusion criteria are met.

1. Current febrile illness (axillary temperature ≥37.5°C) or other acute illness within 48 hours before study intervention administration. This includes current symptoms that could represent a potential COVID-19 illness:

    - New or increased cough;

    - New or increased shortness of breath;

090177e1968b9275\Approved\Approved On: 18-Mar-2021 07:58 (GMT)

- Chills;
- New or increased muscle pain;
- New loss of taste/smell;
- Sore throat;
- Diarrhea;
- Vomiting.

2. Receipt of any seasonal or pandemic influenza vaccine within 14 days, or any other nonstudy vaccine within 28 days, before study intervention administration.

3. Anticipated receipt of any seasonal or pandemic influenza vaccine within 14 days, or any other nonstudy vaccine within 28 days, after study intervention administration.

4. Receipt of short-term (<14 days) systemic corticosteroids. Study intervention administration should be delayed until systemic corticosteroid use has been discontinued for at least 28 days. Inhaled/nebulized, intra-articular, intrabursal, or topical (skin or eyes) corticosteroids are permitted.

6. STUDY INTERVENTION

Study intervention is defined as any investigational intervention(s), marketed product(s), placebo, medical device(s), or study procedure(s) intended to be administered to a study participant according to the study protocol.

The study will evaluate a 2-dose (separated by 21 days) schedule of 30 μg of the investigational RNA vaccine candidate for active immunization against COVID-19, which is the same as that selected for Phase 2/3 evaluation in the C4591001 study. The vaccine candidate or placebo (normal saline) will be administered to a study participant.

6.1. Study Intervention(s) Administered

**Intervention Name**	**BNT162b2 (BNT162 RNA-LNP Vaccine Utilizing modRNA)**	**Saline Placebo**
**Type**	Vaccine	Placebo
**Dose Formulation**	modRNA	Normal saline (0.9% sodium chloride solution for injection)
**Unit Dose Strength**	250 μg/0.5 mL concentrate for solution	N/A
**Dosage Level**	30-μg	N/A
**Route of Administration**	Intramuscular injection	Intramuscular injection
**Use**	Experimental	Placebo
**IMP or NIMP**	IMP	IMP
**Sourcing**	Provided centrally by the sponsor	Provided centrally by the sponsor

090177e1968b9275\Approved\Approved On: 18-Mar-2021 07:58 (GMT)

Intervention Name	BNT162b2 (BNT162 RNA-LNP Vaccine Utilizing modRNA)	Saline Placebo
**Packaging and Labeling**	Study intervention will be provided in a glass vial as open-label supply. Each vial will be labeled as required per country requirement	Study intervention will be provided in a glass or plastic vial as open-label supply. Each vial will be labeled as required per country requirement

6.1.1. Administration

Participants will receive 1 dose of study intervention as randomized at each vaccination visit (Visits 1 and 4 for the clinical laboratory subset, Visits 1 and 2 for the standard participants) in accordance with the study's SoA. Participants who originally received placebo and accept the offer to receive BNT162b2 at defined points as part of the study, will receive 1 dose of BNT162b2 at each additional vaccination visit (Visits 101 and 102) in accordance with the study's additional SoA (Section 1.3.3). Full details are described in the IP manual.

Study intervention should be administered intramuscularly into the deltoid muscle, preferably of the nondominant arm, by an **unblinded** administrator.

Standard vaccination practices must be observed and vaccine must not be injected into blood vessels. Appropriate medication and other supportive measures for management of an acute hypersensitivity reaction should be available in accordance with local guidelines for standard immunization practices.

Administration of study interventions should be performed by an appropriately qualified, GCP-trained, and vaccine-experienced member of the study staff (eg, physician or nurse) as allowed by local, state, and institutional guidance.

Study intervention administration details will be recorded on the CRF.

6.2. Preparation/Handling/Storage/Accountability

1. The investigator or designee must confirm appropriate temperature conditions have been maintained during transit for all study interventions received and any discrepancies are reported and resolved before use of the study intervention.

2. Only participants enrolled in the study may receive study intervention and only authorized site staff may supply or administer study intervention. All study interventions must be stored in a secure, environmentally controlled, and monitored (manual or automated recording) area in accordance with the labeled storage conditions with access limited to the investigator and authorized site staff. At a minimum, daily minimum and maximum temperatures for all site storage locations must be documented and available upon request. Data for nonworking days must indicate the minimum and maximum temperatures since previously documented for all site storage locations upon return to business.

090177e1968b9275\Approved\Approved On: 18-Mar-2021 07:58 (GMT)

3. Any excursions from the study intervention label storage conditions should be reported to Pfizer upon discovery along with any actions taken. The site should actively pursue options for returning the study intervention to the storage conditions described in the labeling, as soon as possible. Once an excursion is identified, the study intervention must be quarantined and not used until Pfizer provides permission to use the study intervention. Specific details regarding the definition of an excursion and information the site should report for each excursion will be provided to the site in the IP manual.

4. Any storage conditions stated in the SRSD will be superseded by the storage conditions stated on the label.

5. Study interventions should be stored in their original containers.

6. See the IP manual for storage conditions of the study intervention.

7. The investigator, institution, or the head of the medical institution (where applicable) is responsible for study intervention accountability, reconciliation, and record maintenance (ie, receipt, reconciliation, and final disposition records), such as the IPAL or sponsor-approved equivalent. All study interventions will be accounted for using a study intervention accountability form/record.

8. Further guidance and information for the final disposition of unused study interventions are provided in the IP manual. All destruction must be adequately documented. If destruction is authorized to take place at the investigator site, the investigator must ensure that the materials are destroyed in compliance with applicable environmental regulations, institutional policy, and any special instructions provided by Pfizer.

Upon identification of a product complaint, notify the sponsor within 1 business day of discovery as described in the IP manual.

6.2.1. Preparation and Dispensing

See the IP manual for instructions on how to prepare the study intervention for administration. Study intervention should be prepared and dispensed by an appropriately qualified and experienced member of the study staff (eg, physician, nurse, physician's assistant, nurse practitioner, pharmacy assistant/technician, or pharmacist) as allowed by local, state, and institutional guidance. A second staff member will verify the dispensing.

Study intervention and placebo will be prepared by qualified unblinded site personnel according to the IP manual. The study intervention will be administered in such a way to ensure the participants remain blinded.

6.3. Measures to Minimize Bias: Randomization and Blinding

6.3.1. Allocation to Study Intervention

Allocation (randomization) of participants to vaccine groups will proceed through the use of an IRT system (IWR). The site personnel (study coordinator or specified designee) will be

090177e1968b9275\Approved\Approved On: 18-Mar-2021 07:58 (GMT)

required to enter or select information including but not limited to the user's ID and password, the protocol number, and the participant number. The site personnel will then be provided with a vaccine assignment and randomization number. The IRT system will provide a confirmation report containing the participant number, randomization number, and study intervention allocation assigned. The confirmation report must be stored in the site's files.

The study-specific IRT reference manual and IP manual will provide the contact information and further details on the use of the IRT system.

6.3.2. Blinding of Site Personnel

In this observer-blinded study, the study staff receiving, storing, dispensing, preparing, and administering the study interventions will be unblinded. All other study and site personnel, including the investigator, investigator staff, and participants, will be blinded to study intervention assignments. In particular, the individuals who evaluate participant safety will be blinded. Because the BNT162 RNA-based COVID-19 vaccine candidates and placebo are different in physical appearance, the study intervention syringes will be administered in a manner that prevents the study participants from identifying the study intervention type based on its appearance.

The responsibility of the unblinded dispenser and administrator must be assigned to an individual or individuals who will not participate in the evaluation of any study participants. Contact between the unblinded dispenser/unblinded administrator and study participants should be kept to a minimum. The remaining site personnel must not know study intervention assignments.

Site staff will be unblinded to all of the individual participants' original study intervention allocations after IRB approval of protocol amendment 3 to inform all participants of the original study intervention allocation (BNT162b2 or placebo) and allow administration of BNT162b2 to participants who originally received placebo.

6.3.3. Blinding of the Sponsor

To facilitate rapid review of data in real time, the majority of sponsor staff will be unblinded to study intervention allocation for all participants. All laboratory testing personnel performing serology assays will remain blinded to study intervention assigned/received throughout the study.

Those study team members who are involved in ensuring that protocol requirements for study intervention preparation, handling, allocation, and administration are fulfilled at the site will be unblinded for the duration of the study (eg, unblinded study manager, unblinded CRA). CRAs and study managers not involved in ensuring that protocol requirements for study intervention preparation, handling, allocation, and administration are fulfilled at the site will remain blinded to study intervention assigned/received throughout the study.

090177e1968b9275\Approved\Approved On: 18-Mar-2021 07:58 (GMT)

At the time when the original study intervention allocation for all participants will be unblinded by the site staff, all members of the study team including CRAs will become unblinded to the participant's original study intervention allocation.

6.3.4. Breaking the Blind

The IRT will be programmed with blind-breaking instructions. In case of an emergency, the investigator has the sole responsibility for determining if unblinding of a participant's study intervention assignment is warranted. Participant safety must always be the first consideration in making such a determination. If the investigator decides that unblinding is warranted, the investigator should make every effort to contact the sponsor prior to unblinding a participant's vaccine assignment unless this could delay further management of the participant. If a participant's vaccine assignment is unblinded, the sponsor must be notified within 24 hours after breaking the blind. The date and reason that the blind was broken must be recorded in the source documentation and CRF.

The study-specific IRT reference manual and IP manual will provide the contact information and further details on the use of the IRT system.

Instructions on how to unblind participants ahead of administration of BNT162b2 to placebo recipients will be provided separately. This unblinding will NOT be performed in the IRT.

6.4. Study Intervention Compliance

When participants are dosed at the site, they will receive study intervention directly from the investigator or designee, under medical supervision. The date and time of each dose administered in the clinic will be recorded in the source documents and recorded in the CRF. The dose of study intervention and study participant identification will be confirmed at the time of dosing by a member of the study site staff other than the person administering the study intervention.

6.5. Concomitant Therapy

The following concomitant medications and vaccinations will be recorded in the CRF:

- All vaccinations received from 28 days prior to study enrollment until the 6-month follow-up visit (Visit 8 for the clinical laboratory subset, Visit 6 for the standard participants).

6.5.1. Prohibited During the Study

Receipt of the following vaccines and medications during the time periods listed below may exclude a participant from the per-protocol analysis from that point onwards, and may require vaccinations to be discontinued in that participant; however, it is anticipated that the participant would not be withdrawn from the study (see Section 7). Medications should not be withheld if required for a participant's medical care.

090177e1968b9275\Approved\Approved On: 18-Mar-2021 07:58 (GMT)

Unless considered medically necessary, no vaccines other than study intervention should be administered within 28 days before and 28 days after each study vaccination. One exception to this is that seasonal and pandemic influenza vaccine can be given at least 14 days after, or at least 14 days prior to, the administration of study intervention.

Receipt of chronic systemic treatment with known immunosuppressant medications, or radiotherapy, within 60 days before enrollment through conclusion of the study.

Receipt of systemic corticosteroids (≥20 mg/day of prednisone or equivalent) for ≥14 days is prohibited from 28 days prior to enrollment to Visit 8 for the clinical laboratory subset and Visit 6 for the standard participants.

Receipt of blood/plasma products or immunoglobulins within 60 days before enrollment through conclusion of the study.

Receipt of any other (nonstudy) coronavirus vaccine at any time prior to or during study participation is prohibited.

Prophylactic antipyretics and other pain medication to prevent symptoms associated with study intervention administration are not permitted. However, if a participant is taking a medication for another condition, even if it may have antipyretic or pain-relieving properties, it should not be withheld prior to study vaccination.

6.5.2. Permitted During the Study

The use of antipyretics and other pain medication to treat symptoms associated with study intervention administration or ongoing conditions is permitted.

Medication other than that described as prohibited in Section 6.5.1 required for treatment of preexisting stable conditions or acute illness is permitted.

Inhaled, topical, or localized injections of corticosteroids (eg, intra-articular or intrabursal administration) are permitted.

Hormonal contraceptives that meet the requirements of this study are allowed to be used in participants who are WOCBP (see Appendix 4).

6.6. Dose Modification

Not applicable.

6.7. Intervention After the End of the Study

No intervention will be provided to study participants at the end of the study. Participants who were originally assigned to placebo will be offered the opportunity to receive BNT162b2 as part of the study.

090177e1968b9275\Approved\Approved On: 18-Mar-2021 07:58 (GMT)

7. DISCONTINUATION OF STUDY INTERVENTION AND PARTICIPANT DISCONTINUATION/WITHDRAWAL

7.1. Discontinuation of Study Intervention

In rare instances, it may be necessary for a participant to permanently discontinue study intervention (definitive discontinuation). Reasons for definitive discontinuation of study intervention may include the following: AEs; Grade 4 local reactions (Section 8.2.2.2), Grade 4 systemic events (Section 8.2.2.3), or fever >40.0°C (Section 8.2.2.4) that is confirmed and determined to be related to the administration of the study intervention; participant request; investigator request; pregnancy; protocol deviation (including no longer meeting all the inclusion criteria, or meeting 1 or more exclusion criteria).

Note that discontinuation of study intervention does not represent withdrawal from the study. Per the study estimands, if study intervention is definitively discontinued, the participant will remain in the study to be evaluated for safety and immunogenicity and for COVID-19 disease surveillance. See the SoA for data to be collected at the time of discontinuation of study intervention and follow-up for any further evaluations that need to be completed.

In the event of discontinuation of study intervention, it must be documented on the appropriate CRF/in the medical records whether the participant is discontinuing further receipt of study intervention or also from study procedures, postvaccination study follow-up, and/or future collection of additional information.

7.2. Participant Discontinuation/Withdrawal From the Study

A participant may withdraw from the study at any time at his/her own request. Reasons for discontinuation from the study may include the following:

- Refused further follow-up;
- Lost to follow-up;
- Death;
- Study terminated by sponsor;
- AEs;
- Participant request;
- Investigator request;
- Protocol deviation.

If a participant does not return for a scheduled visit, every effort should be made to contact the participant. All attempts to contact the participant and information received during

090177e1968b9275\Approved\Approved On: 18-Mar-2021 07:58 (GMT)

contact attempts must be documented in the participant's source document. In any circumstance, every effort should be made to document participant outcome, if possible.

The investigator or his or her designee should capture the reason for withdrawal in the CRF for all participants.

If a participant withdraws from the study, he/she may request destruction of any remaining samples taken and not tested, and the investigator must document any such requests in the site study records and notify the sponsor accordingly.

If the participant withdraws from the study and also withdraws consent (see Section 7.2.1) for disclosure of future information, no further evaluations should be performed and no additional data should be collected. The sponsor may retain and continue to use any data collected before such withdrawal of consent.

Lack of completion of all or any of the withdrawal/early termination procedures will not be viewed as protocol deviations so long as the participant's safety was preserved.

7.2.1. Withdrawal of Consent

Participants who request to discontinue receipt of study intervention will remain in the study and must continue to be followed for protocol-specified follow-up procedures. The only exception to this is when a participant specifically withdraws consent for any further contact with him or her or persons previously authorized by the participant to provide this information. Participants should notify the investigator in writing of the decision to withdraw consent from future follow-up, whenever possible. The withdrawal of consent should be explained in detail in the medical records by the investigator, as to whether the withdrawal is only from further receipt of study intervention or also from study procedures and/or postvaccination study follow-up, and entered on the appropriate CRF page. In the event that vital status (whether the participant is alive or dead) is being measured, publicly available information should be used to determine vital status only as appropriately directed in accordance with local law.

7.3. Lost to Follow-up

A participant will be considered lost to follow-up if he or she repeatedly fails to return for scheduled visits and is unable to be contacted by the study site.

The following actions must be taken if a participant fails to attend a required study visit:

- The site must attempt to contact the participant and reschedule the missed visit as soon as possible and counsel the participant on the importance of maintaining the assigned visit schedule and ascertain whether or not the participant wishes to and/or should continue in the study;

- Before a participant is deemed lost to follow-up, the investigator or designee must make every effort to regain contact with the participant (where possible, 3 telephone calls and,

090177e1968b9275\Approved\Approved On: 18-Mar-2021 07:58 (GMT)

if necessary, a certified letter to the participant's last known mailing address or local equivalent methods). These contact attempts should be documented in the participant's medical record;

- Should the participant continue to be unreachable, he/she will be considered to have withdrawn from the study.

8. STUDY ASSESSMENTS AND PROCEDURES

The investigator (or an appropriate delegate at the investigator site) must obtain a signed and dated ICD before performing any study-specific procedures.

The full date of birth will be collected to critically evaluate the immune response and safety profile by age.

Study procedures and their timing are summarized in the SoA. Protocol waivers or exemptions are not allowed.

Safety issues should be discussed with the sponsor immediately upon occurrence or awareness to determine whether the participant should continue or discontinue study intervention.

Adherence to the study design requirements, including those specified in the SoA, is essential and required for study conduct.

All screening evaluations must be completed and reviewed to confirm that potential participants meet all eligibility criteria. The investigator will maintain a screening log to record details of all participants screened and to confirm eligibility or record reasons for screening failure, as applicable.

Every effort should be made to ensure that protocol-required tests and procedures are completed as described. However, it is anticipated that from time to time there may be circumstances outside the control of the investigator that may make it unfeasible to perform the test. In these cases, the investigator must take all steps necessary to ensure the safety and well-being of the participant. When a protocol-required test cannot be performed, the investigator will document the reason for the missed test and any corrective and preventive actions that he or she has taken to ensure that required processes are adhered to as soon as possible. The study team must be informed of these incidents in a timely manner.

For samples being collected and shipped, detailed collection, processing, storage, and shipment instructions and contact information will be provided to the investigator site prior to initiation of the study.

The total blood sampling volume for individual participants in this study is approximately 225 mL for the clinical laboratory subset and 175 mL for the standard participants. Other additional blood samples may be taken for safety assessments at times specified by Pfizer,

090177e1968b9275\Approved\Approved On: 18-Mar-2021 07:58 (GMT)

provided the total volume taken during the study does not exceed 550 mL during any period of 60 consecutive days.

8.1. Efficacy and/or Immunogenicity Assessments

Serum samples will be obtained for immunogenicity testing at the visits specified in the SoA. The following assays will be performed:

- SARS-CoV-2 serum neutralization assay
- Roche Elecsys Anti-SARS-CoV-2, an electrochemiluminescence immunoassay (ECLIA) for the presence or absence of SARS-CoV-2 N-binding antibodies

Note that all immunogenicity analyses will be based upon samples analyzed at the central laboratory. Additional exploratory assays to measure immune responses may be performed, with analyses described in the SAP.

8.1.1. Biological Samples

Blood samples for immunogenicity and nasal swab samples to be collected before Dose 1 and before Dose 2 will be used only for scientific research. Each sample will be labeled with a code so that the laboratory personnel testing the samples will not know the participant's identity. Samples for immunogenicity that remain after performing assays outlined in the protocol may be stored by Pfizer. Unless a time limitation is required by local regulations or ethical requirements, the samples will be stored for up to 15 years after the end of the study and then destroyed. If allowed by the ICD, stored samples may be used for additional testing to better understand the immune responses to the vaccine(s) under study in this protocol, to inform the development of other products, and/or for vaccine-related assay work supporting vaccine programs. No testing of the participant's DNA will be performed.

The participant may request that his or her samples, if still identifiable, be destroyed at any time; however, any data already collected from those samples will still be used for this research. The biological samples may be shared with other researchers as long as confidentiality is maintained and no testing of the participant's DNA is performed.

8.2. Safety Assessments

Planned time points for all safety assessments are provided in the SoA. Unscheduled clinical laboratory measurements may be obtained at any time during the study to assess any perceived safety issues.

A clinical assessment, including medical history, will be performed on all participants at his/her first visit to establish a baseline. Significant medical history and observations from any physical examination, if performed, will be documented in the CRF. Also, a clinical assessment will be performed on participants enrolled in the clinical laboratory subset at Visits 1 through 4.

AEs and SAEs are collected, recorded, and reported as defined in Section 8.3.

090177e1968b9275\Approved\Approved On: 18-Mar-2021 07:58 (GMT)

Acute reactions within the first 30 minutes will be assessed and documented in the AE CRF.

The safety parameters also include reactogenicity e-diary reports of local reactions and systemic events (including fever), and use of antipyretic medication, that occur in the 7 days after administration of the study intervention. These prospectively self-collected occurrences of local reactions and systemic events are graded as described in Section 8.2.2.

Clinical laboratory tests will be performed in the first 24 participants (12 participants 20 to 64 years of age and 12 participants 65 to 85 years of age).

8.2.1. Clinical Safety Laboratory Assessments (Clinical Laboratory Subset Only)

See Appendix 2 for the list of clinical safety laboratory tests to be performed and the SoA for the timing and frequency. All protocol-required laboratory assessments, as defined in Appendix 2, must be conducted in accordance with the laboratory manual and the SoA. Unscheduled clinical laboratory measurements may be obtained at any time during the study to assess any perceived safety issues.

The investigator must review the laboratory report, document this review, and record any clinically relevant changes occurring during the study in the AE section of the CRF. See Appendix 2 for the grading scale for assessment of clinically significant abnormal laboratory findings. Clinically significant abnormal laboratory findings are those that are not associated with the underlying disease, unless judged by the investigator to be more severe than expected for the participant's condition.

All laboratory tests with values considered clinically significantly abnormal during participation in the study or within 28 days after Dose 2 of study intervention should be repeated until the values return to normal or baseline or are no longer considered clinically significant by the investigator or medical monitor.

If such values do not return to normal/baseline within a period of time judged reasonable by the investigator, the etiology should be identified and the sponsor notified.

See Appendix 6 for suggested actions and follow-up assessments in the event of potential drug-induced liver injury.

8.2.2. Electronic Diary

Participants will be required to complete a reactogenicity e-diary through an application (see Section 8.14) installed on a provisioned device or on the participant’s own personal device. All participants will be asked to monitor and record local reactions, systemic events, and antipyretic medication usage for 7 days following administration of the study intervention. All participants who originally received placebo and then received BNT162b2 under protocol amendment 3 and onwards, will not complete a reactogenicity e-diary but will have their local reactions and systemic events collected and reported as AEs in accordance with Section 8.3.2.

090177e1968b9275\Approved\Approved On: 18-Mar-2021 07:58 (GMT)

The reactogenicity e-diary allows recording of these assessments only within a fixed time window, thus providing the accurate representation of the participant's experience at that time. Data on local reactions and systemic events reported in the reactogenicity e-diary will be transferred electronically to a third-party vendor, where they will be available for review by investigators and the Pfizer clinicians at all times via an internet-based portal.

At intervals agreed to by the vendor and Pfizer, these data will be transferred electronically into Pfizer's database for analysis and reporting. These data do not need to be reported by the investigator in the CRF as AEs.

Investigators (or designee) will be required to review the reactogenicity e-diary data online at frequent intervals as part of the ongoing safety review.

The investigator or designee must obtain stop dates from the participant for any ongoing local reactions, systemic events, or use of antipyretic medication on the last day that the reactogenicity e-diary was completed. The stop dates should be documented in the source documents and the information entered in the CRF.

8.2.2.1. Grading Scales

The grading scales used in this study to assess local reactions and systemic events as described below are derived from the FDA CBER guidelines on toxicity grading scales for healthy adult volunteers enrolled in preventive vaccine clinical trials.[8]

8.2.2.2. Local Reactions

During the reactogenicity e-diary reporting period, participants will be asked to assess redness, swelling, and pain at the injection site and to record the symptoms in the reactogenicity e-diary. If a local reaction persists beyond the end of the reactogenicity e-diary period following vaccination, the participant will be requested to report that information. The investigator will enter this additional information in the CRF.

Redness and swelling will be measured and recorded in measuring device units (range: 1 to 21) and then categorized during analysis as absent, mild, moderate, or severe based on the grading scale in Table 1. Measuring device units can be converted to centimeters according to the following formula: 1 measuring device unit = 0.5 cm. Pain at the injection site will be assessed by the participant as absent, mild, moderate, or severe according to the grading scale in Table 1.

If a Grade 3 local reaction is reported in the reactogenicity e-diary, a telephone contact should occur to ascertain further details and determine whether a site visit is clinically indicated. Only an investigator or medically qualified person is able to classify a participant's local reaction as Grade 4. If a participant experiences a confirmed Grade 4 local reaction, the investigator must immediately notify the sponsor and, if it is determined to be related to the administration of the study intervention, further vaccinations will be discontinued in that participant.

090177e1968b9275\Approved\Approved On: 18-Mar-2021 07:58 (GMT)

Table 1. Local Reaction Grading Scale

	Mild (Grade 1)	Moderate (Grade 2)	Severe (Grade 3)	Potentially Life Threatening (Grade 4)
**Pain at the injection site**	Does not interfere with activity	Interferes with activity	Prevents daily activity	Emergency room visit or hospitalization for severe pain
**Redness**	2.0 cm to 5.0 cm (5 to 10 measuring device units)	>5.0 cm to 10.0 cm (11 to 20 measuring device units)	>10 cm (≥21 measuring device units)	Necrosis or exfoliative dermatitis
**Swelling**	2.0 cm to 5.0 cm (5 to 10 measuring device units)	>5.0 cm to 10.0 cm (11 to 20 measuring device units)	>10 cm (≥21 measuring device units)	Necrosis

8.2.2.3. Systemic Events

During the reactogenicity e-diary reporting period, participants will be asked to assess vomiting, diarrhea, headache, fatigue, chills, new or worsened muscle pain, and new or worsened joint pain and to record the symptoms in the reactogenicity e-diary. The symptoms will be assessed by the participant as absent, mild, moderate, or severe according to the grading scale in Table 2.

If a Grade 3 systemic event is reported in the reactogenicity e-diary, a telephone contact should occur to ascertain further details and determine whether a site visit is clinically indicated. Only an investigator or medically qualified person is able to classify a participant's systemic event as Grade 4. If a participant experiences a confirmed Grade 4 systemic event, the investigator must immediately notify the sponsor and, if it is determined to be related to the administration of the study intervention, further vaccinations will be discontinued in that participant.

090177e1968b9275\Approved\Approved On: 18-Mar-2021 07:58 (GMT)

Table 2. Systemic Event Grading Scale

	Mild (Grade 1)	Moderate (Grade 2)	Severe (Grade 3)	Potentially Life Threatening (Grade 4)
**Vomiting**	1-2 times in 24 hours	>2 times in 24 hours	Requires IV hydration	Emergency room visit or hospitalization for hypotensive shock
**Diarrhea**	2 to 3 loose stools in 24 hours	4 to 5 loose stools in 24 hours	6 or more loose stools in 24 hours	Emergency room visit or hospitalization for severe diarrhea
**Headache**	Does not interfere with activity	Some interference with activity	Prevents daily routine activity	Emergency room visit or hospitalization for severe headache
**Fatigue/ tiredness**	Does not interfere with activity	Some interference with activity	Prevents daily routine activity	Emergency room visit or hospitalization for severe fatigue
**Chills**	Does not interfere with activity	Some interference with activity	Prevents daily routine activity	Emergency room visit or hospitalization for severe chills
**New or worsened muscle pain**	Does not interfere with activity	Some interference with activity	Prevents daily routine activity	Emergency room visit or hospitalization for severe new or worsened muscle pain
**New or worsened joint pain**	Does not interfere with activity	Some interference with activity	Prevents daily routine activity	Emergency room visit or hospitalization for severe new or worsened joint pain

Abbreviation: IV = intravenous.

8.2.2.4. Fever

In order to record information on fever, a thermometer will be given to participants with instructions on how to measure axillary temperature at home. Temperature will be collected in the reactogenicity e-diary in the evening daily during the reactogenicity e-diary reporting period. It will also be collected at any time during the reactogenicity e-diary data collection periods when fever is suspected. Fever is defined as an axillary temperature of ≥37.5°C. The highest temperature for each day will be recorded in the reactogenicity e-diary. Temperature will be measured and recorded to 1 decimal place and then categorized during analysis according to the scale shown in Table 3.

If a fever of ≥39.0°C is reported in the reactogenicity e-diary, a telephone contact should occur to ascertain further details and determine whether a site visit is clinically indicated. Only an investigator or medically qualified person is able to confirm a participant's fever as >40.0°C. If a participant experiences a confirmed fever >40.0°C, the investigator must immediately notify the sponsor and, if it is determined to be related to the administration of the study intervention, further vaccinations will be discontinued in that participant.

090177e1968b9275\Approved\Approved On: 18-Mar-2021 07:58 (GMT)

Table 3. Scale for Fever

≥37.5-38.4°C
>38.4-38.9°C
>38.9-40.0°C
>40.0°C

Note: Fever is defined as axillary temperature ≥37.5°C.

8.2.2.5. Antipyretic Medication

The use of antipyretic medication to treat symptoms associated with study intervention administration will be recorded in the reactogenicity e-diary daily during the reporting period (Day 1 to Day 7).

8.2.3. Surveillance of Events That Could Represent Enhanced COVID-19 Disease

As this is a sponsor open-label study, the sponsor will conduct unblinded reviews of the data during the course of the study, including for the purpose of safety assessment.

Participants will be surveilled for potential COVID-19 illness from Visit 1 onwards (see Section 8.13).

8.2.4. Pregnancy Testing

Pregnancy tests may be urine or serum tests, but must have a sensitivity of at least 25 mIU/mL. Pregnancy tests will be performed in WOCBP at the times listed in the SoA, immediately before the administration of each vaccine dose. A negative pregnancy test result will be required prior to the participant's receiving the study intervention. Pregnancy tests may also be repeated if requested by IRBs/ECs or if required by local regulations. In the case of a positive confirmed pregnancy, the participant will be withdrawn from administration of study intervention but may remain in the study.

8.3. Adverse Events and Serious Adverse Events

The definitions of an AE and an SAE can be found in Appendix 3.

AEs will be reported by the participant (or, when appropriate, by a caregiver, surrogate, or the participant's legally authorized representative).

The investigator and any qualified designees are responsible for detecting, documenting, and recording events that meet the definition of an AE or SAE and remain responsible to pursue and obtain adequate information both to determine the outcome and to assess whether the event meets the criteria for classification as an SAE or caused the participant to discontinue the study intervention (see Section 7.1).

Each participant will be questioned about the occurrence of AEs in a nonleading manner.

090177e1968b9275\Approved\Approved On: 18-Mar-2021 07:58 (GMT)

In addition, the investigator may be requested by Pfizer Safety to obtain specific follow-up information in an expedited fashion.

8.3.1. Time Period and Frequency for Collecting AE and SAE Information

The time period for actively eliciting and collecting AEs ("active collection period") for each participant begins from the time the participant provides informed consent, which is obtained before the participant's participation in the study (ie, before undergoing any study-related procedure and/or receiving study intervention), through and including Visit 7 for the clinical laboratory subset and Visit 5 for the standard participants. In addition, any AEs occurring up to 48 hours after each subsequent blood draw must be recorded on the CRF.

SAEs will be collected from the time the participant provides informed consent to approximately 12 months after Dose 2 of study intervention (Visit 9 for the clinical laboratory subset, Visit 7 for the standard participants).

Confirmed COVID-19 diagnosis (SARS-CoV-2 positive test) will be collected as an AESI from the time the participant provides informed consent until study completion (Visit 9 for the clinical laboratory subset, Visit 7 for standard participants) (see Section 8.3.8).

Additionally, for those participants who originally received placebo but go on to receive BNT162b2 at Vaccinations 3 and 4, AEs will be collected from the time the participant provides informed consent (for receipt of Vaccinations 3 and 4) through and including Visit 103. SAEs and confirmed COVID-19 diagnosis (SARS-CoV-2 positive test) will be collected from the time the participant provides informed consent (for receipt of Vaccinations 3 and 4) to approximately 6 months after Dose 2 of BNT162b2 (Visit 104).

Follow-up by the investigator continues throughout and after the active collection period and until the AE or SAE or its sequelae resolve or stabilize at a level acceptable to the investigator and Pfizer concurs with that assessment.

For participants who are screen failures, the active collection period ends when screen failure status is determined.

If the participant withdraws from the study and also withdraws consent for the collection of future information, the active collection period ends when consent is withdrawn.

If a participant definitively discontinues or temporarily discontinues study intervention because of an AE or SAE, the AE or SAE must be recorded on the CRF and the SAE reported using the Vaccine SAE Reporting Form.

Investigators are not obligated to actively seek AEs or SAEs after the participant has concluded study participation. However, if the investigator learns of any SAE, including a death, at any time after a participant has completed the study, and he/she considers the event to be reasonably related to the study intervention, the investigator must promptly report the SAE to Pfizer using the Vaccine SAE Reporting Form.

090177e1968b9275\Approved\Approved On: 18-Mar-2021 07:58 (GMT)

8.3.1.1. Reporting SAEs to Pfizer Safety

All SAEs occurring in a participant during the active collection period as described in Section 8.3.1 are reported to Pfizer Safety on the Vaccine SAE Reporting Form immediately upon awareness and under no circumstance should this exceed 24 hours, as indicated in Appendix 3. The investigator will submit any updated SAE data to the sponsor within 24 hours of it being available.

8.3.1.2. Recording Nonserious AEs and SAEs on the CRF

All nonserious AEs and SAEs occurring in a participant during the active collection period, which begins after obtaining informed consent as described in Section 8.3.1, will be recorded on the AE section of the CRF.

The investigator is to record on the CRF all directly observed and all spontaneously reported AEs and SAEs reported by the participant.

8.3.2. Method of Detecting AEs and SAEs

The method of recording, evaluating, and assessing causality of AEs and SAEs and the procedures for completing and transmitting SAE reports are provided in Appendix 3.

Care will be taken not to introduce bias when detecting AEs and/or SAEs. Open-ended and nonleading verbal questioning of the participant is the preferred method to inquire about AE occurrences.

8.3.3. Follow-up of AEs and SAEs

After the initial AE/SAE report, the investigator is required to proactively follow each participant at subsequent visits/contacts. For each event, the investigator must pursue and obtain adequate information until resolution, stabilization, the event is otherwise explained, or the participant is lost to follow-up (as defined in Section 7.3).

In general, follow-up information will include a description of the event in sufficient detail to allow for a complete medical assessment of the case and independent determination of possible causality. Any information relevant to the event, such as concomitant medications and illnesses, must be provided. In the case of a participant death, a summary of available autopsy findings must be submitted as soon as possible to Pfizer Safety.

Further information on follow-up procedures is given in Appendix 3.

8.3.4. Regulatory Reporting Requirements for SAEs

Prompt notification by the investigator to the sponsor of an SAE is essential so that legal obligations and ethical responsibilities towards the safety of participants and the safety of a study intervention under clinical investigation are met.

The sponsor has a legal responsibility to notify both the local regulatory authority and other regulatory agencies about the safety of a study intervention under clinical investigation. The

090177e1968b9275\Approved\Approved On: 18-Mar-2021 07:58 (GMT)

sponsor will comply with country-specific regulatory requirements relating to safety reporting to the regulatory authority, IRBs/ECs, and investigators.

Investigator safety reports must be prepared for SUSARs according to local regulatory requirements and sponsor policy and forwarded to investigators as necessary.

An investigator who receives SUSARs or other specific safety information (eg, summary or listing of SAEs) from the sponsor will review and then file it along with the SRSD(s) for the study and will notify the IRB/EC, if appropriate according to local requirements.

8.3.5. Exposure During Pregnancy or Breastfeeding, and Occupational Exposure

Exposure to the study intervention under study during pregnancy or breastfeeding and occupational exposure are reportable to Pfizer Safety within 24 hours of investigator awareness.

8.3.5.1. Exposure During Pregnancy

An EDP occurs if:

- A female participant is found to be pregnant while receiving or after discontinuing study intervention.
- A male participant who is receiving or has discontinued study intervention exposes a female partner prior to or around the time of conception.
- A female is found to be pregnant while being exposed or having been exposed to study intervention due to environmental exposure. Below are examples of environmental exposure during pregnancy:
  - A female family member or healthcare provider reports that she is pregnant after having been exposed to the study intervention by inhalation or skin contact.
  - A male family member or healthcare provider who has been exposed to the study intervention by inhalation or skin contact then exposes his female partner prior to or around the time of conception.

The investigator must report EDP to Pfizer Safety within 24 hours of the investigator's awareness, irrespective of whether an SAE has occurred. The initial information submitted should include the anticipated date of delivery (see below for information related to termination of pregnancy).

- If EDP occurs in a participant or a participant's partner, the investigator must report this information to Pfizer Safety on the Vaccine SAE Reporting Form and an EDP Supplemental Form, regardless of whether an SAE has occurred. Details of the pregnancy will be collected after the start of study intervention and until 12 months after Dose 2 of study intervention and until delivery.

090177e1968b9275\Approved\Approved On: 18-Mar-2021 07:58 (GMT)

- If EDP occurs in the setting of environmental exposure, the investigator must report information to Pfizer Safety using the Vaccine SAE Reporting Form and EDP Supplemental Form. Since the exposure information does not pertain to the participant enrolled in the study, the information is not recorded on a CRF; however, a copy of the completed Vaccine SAE Reporting Form is maintained in the investigator site file.

Follow-up is conducted to obtain general information on the pregnancy and its outcome for all EDP reports with an unknown outcome. The investigator will follow the pregnancy until completion (or until pregnancy termination) and notify Pfizer Safety of the outcome as a follow-up to the initial EDP Supplemental Form. In the case of a live birth, the structural integrity of the neonate can be assessed at the time of birth. In the event of a termination, the reason(s) for termination should be specified and, if clinically possible, the structural integrity of the terminated fetus should be assessed by gross visual inspection (unless preprocedure test findings are conclusive for a congenital anomaly and the findings are reported).

Abnormal pregnancy outcomes are considered SAEs. If the outcome of the pregnancy meets the criteria for an SAE (ie, ectopic pregnancy, spontaneous abortion, intrauterine fetal demise, neonatal death, or congenital anomaly), the investigator should follow the procedures for reporting SAEs. Additional information about pregnancy outcomes that are reported to Pfizer Safety as SAEs follows:

- Spontaneous abortion including miscarriage and missed abortion;
- Neonatal deaths that occur within 1 month of birth should be reported, without regard to causality, as SAEs. In addition, infant deaths after 1 month should be reported as SAEs when the investigator assesses the infant death as related or possibly related to exposure to the study intervention.

Additional information regarding the EDP may be requested by the sponsor. Further follow-up of birth outcomes will be handled on a case-by-case basis (eg, follow-up on preterm infants to identify developmental delays). In the case of paternal exposure, the investigator will provide the participant with the Pregnant Partner Release of Information Form to deliver to his partner. The investigator must document in the source documents that the participant was given the Pregnant Partner Release of Information Form to provide to his partner.

8.3.5.2. Exposure During Breastfeeding

An exposure during breastfeeding occurs if:

- A female participant is found to be breastfeeding while receiving or after discontinuing study intervention.

090177e1968b9275\Approved\Approved On: 18-Mar-2021 07:58 (GMT)

- A female is found to be breastfeeding while being exposed or having been exposed to study intervention (ie, environmental exposure). An example of environmental exposure during breastfeeding is a female family member or healthcare provider who reports that she is breastfeeding after having been exposed to the study intervention by inhalation or skin contact.

The investigator must report exposure during breastfeeding to Pfizer Safety within 24 hours of the investigator's awareness, irrespective of whether an SAE has occurred. The information must be reported using the Vaccine SAE Reporting Form. When exposure during breastfeeding occurs in the setting of environmental exposure, the exposure information does not pertain to the participant enrolled in the study, so the information is not recorded on a CRF. However, a copy of the completed Vaccine SAE Reporting Form is maintained in the investigator site file.

An exposure during breastfeeding report is not created when a Pfizer drug specifically approved for use in breastfeeding women (eg, vitamins) is administered in accord with authorized use. However, if the infant experiences an SAE associated with such a drug, the SAE is reported together with the exposure during breastfeeding.

8.3.5.3. Occupational Exposure

An occupational exposure occurs when a person receives unplanned direct contact with the study intervention, which may or may not lead to the occurrence of an AE. Such persons may include healthcare providers, family members, and other roles that are involved in the trial participant's care.

The investigator must report occupational exposure to Pfizer Safety within 24 hours of the investigator's awareness, regardless of whether there is an associated SAE. The information must be reported using the Vaccine SAE Reporting Form. Since the information does not pertain to a participant enrolled in the study, the information is not recorded on a CRF; however, a copy of the completed Vaccine SAE Reporting Form is maintained in the investigator site file.

8.3.6. Cardiovascular and Death Events

Not applicable.

8.3.7. Disease-Related Events and/or Disease-Related Outcomes Not Qualifying as AEs or SAEs

Not applicable.

090177e1968b9275\Approved\Approved On: 18-Mar-2021 07:58 (GMT)

8.3.8. Adverse Events of Special Interest

This section provides information on AESIs that may be detected during the study:

- Confirmed COVID-19 diagnosis (positive SARS-CoV-2 test)

All AESIs must be reported as an AE or SAE following the procedures described in Sections 8.3.1 through 8.3.4. An AESI is to be recorded as an AE or SAE on the CRF. In addition, an AESI that is also an SAE must be reported using the Vaccine SAE Reporting Form.

8.3.8.1. Lack of Efficacy

Lack of efficacy is reportable to Pfizer Safety only if associated with an SAE.

8.3.9. Medical Device Deficiencies

Not applicable.

8.3.10. Medication Errors

Medication errors may result from the administration or consumption of the study intervention by the wrong participant, or at the wrong time, or at the wrong dosage strength.

Exposures to the study intervention under study may occur in clinical trial settings, such as medication errors.

Safety Event	Recorded on the CRF	Reported on the Vaccine SAE Reporting Form to Pfizer Safety Within 24 Hours of Awareness
Medication errors	All (regardless of whether associated with an AE)	Only if associated with an SAE

Medication errors include:

- Medication errors involving participant exposure to the study intervention;
- Potential medication errors or uses outside of what is foreseen in the protocol that do or do not involve the study participant;
- The administration of expired study intervention;
- The administration of an incorrect study intervention;
- The administration of an incorrect dosage;

090177e1968b9275\Approved\Approved On: 18-Mar-2021 07:58 (GMT)

- The administration of study intervention that has undergone temperature excursion from the specified storage range, unless it is determined by the sponsor that the study intervention under question is acceptable for use.

Such medication errors occurring to a study participant are to be captured on the medication error page of the CRF, which is a specific version of the AE page.

In the event of a medication dosing error, the sponsor should be notified within 24 hours.

Whether or not the medication error is accompanied by an AE, as determined by the investigator, the medication error is recorded on the medication error page of the CRF and, if applicable, any associated AE(s), serious and nonserious, are recorded on the AE page of the CRF.

Medication errors should be reported to Pfizer Safety within 24 hours on a Vaccine SAE Reporting Form **only when associated with an SAE.**

8.4. Treatment of Overdose

For this study, any dose of study intervention greater than 1 dose of study intervention within a 24-hour time period will be considered an overdose.

Pfizer does not recommend specific treatment for an overdose.

In the event of an overdose, the investigator should:

1. Contact the medical monitor within 24 hours.

2. Closely monitor the participant for any AEs/SAEs.

3. Document the quantity of the excess dose as well as the duration of the overdose in the CRF.

4. Overdose is reportable to Safety **only when associated with an SAE.**

Decisions regarding dose interruptions or modifications will be made by the investigator in consultation with the medical monitor based on the clinical evaluation of the participant.

8.5. Pharmacokinetics

Pharmacokinetic parameters are not evaluated in this study.

8.6. Pharmacodynamics

Pharmacodynamic parameters are not evaluated in this study.

8.7. Genetics

Genetics (specified analyses) are not evaluated in this study.

090177e1968b9275\Approved\Approved On: 18-Mar-2021 07:58 (GMT)

8.8. Biomarkers

Biomarkers are not evaluated in this study.

8.9. Immunogenicity Assessments

Immunogenicity assessments are described in Section 8.1.

8.10. Health Economics

Health economics/medical resource utilization and health economics parameters are not evaluated in this study.

8.11. Study Procedures

Unless stated otherwise, all study visits are intended to be conducted in person at the study site. If this is not possible because of local circumstances related to the COVID-19 pandemic, study procedures that do not require in-person participant contact may be performed by telehealth. Telehealth includes the exchange of healthcare information and services via telecommunication technologies (eg, audio, video, and video-conferencing software) remotely, allowing the participant and the investigator to communicate on aspects of clinical care, including medical advice, reminders, education, and safety monitoring. Irrespective of the nature of the contact, all visit procedures are expected to be performed on the same day.

8.11.1. Clinical Laboratory Subset

8.11.1.1. Screening (0 to 28 Days Before Visit 1)

Before enrollment and before any study-related procedures are performed, voluntary, written study-specific informed consent will be obtained from the participant. Each signature on the ICD must be personally dated by the signatory. The investigator or his or her designee will also sign the ICD. A copy of the signed and dated ICD must be given to the participant. The source data must reflect that the informed consent was obtained before participation in the study.

It is anticipated that the procedures below will be conducted in a stepwise manner; however, the visit can occur over more than 1 day.

- Assign a single participant number using the IRT system.
- Obtain the participant’s demography (including date of birth, sex, race, and ethnicity). The full date of birth will be collected to critically evaluate the immune response and safety profile by age.
- Obtain any medical history of clinical significance.
- Perform a clinical assessment. If the clinical assessment indicates that a physical examination is necessary to comprehensively evaluate the participant, perform a physical

090177e1968b9275\Approved\Approved On: 18-Mar-2021 07:58 (GMT)

examination and record any findings in the source documents and, if clinically significant, record on the medical history CRF.

- Measure the participant's height and weight.
- Collect a blood sample (approximately 10 mL) for hematology and chemistry laboratory tests as described in Section 10.2.
- Perform urine pregnancy test on WOCBP as described in Section 8.2.4.
- Discuss contraceptive use as described in Section 10.4.
- Record nonstudy vaccinations as described in Section 6.5.
- Ensure and document that all of the inclusion criteria and none of the exclusion criteria are met.
- Record AEs as described in Section 8.3. AEs that occur prior to dosing should be noted on the Medical History CRF.
- Ask the participant to contact the site staff or investigator immediately if any significant illness or hospitalization occurs.
- Ask the participant to contact the site staff or investigator immediately if he or she experiences any symptoms as detailed in Section 8.13.
- Schedule an appointment for the participant to return for the next study visit.
- Complete the source documents.
- Complete the CRF.

8.11.1.2. Visit 1 – Vaccination 1 (Day 1)

It is anticipated that the procedures below will be conducted in a stepwise manner; ensure that procedures listed prior to administration of the vaccine are conducted prior to vaccination.

- Record AEs as described in Section 8.3.
- Perform a clinical assessment. If the clinical assessment indicates that a physical examination is necessary to comprehensively evaluate the participant, perform a physical examination and record any findings in the source documents and, if clinically significant, record on the AE CRF.
- Perform urine pregnancy test on WOCBP as described in Section 8.2.4.

090177e1968b9275\Approved\Approved On: 18-Mar-2021 07:58 (GMT)

- Discuss contraceptive use as described in Section 10.4.
- Record nonstudy vaccinations as described in Section 6.5.
- Review screening laboratory results (hematology and chemistry tests).
- Ensure and document that all of the inclusion criteria and none of the exclusion criteria are met.
- Measure the participant's body temperature (axillary).
- Ensure that the participant meets none of the temporary delay criteria as described in Section 5.5.
- Obtain the participant's randomization number and study intervention allocation using the IRT system. Only an unblinded site staff member may obtain this information.
- Collect a blood sample (approximately 25 mL) for immunogenicity testing.
- Obtain a nasal (midturbinate) swab (collected by site staff).
- Unblinded site staff member(s) will dispense/administer 1 dose of study intervention into the deltoid muscle of the (preferably) nondominant arm. Please refer to the IP manual for further instruction on this process.
- Blinded site staff must observe the participant for at least 30 minutes after study intervention administration for any acute reactions. Record any acute reactions (including time of onset) in the participant's source documents and on the AE page of the CRF, and on an SAE form as applicable.
- Issue a measuring device to measure local reactions at the injection site and a thermometer for recording daily temperatures and provide instructions on their use.
- Explain the e-diary technologies available for this study (see Section 8.14), and assist the participant in downloading the study application onto the participant's own device or issue a provisioned device if required. Provide instructions on e-diary completion and ask the participant to complete the reactogenicity e-diary from Day 1 to Day 7, with Day 1 being the day of vaccination and, if utilized, the COVID-19 illness e-diary (to be completed if the participant is diagnosed with COVID-19 or has possible new or increased symptoms, and when he/she receives a reminder, at least weekly).
- Ask the participant to contact the site staff or investigator immediately if he or she experiences any of the following from Day 1 to Day 7 after vaccination (where Day 1 is the day of vaccination) to determine if an unscheduled reactogenicity visit is required:
  - Fever ≥39.0°C.

090177e1968b9275\Approved\Approved On: 18-Mar-2021 07:58 (GMT)

    - Redness or swelling at the injection site measuring greater than 10 cm (>20 measuring device units).
    - Severe pain at the injection site.
    - Any severe systemic event.
- Ask the participant to contact the site staff or investigator if a medically attended event (eg, doctor's visit, emergency room visit) or hospitalization occurs.
- Ask the participant to contact the site staff or investigator (this could be via the COVID-19 illness e-diary) immediately if he or she experiences any symptoms as detailed in Section 8.13.
- Schedule an appointment for the participant to return for the next study visit.
- Remind the participant to bring the e-diary to the next visit.
- Complete the source documents.
- The investigator or an authorized designee completes the CRFs and an unblinded dispenser/administrator updates the study intervention accountability records.
- The investigator or appropriately qualified designee reviews the reactogenicity e-diary data online following vaccination to evaluate participant compliance and as part of the ongoing safety review. Daily review is optimal during the active diary period.

8.11.1.3. Visit 2 – Next-Day Follow-up Visit (Vaccination 1) (1 to 3 Days After Visit 1)

- Record AEs as described in Section 8.3.
- Perform a clinical assessment. If the clinical assessment indicates that a physical examination is necessary to comprehensively evaluate the participant, perform a physical examination and record any findings in the source documents and, if clinically significant, record on the AE CRF.
- Collect a blood sample (approximately 10 mL) for hematology and chemistry laboratory tests as described in Section 10.2.
- Record nonstudy vaccinations as described in Section 6.5.
- Confirm details of any of the prohibited medications specified in Section 6.5.1 received by the participant if required for his or her clinical care.
- Discuss contraceptive use as described in Section 10.4.

090177e1968b9275\Approved\Approved On: 18-Mar-2021 07:58 (GMT)

- Ask the participant to contact the site staff or investigator immediately if he or she experiences any of the following from Day 1 to Day 7 after vaccination (where Day 1 is the day of vaccination) to determine if an unscheduled reactogenicity visit is required:
  - Fever ≥39.0°C.
  - Redness or swelling at the injection site measuring greater than 10 cm (>20 measuring device units).
  - Severe pain at the injection site.
  - Any severe systemic event.
- Ask the participant to contact the site staff or investigator if a medically attended event (eg, doctor's visit, emergency room visit) or hospitalization occurs.
- Ask the participant to contact the site staff or investigator (this could be via the COVID-19 illness e-diary) immediately if he or she experiences any symptoms as detailed in Section 8.13.
- Schedule an appointment for the participant to return for the next study visit.
- Remind the participant to bring the e-diary to the next visit.
- Complete the source documents.
- The investigator or an authorized designee completes the CRFs.
- The investigator or appropriately qualified designee reviews the reactogenicity e-diary data online following vaccination to evaluate participant compliance and as part of the ongoing safety review. Daily review is optimal during the active diary period.

8.11.1.4. Visit 3 – 1-Week Follow-up Visit (Vaccination 1) (6 to 8 Days After Visit 1)

- Record AEs as described in Section 8.3.
- Review hematology and chemistry laboratory results and record any AEs in accordance with Appendix 2.
- Perform a clinical assessment. If the clinical assessment indicates that a physical examination is necessary to comprehensively evaluate the participant, perform a physical examination and record any findings in the source documents and, if clinically significant, record on the AE CRF.
- Collect a blood sample (approximately 10 mL) for hematology and chemistry laboratory tests as described in Section 10.2.

090177e1968b9275\Approved\Approved On: 18-Mar-2021 07:58 (GMT)

- Record nonstudy vaccinations as described in Section 6.5.
- Confirm details of any of the prohibited medications specified in Section 6.5.1 received by the participant if required for his or her clinical care.
- Discuss contraceptive use as described in Section 10.4.
- Ask the participant to contact the site staff or investigator immediately if he or she experiences any of the following from Day 1 to Day 7 after vaccination (where Day 1 is the day of vaccination) to determine if an unscheduled reactogenicity visit is required:
    - Fever ≥39.0°C.
    - Redness or swelling at the injection site measuring greater than 10 cm (>20 measuring device units).
    - Severe pain at the injection site.
    - Any severe systemic event.
- Ask the participant to contact the site staff or investigator if a medically attended event (eg, doctor's visit, emergency room visit) or hospitalization occurs.
- Ask the participant to contact the site staff or investigator (this could be via the COVID-19 illness e-diary) immediately if he or she experiences any symptoms as detailed in Section 8.13.
- Schedule an appointment for the participant to return for the next study visit.
- Remind the participant to bring the e-diary to the next visit.
- Complete the source documents.
- The investigator or an authorized designee completes the CRFs.
- The investigator or appropriately qualified designee reviews the reactogenicity e-diary data online following vaccination to evaluate participant compliance and as part of the ongoing safety review. Daily review is optimal during the active diary period.

8.11.1.5. Visit 4 – Vaccination 2 (19 to 23 Days After Visit 1)

It is anticipated that the procedures below will be conducted in a stepwise manner; ensure that procedures listed prior to administration of the vaccine are conducted prior to vaccination.

- Record AEs as described in Section 8.3.

090177e1968b9275\Approved\Approved On: 18-Mar-2021 07:58 (GMT)

- Review the participant's reactogenicity e-diary data. Collect stop dates of any reactogenicity e-diary events ongoing on the last day that the reactogenicity e-diary was completed and record stop dates in the CRF if required.

- Review hematology and chemistry laboratory results and record any AEs in accordance with Appendix 2.

- Perform a clinical assessment. If the clinical assessment indicates that a physical examination is necessary to comprehensively evaluate the participant, perform a physical examination and record any findings in the source documents and, if clinically significant, record on the AE CRF.

- Perform urine pregnancy test on WOCBP as described in Section 8.2.4.

- Discuss contraceptive use as described in Section 10.4.

- Collect a blood sample (approximately 10 mL) for hematology and chemistry laboratory tests as described in Section 10.2.

- Record nonstudy vaccinations as described in Section 6.5.

- Confirm details of any of the prohibited medications specified in Section 6.5.1 received by the participant if required for his or her clinical care.

- Ensure and document that all of the inclusion criteria and none of the exclusion criteria are met. Participants who have a positive SARS-CoV-2 NAAT result prior to the Vaccination 2 visit should be handled as described in Section 8.15.

- Measure the participant's body temperature (axillary).

- Ensure that the participant meets none of the temporary delay criteria as described in Section 5.5.

- Collect a blood sample (approximately 25 mL) for immunogenicity testing.

- Obtain a nasal (midturbinate) swab (collected by site staff).

- Unblinded site staff member(s) will dispense/administer 1 dose of study intervention into the deltoid muscle of the preferably nondominant arm. Please refer to the IP manual for further instruction on this process.

- Blinded site staff must observe the participant for at least 30 minutes after study intervention administration for any acute reactions. Record any acute reactions (including time of onset) in the participant's source documents and on the AE page of the CRF, and on an SAE form as applicable.

090177e1968b9275\Approved\Approved On: 18-Mar-2021 07:58 (GMT)

- Ensure the participant has a measuring device to measure local reactions at the injection site and a thermometer for recording daily temperatures.
- Ensure the participant remains comfortable with his or her chosen e-diary platform, confirm instructions on e-diary completion, and ask the participant to complete the reactogenicity e-diary from Day 1 to Day 7, with Day 1 being the day of vaccination.
- Ask the participant to contact the site staff or investigator immediately if he or she experiences any of the following from Day 1 to Day 7 after vaccination (where Day 1 is the day of vaccination) to determine if an unscheduled reactogenicity visit is required:
  - Fever ≥39.0°C.
  - Redness or swelling at the injection site measuring greater than 10 cm (>20 measuring device units).
  - Severe pain at the injection site.
  - Any severe systemic event.
- Ask the participant to contact the site staff or investigator if a medically attended event (eg, doctor's visit, emergency room visit) or hospitalization occurs.
- Ask the participant to contact the site staff or investigator (this could be via the COVID-19 illness e-diary) immediately if he or she experiences any symptoms as detailed in Section 8.13.
- Schedule an appointment for the participant to return for the next study visit.
- Remind the participant to bring the e-diary to the next visit.
- Complete the source documents.
- The investigator or an authorized designee completes the CRFs.
- The investigator or appropriately qualified designee reviews the reactogenicity e-diary data online following vaccination to evaluate participant compliance and as part of the ongoing safety review. Daily review is optimal during the active diary period.

8.11.1.6. Visit 5 – 1-Week Follow-up Visit (6 to 8 Days After Visit 4)

- Record AEs as described in Section 8.3.
- Review the participant's reactogenicity e-diary data. Collect stop dates of any reactogenicity e-diary events ongoing on the last day that the reactogenicity e-diary was completed and record stop dates in the CRF if required.

090177e1968b9275\Approved\Approved On: 18-Mar-2021 07:58 (GMT)

- Review hematology and chemistry laboratory results and record any AEs in accordance with Appendix 2.
- Record nonstudy vaccinations as described in Section 6.5.
- Confirm details of any of the prohibited medications specified in Section 6.5.1 received by the participant if required for his or her clinical care.
- Discuss contraceptive use as described in Section 10.4.
- Collect a blood sample (approximately 10 mL) for hematology and chemistry laboratory tests as described in Section 10.2.
- Collect a blood sample (approximately 25 mL) for immunogenicity testing.
- Ask the participant to contact the site staff or investigator if a medically attended event (eg, doctor's visit, emergency room visit) or hospitalization occurs.
- Ask the participant to contact the site staff or investigator (this could be via the COVID-19 illness e-diary) immediately if he or she experiences any symptoms as detailed in Section 8.13.
- Schedule an appointment for the participant to return for the next study visit.
- Complete the source documents.
- The investigator or an authorized designee completes the CRFs.

8.11.1.7. Visit 6 – 2-Week Follow-up Visit (12 to 16 Days After Visit 4)

- Record AEs as described in Section 8.3.
- Review the participant's reactogenicity e-diary data. Collect stop dates of any reactogenicity e-diary events ongoing on the last day that the reactogenicity e-diary was completed and record stop dates in the CRF if required.
- Review hematology and chemistry laboratory results and record any AEs in accordance with Appendix 2.
- Record nonstudy vaccinations as described in Section 6.5.
- Confirm details of any of the prohibited medications specified in Section 6.5.1 received by the participant if required for his or her clinical care.
- Discuss contraceptive use as described in Section 10.4.
- Collect a blood sample (approximately 25 mL) for immunogenicity testing.

090177e1968b9275\Approved\Approved On: 18-Mar-2021 07:58 (GMT)

- Ask the participant to contact the site staff or investigator if a medically attended event (eg, doctor's visit, emergency room visit) or hospitalization occurs.
- Ask the participant to contact the site staff or investigator (this could be via the COVID-19 illness e-diary) immediately if he or she experiences any symptoms as detailed in Section 8.13.
- Schedule an appointment for the participant to return for the next study visit.
- Complete the source documents.
- The investigator or an authorized designee completes the CRFs.

8.11.1.8. Visit 7 – 1-Month Follow-up Visit (28 to 35 Days After Visit 4)

- Record AEs as described in Section 8.3.
- Record nonstudy vaccinations as described in Section 6.5.
- Confirm details of any of the prohibited medications specified in Section 6.5.1 received by the participant if required for his or her clinical care.
- Discuss contraceptive use as described in Section 10.4.
- Collect a blood sample (approximately 25 mL) for immunogenicity testing.
- Ask the participant to contact the site staff or investigator if a medically attended event (eg, doctor's visit, emergency room visit) or hospitalization occurs.
- Ask the participant to contact the site staff or investigator (this could be via the COVID-19 illness e-diary) immediately if he or she experiences any symptoms as detailed in Section 8.13.
- Schedule an appointment for the participant to return for the next study visit.
- Complete the source documents.
- The investigator or an authorized designee completes the CRFs.

8.11.1.9. Between Visits 7 and 8

All participants will be contacted by the site and unblinded to study intervention allocation to allow placebo recipients the opportunity to receive BNT162b2 as part of the study. If the participant originally received placebo and wants to receive BNT162b2, he or she will follow the procedures in Section 8.16 and the SoA in Section 1.3.3.

090177e1968b9275\Approved\Approved On: 18-Mar-2021 07:58 (GMT)

8.11.1.10. Visit 8 – 6-Month Follow-up Visit (154 to 168 Days After Visit 4): Only for Those Participants Who Originally Received BNT162b2 or Placebo Recipients Who Decline BNT162b2

- Record SAEs as described in Section 8.3.
- Record nonstudy vaccinations as described in Section 6.5.
- Confirm details of any of the prohibited medications specified in Section 6.5.1 received by the participant if required for his or her clinical care.
- Collect a blood sample (approximately 25 mL) for immunogenicity testing.
- Ask the participant to contact the site staff or investigator if a medically attended event (eg, doctor's visit, emergency room visit) or hospitalization occurs.
- Ask the participant to contact the site staff or investigator (this could be via the COVID-19 illness e-diary) immediately if he or she experiences any symptoms as detailed in Section 8.13.
- Schedule an appointment for the participant to return for the next study visit.
- Complete the source documents.
- The investigator or an authorized designee completes the CRFs.
- Record any AEs that occur within the 48 hours after the blood draw as described in Section 8.3.

8.11.1.11. Visit 9 – 12-Month Follow-up Visit (350 to 378 Days After Visit 4): Only for Those Participants Who Originally Received BNT162b2 or Placebo Recipients Who Decline BNT162b2

- Record SAEs as described in Section 8.3.
- Collect a blood sample (approximately 25 mL) for immunogenicity testing.
- Confirm details of any of the prohibited medications specified in Section 6.5.1 received by the participant if required for his or her clinical care.
- Collect the participant's e-diary or assist the participant to remove the study application from his or her own personal device.
- Complete the source documents.
- The investigator or an authorized designee completes the CRFs.

090177e1968b9275\Approved\Approved On: 18-Mar-2021 07:58 (GMT)

- Record any AEs that occur within the 48 hours after the blood draw as described in Section 8.3.

8.11.2. Standard Participants

8.11.2.1. Visit 1 – Vaccination 1 (Day 1)

Before enrollment and before any study-related procedures are performed, voluntary, written, study-specific informed consent will be obtained from the participant. Each signature on the ICD must be personally dated by the signatory. The investigator or his or her designee will also sign the ICD. A copy of the signed and dated ICD must be given to the participant. The source data must reflect that the informed consent was obtained before participation in the study.

It is anticipated that the procedures below will be conducted in a stepwise manner.

- Assign a single participant number using the IRT system.
- Obtain the participant's demography (including date of birth, sex, race, and ethnicity). The full date of birth will be collected to critically evaluate the immune response and safety profile by age.
- Obtain any medical history of clinical significance.
- Perform a clinical assessment. If the clinical assessment indicates that a physical examination is necessary to comprehensively evaluate the participant, perform a physical examination and record any findings in the source documents and, if clinically significant, record on the medical history CRF.
- Measure the participant's height and weight.
- Perform urine pregnancy test on WOCBP as described in Section 8.2.4.
- Discuss contraceptive use as described in Section 10.4.
- Record nonstudy vaccinations as described in Section 6.5.
- Ensure and document that all of the inclusion criteria and none of the exclusion criteria are met.
- Measure the participant's body temperature (axillary).
- Ensure that the participant meets none of the temporary delay criteria as described in Section 5.5.
- Record AEs as described in Section 8.3.

090177e1968b9275\Approved\Approved On: 18-Mar-2021 07:58 (GMT)

- Obtain the participant's randomization number and study intervention allocation using the IRT system. Only an unblinded site staff member may obtain this information.
- Collect a blood sample (approximately 25 mL) for immunogenicity testing.
- Obtain a nasal (midturbinate) swab (collected by site staff).
- Unblinded site staff member(s) will dispense/administer 1 dose of study intervention into the deltoid muscle of the preferably nondominant arm. Please refer to the IP manual for further instruction on this process.
- Blinded site staff must observe the participant for at least 30 minutes after study intervention administration for any acute reactions. Record any acute reactions (including time of onset) in the participant's source documents and on the AE page of the CRF, and on an SAE form as applicable.
- Issue a measuring device to measure local reactions at the injection site and a thermometer for recording daily temperatures and provide instructions on their use.
- Explain the e-diary technologies available for this study (see Section 8.14), and assist the participant in downloading the study application onto the participant's own device or issue a provisioned device if required. Provide instructions on e-diary completion and ask the participant to complete the reactogenicity e-diary from Day 1 to Day 7, with Day 1 being the day of vaccination and, if utilized, the COVID-19 illness e-diary (to be completed if the participant is diagnosed with COVID-19 or has possible new or increased symptoms, and when he/she receives a reminder, at least weekly).
- Ask the participant to contact the site staff or investigator immediately if he or she experiences any of the following from Day 1 to Day 7 after vaccination (where Day 1 is the day of vaccination) to determine if an unscheduled reactogenicity visit is required:
    - Fever ≥39.0°C.
    - Redness or swelling at the injection site measuring greater than 10 cm (>20 measuring device units).
    - Severe pain at the injection site.
    - Any severe systemic event.
- Ask the participant to contact the site staff or investigator if a medically attended event (eg, doctor's visit, emergency room visit) or hospitalization occurs.
- Ask the participant to contact the site staff or investigator (this could be via the COVID-19 illness e-diary) immediately if he or she experiences any symptoms as detailed in Section 8.13.

090177e1968b9275\Approved\Approved On: 18-Mar-2021 07:58 (GMT)

- Schedule an appointment for the participant to return for the next study visit.
- Remind the participant to bring the e-diary to the next visit.
- Complete the source documents.
- The investigator or an authorized designee completes the CRFs and an unblinded dispenser/administrator updates the study intervention accountability records.
- The investigator or appropriately qualified designee reviews the reactogenicity e-diary data online following vaccination to evaluate participant compliance and as part of the ongoing safety review. Daily review is optimal during the active diary period.

8.11.2.2. Visit 2 – Vaccination 2 (19 to 23 Days After Visit 1)

It is anticipated that the procedures below will be conducted in a stepwise manner; ensure that procedures listed prior to administration of the vaccine are conducted prior to vaccination.

- Record AEs as described in Section 8.3.
- Review the participant's reactogenicity e-diary data. Collect stop dates of any reactogenicity e-diary events ongoing on the last day that the reactogenicity e-diary was completed and record stop dates in the CRF if required.
- Perform urine pregnancy test on WOCBP as described in Section 8.2.4.
- Discuss contraceptive use as described in Section 10.4.
- Record nonstudy vaccinations as described in Section 6.5.
- Confirm details of any of the prohibited medications specified in Section 6.5.1 received by the participant if required for his or her clinical care.
- Ensure and document that all of the inclusion criteria and none of the exclusion criteria are met. If not, the participant should not receive further study intervention but will remain in the study to be evaluated for safety and immunogenicity (see Section 7.1). Participants who have a positive SARS-CoV-2 NAAT result prior to the Vaccination 2 visit should be handled as described in Section 8.15.
- Measure the participant's body temperature (axillary).
- Ensure that the participant meets none of the temporary delay criteria as described in Section 5.5.
- Collect a blood sample (approximately 25 mL) for immunogenicity testing.

090177e1968b9275\Approved\Approved On: 18-Mar-2021 07:58 (GMT)

- Obtain a nasal (midturbinate) swab (collected by site staff).
- Unblinded site staff member(s) will dispense/administer 1 dose of study intervention into the deltoid muscle of the preferably nondominant arm. Please refer to the IP manual for further instruction on this process.
- Blinded site staff must observe the participant for at least 30 minutes after study intervention administration for any acute reactions. Record any acute reactions (including time of onset) in the participant's source documents and on the AE page of the CRF, and on an SAE form as applicable.
- Ensure the participant has a measuring device to measure local reactions at the injection site and a thermometer for recording daily temperatures.
- Ensure the participant remains comfortable with his or her chosen e-diary platform, confirm instructions on e-diary completion, and ask the participant to complete the reactogenicity e-diary from Day 1 to Day 7, with Day 1 being the day of vaccination.
- Ask the participant to contact the site staff or investigator immediately if he or she experiences any of the following from Day 1 to Day 7 after vaccination (where Day 1 is the day of vaccination) to determine if an unscheduled reactogenicity visit is required:
    - Fever ≥39.0°C.
    - Redness or swelling at the injection site measuring greater than 10 cm (>20 measuring device units).
    - Severe pain at the injection site.
    - Any severe systemic event.
- Ask the participant to contact the site staff or investigator if a medically attended event (eg, doctor's visit, emergency room visit) or hospitalization occurs.
- Ask the participant to contact the site staff or investigator (this could be via the COVID-19 illness e-diary) immediately if he or she experiences any symptoms as detailed in Section 8.13.
- Schedule an appointment for the participant to return for the next study visit.
- Remind the participant to bring the e-diary to the next visit.
- Complete the source documents.
- The investigator or an authorized designee completes the CRFs and an unblinded dispenser/administrator updates the study intervention accountability records.

090177e1968b9275\Approved\Approved On: 18-Mar-2021 07:58 (GMT)

- The investigator or appropriately qualified designee reviews the reactogenicity e-diary data online following vaccination to evaluate participant compliance and as part of the ongoing safety review. Daily review is optimal during the active diary period.

8.11.2.3. Visit 3 – 1-Week Follow-up Visit (6 to 8 Days After Visit 2)

- Record AEs as described in Section 8.3.
- Review the participant's reactogenicity e-diary data. Collect stop dates of any reactogenicity e-diary events ongoing on the last day that the reactogenicity e-diary was completed and record stop dates in the CRF if required.
- Record nonstudy vaccinations as described in Section 6.5.
- Confirm details of any of the prohibited medications specified in Section 6.5.1 received by the participant if required for his or her clinical care.
- Discuss contraceptive use as described in Section 10.4.
- Collect a blood sample (approximately 25 mL) for immunogenicity testing.
- Ask the participant to contact the site staff or investigator if a medically attended event (eg, doctor's visit, emergency room visit) or hospitalization occurs.
- Ask the participant to contact the site staff or investigator (this could be via the COVID-19 illness e-diary) immediately if he or she experiences any symptoms as detailed in Section 8.13.
- Schedule an appointment for the participant to return for the next study visit.
- Complete the source documents.
- The investigator or an authorized designee completes the CRFs.

8.11.2.4. Visit 4 – 2-Week Follow-up Visit (12 to 16 Days After Visit 2)

- Record AEs as described in Section 8.3.
- Review the participant's reactogenicity e-diary data. Collect stop dates of any reactogenicity e-diary events ongoing on the last day that the reactogenicity e-diary was completed and record stop dates in the CRF if required.
- Record nonstudy vaccinations as described in Section 6.5.
- Confirm details of any of the prohibited medications specified in Section 6.5.1 received by the participant if required for his or her clinical care.

090177e1968b9275\Approved\Approved On: 18-Mar-2021 07:58 (GMT)

- Discuss contraceptive use as described in Section 10.4.
- Collect a blood sample (approximately 25 mL) for immunogenicity testing.
- Ask the participant to contact the site staff or investigator if a medically attended event (eg, doctor's visit, emergency room visit) or hospitalization occurs.
- Ask the participant to contact the site staff or investigator (this could be via the COVID-19 illness e-diary) immediately if he or she experiences any symptoms as detailed in Section 8.13.
- Schedule an appointment for the participant to return for the next study visit.
- Complete the source documents.
- The investigator or an authorized designee completes the CRFs.

8.11.2.5. Visit 5 – 1-Month Follow-up Visit (28 to 35 Days After Visit 2)

- Record AEs as described in Section 8.3.
- Record nonstudy vaccinations as described in Section 6.5.
- Confirm details of any of the prohibited medications specified in Section 6.5.1 received by the participant if required for his or her clinical care.
- Discuss contraceptive use as described in Section 10.4.
- Collect a blood sample (approximately 25 mL) for immunogenicity testing.
- Ask the participant to contact the site staff or investigator if a medically attended event (eg, doctor's visit, emergency room visit) or hospitalization occurs.
- Ask the participant to contact the site staff or investigator (this could be via the COVID-19 illness e-diary) immediately if he or she experiences any symptoms as detailed in Section 8.13.
- Schedule an appointment for the participant to return for the next study visit.
- Complete the source documents.
- The investigator or an authorized designee completes the CRFs.

090177e1968b9275\Approved\Approved On: 18-Mar-2021 07:58 (GMT)

8.11.2.6. Between Visits 5 and 6

All participants will be contacted by the site and unblinded to study intervention allocation to allow placebo recipients the opportunity to receive BNT162b2 as part of the study. If the participant originally received placebo and wants to receive BNT162b2, he or she will follow the procedures in Section 8.16 and the SoA in Section 1.3.3.

8.11.2.7. Visit 6 – 6-Month Follow-up Visit (154 to 168 Days After Visit 2): Only for Those Participants Who Originally Received BNT162b2 or Placebo Recipients Who Decline BNT162b2

- Record SAEs as described in Section 8.3.
- Record nonstudy vaccinations as described in Section 6.5.
- Confirm details of any of the prohibited medications specified in Section 6.5.1 received by the participant if required for his or her clinical care.
- Collect a blood sample (approximately 25 mL) for immunogenicity testing.
- Ask the participant to contact the site staff or investigator if a medically attended event (eg, doctor's visit, emergency room visit) or hospitalization occurs.
- Ask the participant to contact the site staff or investigator (this could be via the COVID-19 illness e-diary) immediately if he or she experiences any symptoms as detailed in Section 8.13.
- Schedule an appointment for the participant to return for the next study visit.
- Complete the source documents.
- The investigator or an authorized designee completes the CRFs.
- Record any AEs that occur within the 48 hours after the blood draw as described in Section 8.3.

8.11.2.8. Visit 7 – 12-Month Follow-up Visit (350 to 378 Days After Visit 2): Only for Those Participants Who Originally Received BNT162b2 or Placebo Recipients Who Decline BNT162b2

- Record SAEs as described in Section 8.3.
- Collect a blood sample (approximately 25 mL) for immunogenicity testing.
- Confirm details of any of the prohibited medications specified in Section 6.5.1 received by the participant if required for his or her clinical care.

090177e1968b9275\Approved\Approved On: 18-Mar-2021 07:58 (GMT)

- Collect the participant's e-diary or assist the participant to remove the study application from his or her own personal device.
- Complete the source documents.
- The investigator or an authorized designee completes the CRFs.
- Record any AEs that occur within the 48 hours after the blood draw as described in Section 8.3.

8.12. Unscheduled Visit for a Grade 3 or Suspected Grade 4 Reaction

If a Grade 3 local reaction (Section 8.2.2.2), systemic event (Section 8.2.2.3), or fever (Section 8.2.2.4) is reported in the reactogenicity e-diary, a telephone contact should occur to ascertain further details and determine whether a site visit is clinically indicated. If a suspected Grade 4 local reaction (Section 8.2.2.2), systemic event (Section 8.2.2.3), or fever (Section 8.2.2.4) is reported in the reactogenicity e-diary, a telephone contact or site visit should occur to confirm whether the event meets the criteria for Grade 4.

A site visit must be scheduled as soon as possible to assess the participant unless any of the following is true:

- The participant is unable to attend the unscheduled visit.
- The local reaction/systemic event is no longer present at the time of the telephone contact.
- The participant recorded an incorrect value in the reactogenicity e-diary (confirmation of a reactogenicity e-diary data entry error).
- The PI or authorized designee determined it was not needed.

This telephone contact will be recorded in the participant's source documentation and the CRF.

If the participant is unable to attend the unscheduled visit, or the PI or authorized designee determined it was not needed, any ongoing local reactions/systemic events must be assessed at the next study visit.

During the unscheduled visit, the reactions/events should be assessed by the investigator or a medically qualified member of the study staff such as a study physician or a study nurse, as applicable to the investigator's local practice, who will:

- Measure body temperature (°C) (axillary).
- Measure minimum and maximum diameters of redness (if present).

090177e1968b9275\Approved\Approved On: 18-Mar-2021 07:58 (GMT)

- Measure minimum and maximum diameters of swelling (if present).
- Assess injection site pain (if present) in accordance with the grades provided in Section 8.2.2.2.
- Assess systemic events (if present) in accordance with the grades provided in Section 8.2.2.3.
- Assess for other findings associated with the reaction and record on the AE page of the CRF, if appropriate.

The investigator or an authorized designee will complete the unscheduled visit assessment page of the CRF.

8.13. COVID-19 Disease Surveillance

If a participant experiences any of the following, he or she is instructed to contact the site immediately and, if confirmed, participate in a telehealth visit as soon as possible, optimally within 3 days of symptom onset. During the 7 days following each vaccination, potential COVID-19 symptoms that overlap with solicited systemic events (ie, fever, chills, new or increased muscle pain, diarrhea, vomiting) should not trigger a potential COVID-19 illness visit unless, in the investigator's opinion, the clinical picture is more indicative of a possible COVID-19 illness than vaccine reactogenicity. Participants may utilize a COVID-19 illness e-diary via an application (see Section 8.14) installed on a provisioned device or on the participant's own personal device to prompt him/her to report any symptoms. Note that this does not substitute for a participant's routine medical care. Therefore, participants should be encouraged to seek care, if appropriate, from their usual provider.

- A diagnosis of COVID-19;
- Fever;
- New or increased cough;
- New or increased shortness of breath;
- Chills;
- New or increased muscle pain;
- New loss of taste/smell;
- Sore throat;
- Diarrhea;
- Vomiting.

090177e1968b9275\Approved\Approved On: 18-Mar-2021 07:58 (GMT)

090177e1968b9275\Approved\Approved On: 18-Mar-2021 07:58 (GMT)

8.13.1. Potential COVID-19 Illness Visit (Optimally Within 3 Days After Potential COVID-19 Illness Onset)

This visit is expected to involve the sharing of healthcare information and services via telecommunication technologies (eg, audio, video, videoconferencing software) remotely, thus allowing the participant and investigator to communicate on aspects of clinical care.

As a participant's COVID-19 illness may evolve over time, several contacts may be required to obtain the following information:

- Record AEs, as appropriate as described in Section 8.3.
- Confirm details of any of the prohibited medications specified in Section 6.5.1 received by the participant if required for his or her clinical care.
- Collect local test results of COVID-19 and local diagnosis of COVID-19 as available.
- Complete the source documents.
- The investigator or an authorized designee completes the CRFs.

8.13.2. Potential COVID-19 Convalescent Visit (28 to 35 Days After Potential COVID-19 Illness Visit)

This visit is expected to involve the sharing of healthcare information and services via telecommunication technologies (eg, audio, video, videoconferencing software) remotely, thus allowing the participant and investigator to communicate on aspects of clinical care.

- Record AEs, as appropriate as described in Section 8.3.
- Confirm details of any of the prohibited medications specified in Section 6.5.1 received by the participant if required for his or her clinical care.
- Complete the source documents.
- The investigator or an authorized designee completes the CRFs.

8.14. Communication and Use of Technology

In a study of this nature that requires illness events to be reported outside of scheduled study visits, it is vital that communication between the study site and the participant is maintained to ensure that endpoint events are not missed. This study will employ various methods, tailored to the individual participant, to ensure that communication is maintained and study information can be transmitted securely. Using appropriate technology, such as a study application, a communication pathway between the participant and the study site staff will be established. The participant may be able to utilize his or her own devices to access this technology, or use a device provided by the sponsor. Traditional methods of telephone communication will also be available. The technology solution may facilitate the following:

- Contact with the investigator, including the ability of the participant to report whether or not he or she has experienced symptoms that could represent a potential COVID-19 illness (COVID-19 illness e-diary; see Section 8.13).
- An alert in the event that the participant is hospitalized.
- Visit reminders.
- Messages of thanks and encouragement from the study team.
- A platform for recording local reactions and systemic events (reactogenicity e-diary) – see Section 8.2.2.

If a participant is not actively completing either the reactogenicity or COVID-19 illness e-diary, the investigator or designee is required to contact the participant to ascertain why and also to obtain details of any missed events.

8.15. SARS-CoV-2 NAAT Results From Vaccination 1 and Vaccination 2 Visits

Nasal (midturbinate) swabs for SARS-CoV-2 NAAT are obtained at Visits 1 and 4 (clinical laboratory subset) or Visits 1 and 2 (standard participants) to describe the immune responses elicited by a prophylactic BNT162b2 vaccine in participants with or without confirmed COVID-19 before Dose 2 and during the study (the secondary and exploratory objectives).

Research laboratory–generated positive results from the Vaccination 1 and Vaccination 2 visit swabs will be provided to the site once available, but this will not be in real time. The investigator must report the positive result to the community health center according to the local regulation in Japan.

Participants who have a positive SARS-CoV-2 NAAT result prior to the Vaccination 2 visit (Visit 2 for standard participants, Visit 4 for the clinical laboratory subset) should be handled as follows:

- Positive SARS-CoV-2 test with no symptoms, either at Visit 1 or any time between Visit 1 and the Vaccination 2 visit: A positive test in an asymptomatic participant does not meet exclusion criterion 5; therefore, Vaccination 2 should proceed as normal.
- Confirmed COVID-19 (ie, symptoms and positive SARS-CoV-2 test): This meets exclusion criterion 5; therefore, Vaccination 2 should not be given but the participant should remain in the study.

8.16. Procedures for Administration of BNT162b2 to Those Originally Assigned to Placebo After Approval of Protocol Amendment 3

All participants will be contacted by the site and unblinded to study intervention allocation to allow placebo recipients the opportunity to receive BNT162b2 as part of the study. Visit 101 could occur at the same time as the original Visit 8 for the clinical laboratory subset or

090177e1968b9275\Approved\Approved On: 18-Mar-2021 07:58 (GMT)

Visit 6 for the standard participants, if the participants originally received placebo and want to receive BNT162b2.

8.16.1. Visit 101 – Vaccination 3 (After Protocol Amendment 3 is Approved by the IRB)

Before vaccination and before any study-related procedures are performed, voluntary, written, informed consent (via an ICD addendum) will be obtained from the participant. Each signature on the ICD addendum must be personally dated by the signatory. The investigator or his or her designee will also sign the ICD addendum. A copy of the signed and dated ICD addendum must be given to the participant.

- Confirm the participant originally received only placebo at Vaccinations 1 and 2. Secondary confirmation by another site staff member is required.
- Perform a urine pregnancy test on WOCBP as described in Section 8.2.4.
- Discuss contraceptive use as described in Section 10.4.
- Record nonstudy vaccinations as described in Section 6.5.
- Confirm details of any of the prohibited medications, specified in Section 6.5.1, received by the participant if required for his or her clinical care.
- Ensure and document that inclusion criteria 2, 3, and 4 are met and exclusion criteria 1, 3, 6, 7, 8, 9, 10, 12, 13, and 15 are not met. If, in the investigator's judgment, vaccination is in the best interests of the participant, vaccination may proceed, even if inclusion criteria are not met and exclusion criteria are met. Such exceptions should be recorded in the participant's source documents.
- Measure the participant's body temperature (axillary).
- Ensure that the participant meets none of the temporary delay criteria as described in Section 5.5.
- Record AEs as described in Section 8.3.
- Collect a blood sample (approximately 25 mL) for immunogenicity testing. If a sample for this purpose has already been collected in the previous 7 days (eg, per the procedures at Visit 8 for the clinical laboratory subset or Visit 6 for the standard participants), a second sample need not be collected.
- Obtain the participant's vaccine vial allocation using the IRT system.
- Site staff member(s) will dispense/administer 1 dose of BNT162b2 into the deltoid muscle of the preferably nondominant arm.

090177e1968b9275\Approved\Approved On: 18-Mar-2021 07:58 (GMT)

- Site staff must observe the participant for at least 30 minutes after BNT162b2 administration for any acute reactions. Record any acute reactions (including time of onset) in the participant's source documents and on the AE page of the CRF, and on an SAE form as applicable.
- Ask the participant to contact the site staff or investigator if a medically attended event (eg, doctor's visit, emergency room visit) or hospitalization occurs.
- Ask the participant to contact the site staff or investigator (this could be via the COVID-19 illness e-diary) immediately if the participant experiences any respiratory symptoms as detailed in Section 8.13.
- Schedule an appointment for the participant to return for the next study visit.
- Complete the source documents.
- The investigator or an authorized designee completes the CRFs and the dispenser/administrator updates the study intervention accountability records.

8.16.2. Visit 102 – Vaccination 4 (19 to 23 Days After Visit 101)

It is anticipated that the procedures below will be conducted in a stepwise manner.

- Record AEs as described in Section 8.3.
- Perform a urine pregnancy test on WOCBP as described in Section 8.2.4.
- Discuss contraceptive use as described in Section 10.4.
- Record nonstudy vaccinations as described in Section 6.5.
- Confirm details of any of the prohibited medications, specified in Section 6.5.1, received by the participant if required for his or her clinical care.
- Ensure and document that inclusion criteria 2, 3, and 4 are met and exclusion criteria 1, 3, 6, 7, 8, 9, 10, 12, 13, and 15 are not met. If, in the investigator's judgment, vaccination is in the best interests of the participant, vaccination may proceed, even if inclusion criteria are not met and exclusion criteria are met. Such exceptions should be recorded in the participant's source documents.
- Measure the participant's body temperature (axillary).
- Ensure that the participant meets none of the temporary delay criteria as described in Section 5.5.
- Obtain the participant's vaccine vial allocation using the IRT system.

090177e1968b9275\Approved\Approved On: 18-Mar-2021 07:58 (GMT)

- Site staff member(s) will dispense/administer 1 dose of study intervention into the deltoid muscle of the preferably nondominant arm. Please refer to the IP manual for further instruction on this process.

- Site staff must observe the participant for at least 30 minutes after study intervention administration for any acute reactions. Record any acute reactions (including time of onset) in the participant's source documents and on the AE page of the CRF, and on an SAE form as applicable.

- Ask the participant to contact the site staff or investigator if a medically attended event (eg, doctor's visit, emergency room visit) or hospitalization occurs.

- Ask the participant to contact the site staff or investigator (this could be via the COVID-19 illness e-diary) immediately if the participant experiences any respiratory symptoms as detailed in Section 8.13.

- Schedule an appointment for the participant to return for the next study contact.

- Complete the source documents.

- The investigator or an authorized designee completes the CRFs and the dispenser/administrator updates the study intervention accountability records.

8.16.3. Visit 103 – 1-Month After Vaccination 4 Follow-up Telephone Contact (28 to 35 Days After Visit 102)

- Contact the participant by telephone but, if desired, may be conducted in person.

- Record AEs as described in Section 8.3.

- Record nonstudy vaccinations as described in Section 6.5.

- Confirm details of any of the prohibited medications, specified in Section 6.5.1, received by the participant if required for his or her clinical care.

- Ask the participant to contact the site staff or investigator if a medically attended event (eg, doctor's visit, emergency room visit) or hospitalization occurs.

- Ask the participant to contact the site staff or investigator (this could be via the COVID-19 illness e-diary) immediately if the participant experiences any respiratory symptoms as detailed in Section 8.13.

- Schedule an appointment to call the participant by telephone for the next study contact.

- Complete the source documents.

090177e1968b9275\Approved\Approved On: 18-Mar-2021 07:58 (GMT)

- The investigator or an authorized designee completes the CRFs.

8.16.4. Visit 104 – 6-Months After Vaccination 4 Follow-up Telephone Contact (175 to 189 Days After Visit 102)

- Contact the participant by telephone but, if desired, may be conducted in person.
- Record SAEs as described in Section 8.3.
- Record nonstudy vaccinations as described in Section 6.5.
- Confirm details of any of the prohibited medications, specified in Section 6.5.1, received by the participant if required for his or her clinical care.
- Collect the participant's e-diary or assist the participant to remove the study application from his or her own personal device.
- Inform the participant that his or her study participation has completed.
- Complete the source documents.
- The investigator or an authorized designee completes the CRFs.

9. STATISTICAL CONSIDERATIONS

Detailed methodology for summary and statistical analyses of the data collected in this study is outlined here and further detailed in a SAP, which will be maintained by the sponsor. The SAP may modify what is outlined in the protocol where appropriate; however, any major modifications of the primary endpoint definitions or their analyses will also be reflected in a protocol amendment.

9.1. Estimands and Statistical Hypotheses

9.1.1. Estimands

The estimand corresponding to each primary, secondary, and exploratory objective is described in the table in Section 3.

In the primary safety objective evaluations, missing reactogenicity e-diary data will not be imputed. Missing AE dates will be imputed according to Pfizer safety rules. No other missing information will be imputed in the safety analysis.

090177e1968b9275\Approved\Approved On: 18-Mar-2021 07:58 (GMT)

The estimands to evaluate the immunogenicity objectives are based on the evaluable population for immunogenicity (Section 9.3). These estimands estimate the vaccine effect in the hypothetical setting where participants follow the study schedules and protocol requirements as directed. Missing antibody results will not be imputed. Immunogenicity results that are below the LLOQ will be set to 0.5 × LLOQ in the analysis; this may be adjusted once additional data on the assay characteristics become available. Laboratory results will not be imputed for the primary analysis.

9.1.2. Statistical Hypotheses

There are no statistical hypotheses in the study.

9.2. Sample Size Determination

The study size of the study is not based on any formal hypothesis test for a safety or immunogenicity endpoint. All statistical analyses of safety and immunogenicity will be descriptive.

One hundred sixty participants will be randomized in a 3:1 ratio to receive active vaccine (120 participants) or placebo (40 participants). The evaluable immunogenicity population is expected to comprise 144 participants (108: active vaccine, 36: placebo) 1 month after Dose 2 by assuming a nonevaluable rate of about 10%.

Safety Endpoints

For safety outcomes, Table 4 shows the probability of observing at least 1 AE for a given true event rate of a particular AE. Administration of BNT162b2 to 120 participants provides about 70%, 91%, or more than 99% probability of observing at least 1 occurrence of any AE with a true incidence rate of 1.0%, 2.0%, or 5.0%, respectively.

Table 4. Probability of Observing at Least 1 AE by Assumed True Event Rates

Assumed True Event Rate of an AE	N=120
1.0%	0.701
1.5%	0.837
2.0%	0.911
3.0%	0.974
5.0%	0.998

Immunogenicity Endpoints

To assess precision for the immunogenicity results of the primary immunogenicity endpoints, a precision measure for GMT of a given SARS-CoV-2 serum neutralizing titer is defined as the relative distance from the lower limit of the 2-sided 95% CI for the GMT to the GMT, calculated as (lower limit of CI/[GMT]) × 100%. A precision measure of 80% means that the lower 2-sided 95% confidence limit of the GMT is 20% lower than the GMT for that serotype.

090177e1968b9275\Approved\Approved On: 18-Mar-2021 07:58 (GMT)

Currently, immunogenicity data up to 14 days after Dose 2 for the BNT162b1 30-µg group (18-55 years of age) for the C4591001 study are available. Standard deviations (on the natural log scale) obtained from the data are used in the calculations of the confidence limits of GM (Table 5).

With 108 evaluable participants in the active vaccine group, the lower 95% confidence limits of the GMTs would be about 10% lower than the corresponding GMTs.

Table 5. Precision of Each SARS-CoV-2 Assay

Assay	Assumed Standard Deviation (Natural Log)	Evaluable Participants = 108
SARS-CoV-2 serum neutralizing titer	0.58	90%

Note: Standard deviations were derived at 14 days after Dose 2 for the C4591001 study.

9.3. Analysis Sets

For purposes of analysis, the following populations are defined:

Population	Description
Enrolled	All participants who have a signed ICD.
Randomized	All participants who are assigned a randomization number in the IWR system.
Evaluable immunogenicity (primary analysis population)	All eligible randomized participants who receive 2 doses of the vaccine to which they are randomly assigned, with Dose 2 received within the predefined window, have at least 1 valid and determinate immunogenicity result after Dose 2, have blood collection within an appropriate window after Dose 2, and have no other major protocol deviations as determined by the clinician.
All-available immunogenicity	All randomized participants who receive at least 1 dose of the study intervention with at least 1 valid and determinate immunogenicity result after Dose 2.
Safety	All randomized participants who receive at least 1 dose of the study intervention.

9.4. Statistical Analyses

The SAP will be developed and finalized before database lock for any of the planned analyses in Section 9.5.1. It will describe the participant populations to be included in the analyses and the procedures for accounting for missing, unused, and spurious data. This section provides a summary of the planned statistical analyses of the primary, secondary, and exploratory endpoints.

090177e1968b9275\Approved\Approved On: 18-Mar-2021 07:58 (GMT)

9.4.1. Immunogenicity Analyses

The statistical analysis of immunogenicity results will be primarily based on the evaluable immunogenicity population as defined in Section 9.3.

An additional analysis will be performed based on the all-available population if there is a large enough difference in sample size between the all-available immunogenicity population and the evaluable immunogenicity population. Participants will be summarized according to the vaccine group to which they were randomized.

Endpoint	Statistical Analysis Methods
Primary immunogenicity	**GMTs of SARS-CoV-2 serum neutralizing titers**<br><br>For SARS-CoV-2 serum neutralizing titers, the GMTs and 2-sided 95% CIs will be provided for each group at the following time point:<br><br>• 1 Month after Dose 2<br><br>Geometric means will be calculated as the mean of the assay results after making the logarithm transformation and then exponentiating the mean to express results on the original scale. Two-sided 95% CIs will be obtained by taking natural log transforms of titers, calculating the 95% CI with reference to the t-distribution, and then exponentiating the confidence limits.<br><br>**GMFRs of SARS-CoV-2 serum neutralizing titers**<br><br>For SARS-CoV-2 serum neutralizing titers, the GMFRs and 2-sided 95% CIs will be provided for each group at the following time point:<br><br>• To 1 month after Dose 2 from the prevaccination time point<br><br>GMFRs will be limited to participants with nonmissing values prior to the first dose and at the postvaccination time point. The GMFR will be calculated as the mean of the difference of logarithmically transformed assay results (later time point – earlier time point) and exponentiating the mean. The associated 2-sided CIs will be obtained by calculating CIs using Student's t-distribution for the mean difference of the logarithmically transformed assay results and exponentiating the confidence limits.

090177e1968b9275\Approved\Approved On: 18-Mar-2021 07:58 (GMT)

090177e1968b9275\Approved\Approved On: 18-Mar-2021 07:58 (GMT)

Endpoint	Statistical Analysis Methods
Secondary immunogenicity	**GMTs of SARS-CoV-2 serum neutralizing titers**<br><br>For SARS-CoV-2 serum neutralizing titers, GMTs and 2-sided 95% CIs will be provided for each group at each of the following time points:<br><br>• Baseline, 21 days after Dose 1; 7 and 14 days and 6 and 12 months after Dose 2<br><br>GMTs will be calculated using the same methodology as described for the primary endpoints.<br><br>**GMFRs of SARS-CoV-2 serum neutralizing titers**<br><br>For SARS-CoV-2 serum neutralizing titers, the GMFRs and 2-sided 95% CIs will be provided for each group at each of the following time points:<br><br>• To 21 days after Dose 1, and 7 and 14 days and 6 and 12 months after Dose 2, from the prevaccination time point<br><br>GMFRs will be calculated using the same methodology as described for the primary endpoints.<br><br>**GMTs and GMFRs of SARS-CoV-2 serum neutralizing titers for participants with/without confirmed COVID-19 before Dose 2**<br><br>GMTs, GMFRs, and their 2-sided 95% CIs will be provided for each group at each of the time points for participants with and without confirmed COVID-19 before Dose 2.<br><br>GMTs and GMFRs will be calculated using the same methodology as described for the primary endpoints.
Exploratory immunogenicity	**GMTs and GMFRs of SARS-CoV-2 serum neutralizing titers for participants with/without confirmed COVID-19 during the study**<br><br>GMTs, GMFRs, and their 2-sided 95% CIs will be provided for each group at each of the time points for participants with and without confirmed COVID-19 during the study.<br><br>GMTs and GMFRs will be calculated using the same methodology as described for the primary endpoints.

Endpoint	Statistical Analysis Methods
	**Percentage of participants with the immune response (non-S) to SARS-CoV-2 for N-binding antibody at the time points for participants with/without confirmed COVID-19 during the study when data are available**<br><br>The Clopper-Pearson method will be used to calculate the CIs.<br><br>**RCDCs for immunogenicity results**<br><br>Empirical RCDCs will be provided for SARS-CoV-2 serum neutralizing titers after Dose 2.

9.4.2. Safety Analyses

Endpoint	Statistical Analysis Methods
Primary	Descriptive statistics will be provided for each reactogenicity endpoint for each vaccine group. Local reactions and systemic events from Day 1 through Day 7 after each vaccination will be presented by severity cumulatively across severity levels. Descriptive summary statistics will include counts and percentages of participants with the indicated endpoint and the associated Clopper-Pearson 95% CIs.<br><br>For the clinical laboratory subset, descriptive statistics will be provided for abnormal hematology and chemistry laboratory values at 1 and 7 days after Dose 1 and 7 days after Dose 2, including grading shifts in hematology and chemistry laboratory assessments between baseline and 1 and 7 days after Dose 1, and before Dose 2 and 7 days after Dose 2. Descriptive summary statistics will include counts and percentages of participants with the indicated endpoint and the associated Clopper-Pearson 2-sided 95% CIs.<br><br>AEs will be categorized according to Medical Dictionary for Regulatory Activities (MedDRA) terms. A 3-tier approach will be used to summarize AEs. Under this approach AEs are classified into 1 of 3 tiers: (1) Tier 1 events are prespecified events of clinical importance and are identified in a list in the product's safety review plan; (2) Tier 2 events are those that are not Tier 1 but are considered "relatively common"; a MedDRA preferred term is defined as a Tier 2 event if there are at least 4 of participants in at least 1 vaccine group reporting the event; and (3) Tier 3 events are those that are neither Tier 1 nor Tier 2 events. For both Tier 1 and Tier 2 events, 2-sided 95% CIs for the difference between the vaccine and placebo groups in the percentage of participants reporting the events based on the

090177e1968b9275\Approved\Approved On: 18-Mar-2021 07:58 (GMT)

Endpoint	Statistical Analysis Methods
	Miettinen and Nurminen method[9] will be provided. In addition, for Tier 1 events, the asymptotic p-values will also be presented for the difference between groups in the percentage of participants reporting the events, based on the same test statistic and under the assumption that the test statistic is asymptotically normally distributed.<br><br>Descriptive summary statistics (counts, percentages, and associated Clopper-Pearson 95% CIs) will be provided for any AE events for each vaccine group.<br><br>SAEs will be categorized according to MedDRA terms. Counts, percentages, and the associated Clopper-Pearson 95% CIs of SAEs from Dose 1 to 12 months after Dose 2 will be provided for each vaccine group.<br><br>AEs and SAEs reported only for participants originally assigned to placebo who accepted the opportunity to receive BNT162b2 after approval of protocol amendment 3 will be summarized separately.<br><br>The safety analyses are based on the safety population. Participants will be summarized by vaccine group according to the investigational vaccine they actually received. Missing reactogenicity e-diary data will not be imputed; missing AE dates will be handled according to the Pfizer safety rules.
Secondary	Not applicable (N/A)
Exploratory	N/A

9.5. Interim Analyses

No interim analysis is planned in this study.

9.5.1. Analysis Timing

Statistical analyses will be carried out when the following data are available:

- Primary analysis: complete safety and immunogenicity data through 1 month after Dose 2 from all participants

Interim summary at different time points may be considered to support internal decisions for additional visits and to support regulatory interactions.

090177e1968b9275\Approved\Approved On: 18-Mar-2021 07:58 (GMT)

The study final analysis will be performed when all participants have completed the study and all immunogenicity and safety data are available.

9.6. Data Monitoring Committee or Other Independent Oversight Committee

An external DMC will be utilized for this study and will review cumulative unblinded safety data throughout the study in accordance with the charter.

The recommendations made by the DMC to alter the conduct of the study will be forwarded to the appropriate Pfizer personnel for final decision. Pfizer will forward such decisions, which may include summaries of aggregate analyses of safety data, to regulatory authorities, as appropriate.

090177e1968b9275\Approved\Approved On: 18-Mar-2021 07:58 (GMT)

10. SUPPORTING DOCUMENTATION AND OPERATIONAL CONSIDERATIONS

10.1. Appendix 1: Regulatory, Ethical, and Study Oversight Considerations

10.1.1. Regulatory and Ethical Considerations

This study will be conducted in accordance with the protocol and with the following:

- Consensus ethical principles derived from international guidelines including the Declaration of Helsinki and CIOMS International Ethical Guidelines;
- Applicable ICH GCP guidelines;
- Applicable laws and regulations, including applicable privacy laws and GPSP.

The protocol, protocol amendments, ICD, SRSD(s), and other relevant documents (eg, advertisements) must be reviewed and approved by the sponsor and submitted to an IRB/EC by the investigator and reviewed and approved by the IRB/EC before the study is initiated.

Any amendments to the protocol will require IRB/EC approval before implementation of changes made to the study design, except for changes necessary to eliminate an immediate hazard to study participants.

The investigator will be responsible for the following:

- Providing written summaries of the status of the study to the IRB/EC annually or more frequently in accordance with the requirements, policies, and procedures established by the IRB/EC;
- Notifying the IRB/EC of SAEs or other significant safety findings as required by IRB/EC procedures;
- Providing oversight of the conduct of the study at the site and adherence to requirements of 21 CFR, ICH guidelines, the IRB/EC, European regulation 536/2014 for clinical studies (if applicable), and all other applicable local regulations.

10.1.1.1. Reporting of Safety Issues and Serious Breaches of the Protocol or ICH GCP

In the event of any prohibition or restriction imposed (ie, clinical hold) by an applicable regulatory authority in any area of the world, or if the investigator is aware of any new information that might influence the evaluation of the benefits and risks of the study intervention, Pfizer should be informed immediately.

In addition, the investigator will inform Pfizer immediately of any urgent safety measures taken by the investigator to protect the study participants against any immediate hazard, and of any serious breaches of this protocol or of ICH GCP that the investigator becomes aware of.

090177e1968b9275\Approved\Approved On: 18-Mar-2021 07:58 (GMT)

10.1.2. Financial Disclosure

Investigators and subinvestigators will provide the sponsor with sufficient, accurate financial information as requested to allow the sponsor to submit complete and accurate financial certification or disclosure statements to the appropriate regulatory authorities. Investigators are responsible for providing information on financial interests during the course of the study and for 1 year after completion of the study.

10.1.3. Informed Consent Process

The investigator or his/her representative will explain the nature of the study to the participant and answer all questions regarding the study. The participant should be given sufficient time and opportunity to ask questions and to decide whether or not to participate in the trial.

Participants must be informed that their participation is voluntary. Participants will be required to sign a statement of informed consent that meets the requirements of 21 CFR 50, local regulations, ICH guidelines, HIPAA requirements, where applicable, and the IRB/EC or study center.

The investigator must ensure that each study participant is fully informed about the nature and objectives of the study, the sharing of data related to the study, and possible risks associated with participation, including the risks associated with the processing of the participant's personal data.

The participant must be informed that his/her personal study-related data will be used by the sponsor in accordance with local data protection law. The level of disclosure must also be explained to the participant.

The participant must be informed that his/her medical records may be examined by Clinical Quality Assurance auditors or other authorized personnel appointed by the sponsor, by appropriate IRB/EC members, and by inspectors from regulatory authorities.

The investigator further must ensure that each study participant is fully informed about his or her right to access and correct his or her personal data and to withdraw consent for the processing of his or her personal data.

The medical record must include a statement that written informed consent was obtained before the participant was enrolled in the study and the date the written consent was obtained. The authorized person obtaining the informed consent must also sign the ICD.

Participants must be reconsented to the most current version of the ICD(s) during their participation in the study.

A copy of the ICD(s) must be provided to the participant. Participants who are rescreened are required to sign a new ICD.

090177e1968b9275\Approved\Approved On: 18-Mar-2021 07:58 (GMT)

Unless prohibited by local requirements or IRB/EC decision, the ICD will contain a separate section that addresses the use of samples for optional additional research. The optional additional research does not require the collection of any further samples. The investigator or authorized designee will explain to each participant the objectives of the additional research. Participants will be told that they are free to refuse to participate and may withdraw their consent at any time and for any reason during the storage period.

10.1.4. Data Protection

All parties will comply with all applicable laws, including laws regarding the implementation of organizational and technical measures to ensure protection of participant data.

Participants' personal data will be stored at the study site in encrypted electronic and/or paper form and will be password protected or secured in a locked room to ensure that only authorized study staff have access. The study site will implement appropriate technical and organizational measures to ensure that the personal data can be recovered in the event of disaster. In the event of a potential personal data breach, the study site will be responsible for determining whether a personal data breach has in fact occurred and, if so, providing breach notifications as required by law.

To protect the rights and freedoms of participants with regard to the processing of personal data, participants will be assigned a single, participant-specific numerical code. Any participant records or data sets that are transferred to the sponsor will contain the numerical code; participant names will not be transferred. All other identifiable data transferred to the sponsor will be identified by this single, participant-specific code. The study site will maintain a confidential list of participants who participated in the study, linking each participant's numerical code to his or her actual identity and medical record identification. In case of data transfer, the sponsor will protect the confidentiality of participants' personal data consistent with the clinical study agreement and applicable privacy laws.

10.1.5. Dissemination of Clinical Study Data

Pfizer fulfills its commitment to publicly disclose clinical study results through posting the results of studies on www.clinicaltrials.gov (ClinicalTrials.gov), the EudraCT, and/or www.pfizer.com, and other public registries in accordance with applicable local laws/regulations. In addition, Pfizer reports study results outside of the requirements of local laws/regulations pursuant to its SOPs.

In all cases, study results are reported by Pfizer in an objective, accurate, balanced, and complete manner and are reported regardless of the outcome of the study or the country in which the study was conducted.

<u>www.clinicaltrials.gov</u>

Pfizer posts clinical trial results on www.clinicaltrials.gov for Pfizer-sponsored interventional studies (conducted in patients) that evaluate the safety and/or efficacy of a product,

090177e1968b9275\Approved\Approved On: 18-Mar-2021 07:58 (GMT)

regardless of the geographical location in which the study is conducted. These results are submitted for posting in accordance with the format and timelines set forth by US law.

EudraCT

Pfizer posts clinical trial results on EudraCT for Pfizer-sponsored interventional studies in accordance with the format and timelines set forth by EU requirements.

www.pfizer.com

Pfizer posts public disclosure synopses (CSR synopses in which any data that could be used to identify individual participants have been removed) on www.pfizer.com for Pfizer-sponsored interventional studies at the same time the corresponding study results are posted to www.clinicaltrials.gov.

Documents within marketing authorization packages/submissions

Pfizer complies with the European Union Policy 0070, the proactive publication of clinical data to the EMA website. Clinical data, under Phase 1 of this policy, includes clinical overviews, clinical summaries, CSRs, and appendices containing the protocol and protocol amendments, sample CRFs, and statistical methods. Clinical data, under Phase 2 of this policy, includes the publishing of individual participant data. Policy 0070 applies to new marketing authorization applications submitted via the centralized procedure since 01 January 2015 and applications for line extensions and for new indications submitted via the centralized procedure since 01 July 2015.

Data Sharing

Pfizer provides researchers secure access to patient-level data or full CSRs for the purposes of "bona-fide scientific research" that contributes to the scientific understanding of the disease, target, or compound class. Pfizer will make available data from these trials 24 months after study completion. Patient-level data will be anonymized in accordance with applicable privacy laws and regulations. CSRs will have personally identifiable information redacted.

Data requests are considered from qualified researchers with the appropriate competencies to perform the proposed analyses. Research teams must include a biostatistician. Data will not be provided to applicants with significant conflicts of interest, including individuals requesting access for commercial/competitive or legal purposes.

090177e1968b9275\Approved\Approved On: 18-Mar-2021 07:58 (GMT)

10.1.6. Data Quality Assurance

All participant data relating to the study will be recorded on printed or electronic CRF unless transmitted to the sponsor or designee electronically (eg, laboratory data). The investigator is responsible for verifying that data entries are accurate and correct by physically or electronically signing the CRF.

The investigator must maintain accurate documentation (source data) that supports the information entered in the CRF.

The investigator must ensure that the CRFs are securely stored at the study site in encrypted electronic and/or paper form and are password protected or secured in a locked room to prevent access by unauthorized third parties.

The investigator must permit study-related monitoring, audits, IRB/EC review, and regulatory agency inspections and provide direct access to source data documents. This verification may also occur after study completion. It is important that the investigator(s) and their relevant personnel are available during the monitoring visits and possible audits or inspections and that sufficient time is devoted to the process.

Monitoring details describing strategy (eg, risk-based initiatives in operations and quality such as risk management and mitigation strategies and analytical risk-based monitoring), methods, responsibilities, and requirements, including handling of noncompliance issues and monitoring techniques (central, remote, or on-site monitoring), are provided in the monitoring plan.

The sponsor or designee is responsible for the data management of this study, including quality checking of the data.

Study monitors will perform ongoing source data verification to confirm that data entered into the CRF by authorized site personnel are accurate, complete, and verifiable from source documents; that the safety and rights of participants are being protected; and that the study is being conducted in accordance with the currently approved protocol and any other study agreements, ICH GCP, and all applicable regulatory requirements.

Records and documents, including signed ICDs, pertaining to the conduct of this study must be retained by the investigator for 15 years after study completion unless local regulations or institutional policies require a longer retention period. No records may be destroyed during the retention period without the written approval of the sponsor. No records may be transferred to another location or party without written notification to the sponsor. The investigator must ensure that the records continue to be stored securely for as long as they are maintained.

When participant data are to be deleted, the investigator will ensure that all copies of such data are promptly and irrevocably deleted from all systems.

090177e1968b9275\Approved\Approved On: 18-Mar-2021 07:58 (GMT)

The investigator(s) will notify the sponsor or its agents immediately of any regulatory inspection notification in relation to the study. Furthermore, the investigator will cooperate with the sponsor or its agents to prepare the investigator site for the inspection and will allow the sponsor or its agent, whenever feasible, to be present during the inspection. The investigator site and investigator will promptly resolve any discrepancies that are identified between the study data and the participant's medical records. The investigator will promptly provide copies of the inspection findings to the sponsor or its agent. Before response submission to the regulatory authorities, the investigator will provide the sponsor or its agents with an opportunity to review and comment on responses to any such findings.

10.1.7. Source Documents

Source documents provide evidence for the existence of the participant and substantiate the integrity of the data collected. Source documents are filed at the investigator site.

Data reported on the CRF or entered in the eCRF that are from source documents must be consistent with the source documents or the discrepancies must be explained. The investigator may need to request previous medical records or transfer records, depending on the study. Also, current medical records must be available.

Definition of what constitutes source data can be found in the study monitoring plan.

Description of the use of computerized system is documented in the Data Management Plan.

10.1.8. Study and Site Start and Closure

The study start date is the date on which the clinical study will be open for recruitment of participants.

The first act of recruitment is the date of the first participant's first visit and will be the study start date.

The sponsor designee reserves the right to close the study site or terminate the study at any time for any reason at the sole discretion of the sponsor. Study sites will be closed upon study completion. A study site is considered closed when all required documents and study supplies have been collected and a study-site closure visit has been performed.

The investigator may initiate study-site closure at any time upon notification to the sponsor or designee if requested to do so by the responsible IRB/EC or if such termination is required to protect the health of study participants.

Reasons for the early closure of a study site by the sponsor may include but are not limited to:

- Failure of the investigator to comply with the protocol, the requirements of the IRB/EC or local health authorities, the sponsor's procedures, or GCP guidelines;

090177e1968b9275\Approved\Approved On: 18-Mar-2021 07:58 (GMT)

- Inadequate recruitment of participants by the investigator;
- Discontinuation of further study intervention development.

If the study is prematurely terminated or suspended, the sponsor shall promptly inform the investigators, the ECs/IRBs, the regulatory authorities, and any CRO(s) used in the study of the reason for termination or suspension, as specified by the applicable regulatory requirements. The investigator shall promptly inform the participant and should assure appropriate participant therapy and/or follow-up.

Study termination is also provided for in the clinical study agreement. If there is any conflict between the contract and this protocol, the contract will control as to termination rights.

10.1.9. Publication Policy

The results of this study may be published or presented at scientific meetings by the investigator after publication of the overall study results or 1 year after the end of the study (or study termination), whichever comes first.

The investigator agrees to refer to the primary publication in any subsequent publications such as secondary manuscripts, and submits all manuscripts or abstracts to the sponsor 30 days before submission. This allows the sponsor to protect proprietary information and to provide comments and the investigator will, on request, remove any previously undisclosed confidential information before disclosure, except for any study- or Pfizer intervention-related information necessary for the appropriate scientific presentation or understanding of the study results.

For all publications relating to the study, the investigator will comply with recognized ethical standards concerning publications and authorship, including those established by the International Committee of Medical Journal Editors.

The sponsor will comply with the requirements for publication of the overall study results covering all investigator sites. In accordance with standard editorial and ethical practice, the sponsor will support publication of multicenter studies only in their entirety and not as individual site data. In this case, a coordinating investigator will be designated by mutual agreement.

Authorship of publications for the overall study results will be determined by mutual agreement and in line with International Committee of Medical Journal Editors authorship requirements.

If publication is addressed in the clinical study agreement, the publication policy set out in this section will not apply.

090177e1968b9275\Approved\Approved On: 18-Mar-2021 07:58 (GMT)

10.1.10. Sponsor's Qualified Medical Personnel

The contact information for the sponsor's appropriately qualified medical personnel for the study is documented in the study contact list located in the [supporting study documentation.

To facilitate access to appropriately qualified medical personnel on study-related medical questions or problems, participants are provided with a contact card at the time of informed consent. The contact card contains, at a minimum, protocol and study intervention identifiers, participant numbers, contact information for the investigator site, and contact details for a contact center in the event that the investigator site staff cannot be reached to provide advice on a medical question or problem originating from another healthcare professional not involved in the participant's participation in the study. The contact number can also be used by investigator staff if they are seeking advice on medical questions or problems; however, it should be used only in the event that the established communication pathways between the investigator site and the study team are not available. It is therefore intended to augment, but not replace, the established communication pathways between the investigator site and the study team for advice on medical questions or problems that may arise during the study. The contact number is not intended for use by the participant directly, and if a participant calls that number, he or she will be directed back to the investigator site.

090177e1968b9275\Approved\Approved On: 18-Mar-2021 07:58 (GMT)

10.2. Appendix 2: Clinical Laboratory Tests

The following safety laboratory tests will be performed at times defined in the SoA section of this protocol. Additional laboratory results may be reported on these samples as a result of the method of analysis or the type of analyzer used by the clinical laboratory, or as derived from calculated values. These additional tests would not require additional collection of blood. Unscheduled clinical laboratory measurements may be obtained at any time during the study to assess any perceived safety issues.

Hematology	Chemistry	Other
Hemoglobin<br>Hematocrit<br>RBC count<br>MCV<br>MCH<br>MCHC<br>Platelet count<br>WBC count<br>Total neutrophils (Abs)<br>Eosinophils (Abs)<br>Monocytes (Abs)<br>Basophils (Abs)<br>Lymphocytes (Abs)	BUN<br>Creatinine<br>AST<br>ALT<br>Total bilirubin<br>Alkaline phosphatase	• Urine pregnancy test (β-hCG)

Investigators must document their review of each laboratory safety report.

Clinically significant abnormal laboratory findings should be recorded in the AE CRF in accordance with the following grading scale (Table 6).

Table 6. Laboratory Abnormality Grading Scale

Hematology	Mild (Grade 1)	Moderate (Grade 2)	Severe (Grade 3)	Potentially Life Threatening (Grade 4)
Hemoglobin (Female) - g/dL	11.0 – 12.0	9.5 – 10.9	8.0 – 9.4	<8.0
Hemoglobin (Male) - g/dL	12.5 – 13.5	10.5 – 12.4	8.5 – 10.4	<8.5
WBC increase - cells/mm^3	10,800 – 15,000	15,001 – 20,000	20,001 – 25, 000	>25,000
WBC decrease - cells/mm^3	2,500 – 3,500	1,500 – 2,499	1,000 – 1,499	<1,000
Lymphocytes decrease - cells/mm^3	750 – 1,000	500 – 749	250 – 499	<250
Neutrophils decrease - cells/mm^3	1,500 – 2,000	1,000 – 1,499	500 – 999	<500
Eosinophils - cells/mm^3	650 – 1500	1501 - 5000	>5000	Hypereosinophilic
Platelets decreased - cells/mm^3	125,000 – 140,000	100,000 – 124,000	25,000 – 99,000	<25,000

090177e1968b9275\Approved\Approved On: 18-Mar-2021 07:58 (GMT)

Table 6. Laboratory Abnormality Grading Scale

Chemistry	Mild (Grade 1)	Moderate (Grade 2)	Severe (Grade 3)	Potentially Life Threatening (Grade 4)
BUN - mg/dL	23 – 26	27 – 31	> 31	Requires dialysis
Creatinine – mg/dL	1.5 – 1.7	1.8 – 2.0	2.1 – 2.5	> 2.5 or requires dialysis
Alkaline phosphate – increase by factor	1.1 – 2.0 x ULN	2.1 – 3.0 x ULN	3.1 – 10 x ULN	>10 x ULN
Liver function tests – ALT, AST increase by factor	1.1 – 2.5 x ULN	2.6 – 5.0 x ULN	5.1 – 10 x ULN	>10 x ULN
Bilirubin – when accompanied by any increase in liver function test - increase by factor	1.1 – 1.25 x ULN	1.26 – 1.5 x ULN	1.51 – 1.75 x ULN	>1.75 x ULN
Bilirubin – when liver function test is normal - increase by factor	1.1 – 1.5 x ULN	1.6 – 2.0 x ULN	2.0 – 3.0 x ULN	>3.0 x ULN

Abbreviations: ALT = alanine aminotransferase; AST = aspartate aminotransferase; BUN = blood urea nitrogen; ULN = upper limit of normal; WBC = white blood cell.

090177e1968b9275\Approved\Approved On: 18-Mar-2021 07:58 (GMT)

10.3. Appendix 3: Adverse Events: Definitions and Procedures for Recording, Evaluating, Follow-up, and Reporting

10.3.1. Definition of AE

AE Definition
• An AE is any untoward medical occurrence in a patient or clinical study participant, temporally associated with the use of study intervention, whether or not considered related to the study intervention.<br>• NOTE: An AE can therefore be any unfavorable and unintended sign (including an abnormal laboratory finding), symptom, or disease (new or exacerbated) temporally associated with the use of study intervention.

Events Meeting the AE Definition
• Any abnormal laboratory test results (hematology, clinical chemistry, or urinalysis) or other safety assessments (eg, ECG, radiological scans, vital sign measurements), including those that worsen from baseline, considered clinically significant in the medical and scientific judgment of the investigator Any abnormal laboratory test results that meet any of the conditions below must be recorded as an AE:<br>• Is associated with accompanying symptoms.<br>• Requires additional diagnostic testing or medical/surgical intervention.<br>• Leads to a change in study dosing (outside of any protocol-specified dose adjustments) or discontinuation from the study, significant additional concomitant drug treatment, or other therapy.<br>• Exacerbation of a chronic or intermittent preexisting condition including either an increase in frequency and/or intensity of the condition.<br>• New conditions detected or diagnosed after study intervention administration even though it may have been present before the start of the study.<br>• Signs, symptoms, or the clinical sequelae of a suspected drug-drug interaction.<br>• Signs, symptoms, or the clinical sequelae of a suspected overdose of either study intervention or a concomitant medication. Overdose per se will not be reported as an AE/SAE unless it is an intentional overdose taken with possible suicidal/self-harming intent. Such overdoses should be reported regardless of sequelae.

090177e1968b9275\Approved\Approved On: 18-Mar-2021 07:58 (GMT)

**Events NOT Meeting the AE Definition**
• Any clinically significant abnormal laboratory findings or other abnormal safety assessments which are associated with the underlying disease, unless judged by the investigator to be more severe than expected for the participant's condition.<br>• The disease/disorder being studied or expected progression, signs, or symptoms of the disease/disorder being studied, unless more severe than expected for the participant's condition.<br>• Medical or surgical procedure (eg, endoscopy, appendectomy): the condition that leads to the procedure is the AE.<br>• Situations in which an untoward medical occurrence did not occur (social and/or convenience admission to a hospital).<br>• Anticipated day-to-day fluctuations of preexisting disease(s) or condition(s) present or detected at the start of the study that do not worsen.

10.3.2. Definition of SAE

If an event is not an AE per definition above, then it cannot be an SAE even if serious conditions are met (eg, hospitalization for signs/symptoms of the disease under study, death due to progression of disease).

**An SAE is defined as any untoward medical occurrence that, at any dose:**
**a. Results in death**
**b. Is life-threatening**<br>The term "life-threatening" in the definition of "serious" refers to an event in which the participant was at risk of death at the time of the event. It does not refer to an event that hypothetically might have caused death if it were more severe.
**c. Requires inpatient hospitalization or prolongation of existing hospitalization**<br>In general, hospitalization signifies that the participant has been detained (usually involving at least an overnight stay) at the hospital or emergency ward for observation and/or treatment that would not have been appropriate in the physician's office or outpatient setting. Complications that occur during hospitalization are AEs. If a complication prolongs hospitalization or fulfills any other serious criteria, the event is serious. When in doubt as to whether "hospitalization" occurred or was necessary, the AE should be considered serious.

090177e1968b9275\Approved\Approved On: 18-Mar-2021 07:58 (GMT)

An SAE is defined as any untoward medical occurrence that, at any dose:
Hospitalization for elective treatment of a preexisting condition that did not worsen from baseline is not considered an AE.
**d. Results in persistent disability/incapacity**<br>• The term disability means a substantial disruption of a person's ability to conduct normal life functions.<br>• This definition is not intended to include experiences of relatively minor medical significance such as uncomplicated headache, nausea, vomiting, diarrhea, influenza, and accidental trauma (eg, sprained ankle) which may interfere with or prevent everyday life functions but do not constitute a substantial disruption.
**e. Is a congenital anomaly/birth defect**
**f. Other situations:**<br>• Medical or scientific judgment should be exercised in deciding whether SAE reporting is appropriate in other situations such as important medical events that may not be immediately life-threatening or result in death or hospitalization but may jeopardize the participant or may require medical or surgical intervention to prevent one of the other outcomes listed in the above definition. These events should usually be considered serious.<br>• Examples of such events include invasive or malignant cancers, intensive treatment in an emergency room or at home for allergic bronchospasm, blood dyscrasias or convulsions that do not result in hospitalization, or development of drug dependency or drug abuse.<br>• Suspected transmission via a Pfizer product of an infectious agent, pathogenic or non-pathogenic, is considered serious. The event may be suspected from clinical symptoms or laboratory findings indicating an infection in a patient exposed to a Pfizer product. The terms "suspected transmission" and "transmission" are considered synonymous. These cases are considered unexpected and handled as serious expedited cases by pharmacovigilance personnel. Such cases are also considered for reporting as product defects, if appropriate.

090177e1968b9275\Approved\Approved On: 18-Mar-2021 07:58 (GMT)

10.3.3. Recording/Reporting and Follow-up of AEs and/or SAEs

SAE Reporting to Pfizer Safety via an Electronic Data Collection Tool

- The primary mechanism for reporting an SAE to Pfizer Safety will be the electronic data collection tool.
- If the electronic system is unavailable, then the site will use the paper SAE data collection tool (see next section) in order to report the event within 24 hours.
- The site will enter the SAE data into the electronic system as soon as the data become available.
- After the study is completed at a given site, the electronic data collection tool will be taken off-line to prevent the entry of new data or changes to existing data.
- If a site receives a report of a new SAE from a study participant or receives updated data on a previously reported SAE after the electronic data collection tool has been taken off-line, then the site can report this information on a paper SAE form (see next section) or to Pfizer Safety by telephone.

AE and SAE Recording/Reporting

The table below summarizes the requirements for recording adverse events on the CRF and for reporting serious adverse events on the Vaccine SAE Reporting Form to Pfizer Safety. These requirements are delineated for 3 types of events: (1) SAEs; (2) nonserious adverse events (AEs); and (3) exposure to the study intervention under study during pregnancy or breastfeeding, and occupational exposure.

It should be noted that the Vaccine SAE Reporting Form for reporting of SAE information is not the same as the AE page of the CRF. When the same data are collected, the forms must be completed in a consistent manner. AEs should be recorded using concise medical terminology and the same AE term should be used on both the CRF and the Vaccine SAE Reporting Form for reporting of SAE information.

Safety Event	Recorded on the CRF	Reported on the Vaccine SAE Reporting Form to Pfizer Safety Within 24 Hours of Awareness
SAE	All	All
Nonserious AE	All	None

090177e1968b9275\Approved\Approved On: 18-Mar-2021 07:58 (GMT)

AE and SAE Recording/Reporting

Exposure to the study intervention under study during pregnancy or breastfeeding, and occupational exposure	All AEs/SAEs associated with exposure during pregnancy or breastfeeding<br><br>Occupational exposure is not recorded.	All (and EDP supplemental form for EDP)<br>Note: Include all SAEs associated with exposure during pregnancy or breastfeeding. Include all AEs/SAEs associated with occupational exposure.

- When an AE/SAE occurs, it is the responsibility of the investigator to review all documentation (eg, hospital progress notes, laboratory reports, and diagnostic reports) related to the event.
- The investigator will then record all relevant AE/SAE information in the CRF.
- It is **not** acceptable for the investigator to send photocopies of the participant's medical records to Pfizer Safety in lieu of completion of the Vaccine SAE Reporting Form/AE/SAE CRF page.
- There may be instances when copies of medical records for certain cases are requested by Pfizer Safety. In this case, all participant identifiers, with the exception of the participant number, will be redacted on the copies of the medical records before submission to Pfizer Safety.
- The investigator will attempt to establish a diagnosis of the event based on signs, symptoms, and/or other clinical information. Whenever possible, the diagnosis (not the individual signs/symptoms) will be documented as the AE/SAE.

090177e1968b9275\Approved\Approved On: 18-Mar-2021 07:58 (GMT)

AE and SAE Recording/Reporting

Assessment of Intensity

The investigator will make an assessment of intensity for each AE and SAE reported during the study and assign it to 1 of the following categories:

GRADE	If required on the AE page of the CRF, the investigator will use the adjectives MILD, MODERATE, SEVERE, or LIFE-THREATENING to describe the maximum intensity of the AE. For purposes of consistency, these intensity grades are defined as follows:	
1	MILD	Does not interfere with participant's usual function.
2	MODERATE	Interferes to some extent with participant's usual function.
3	SEVERE	Interferes significantly with participant's usual function.
4	LIFE-THREATENING	Life-threatening consequences; urgent intervention indicated.

Assessment of Causality

- The investigator is obligated to assess the relationship between study intervention and each occurrence of each AE/SAE.
- A “reasonable possibility” of a relationship conveys that there are facts, evidence, and/or arguments to suggest a causal relationship, rather than a relationship cannot be ruled out.
- The investigator will use clinical judgment to determine the relationship.
- Alternative causes, such as underlying disease(s), concomitant therapy, and other risk factors, as well as the temporal relationship of the event to study intervention administration, will be considered and investigated.
- The investigator will also consult the IB and/or product information, for marketed products, in his/her assessment.

090177e1968b9275\Approved\Approved On: 18-Mar-2021 07:58 (GMT)

**Assessment of Causality**
• For each AE/SAE, the investigator **must** document in the medical notes that he/she has reviewed the AE/SAE and has provided an assessment of causality.<br>• There may be situations in which an SAE has occurred and the investigator has minimal information to include in the initial report to the sponsor. However, **it is very important that the investigator always make an assessment of causality for every event before the initial transmission of the SAE data to the sponsor.**<br>• The investigator may change his/her opinion of causality in light of follow-up information and send an SAE follow-up report with the updated causality assessment.<br>• The causality assessment is one of the criteria used when determining regulatory reporting requirements.<br>• If the investigator does not know whether or not the study intervention caused the event, then the event will be handled as "related to study intervention" for reporting purposes, as defined by the sponsor. In addition, if the investigator determines that an SAE is associated with study procedures, the investigator must record this causal relationship in the source documents and CRF, and report such an assessment in the dedicated section of the Vaccine SAE Reporting Form and in accordance with the SAE reporting requirements.

**Follow-up of AEs and SAEs**
• The investigator is obligated to perform or arrange for the conduct of supplemental measurements and/or evaluations as medically indicated or as requested by the sponsor to elucidate the nature and/or causality of the AE or SAE as fully as possible. This may include additional laboratory tests or investigations, histopathological examinations, or consultation with other healthcare providers.<br>• If a participant dies during participation in the study or during a recognized follow-up period, the investigator will provide Pfizer Safety with a copy of any postmortem findings including histopathology.<br>• New or updated information will be recorded in the originally completed CRF.<br>• The investigator will submit any updated SAE data to the sponsor within 24 hours of receipt of the information.

090177e1968b9275\Approved\Approved On: 18-Mar-2021 07:58 (GMT)

10.3.4. Reporting of SAEs

SAE Reporting to Pfizer Safety via Vaccine SAE Reporting Form
• Facsimile transmission of the Vaccine SAE Reporting Form is the preferred method to transmit this information to Pfizer Safety.<br>• In circumstances when the facsimile is not working, notification by telephone is acceptable with a copy of the Vaccine SAE Reporting Form sent by overnight mail or courier service.<br>• Initial notification via telephone does not replace the need for the investigator to complete and sign the Vaccine SAE Reporting Form pages within the designated reporting time frames.

090177e1968b9275\Approved\Approved On: 18-Mar-2021 07:58 (GMT)

10.4. Appendix 4: Contraceptive Guidance

10.4.1. Male Participant Reproductive Inclusion Criteria

Male participants are eligible to participate if they agree to the following requirements during the intervention period and for at least 28 days after the last dose of study intervention, which corresponds to the time needed to eliminate reproductive safety risk of the study intervention(s):

- Refrain from donating sperm.

PLUS either:

- Be abstinent from heterosexual intercourse with a female of childbearing potential as their preferred and usual lifestyle (abstinent on a long term and persistent basis) and agree to remain abstinent.

  OR

- Must agree to use a male condom when engaging in any activity that allows for passage of ejaculate to another person.

- In addition to male condom use, a highly effective method of contraception may be considered in WOCBP partners of male participants (refer to the list of highly effective methods below in Section 10.4.4).

10.4.2. Female Participant Reproductive Inclusion Criteria

A female participant is eligible to participate if she is not pregnant or breastfeeding, and at least 1 of the following conditions applies:

- Is not a WOCBP (see definitions below in Section 10.4.3).

  OR

- Is a WOCBP and using an acceptable contraceptive method as described below during the intervention period (for a minimum of 28 days after the last dose of study intervention). The investigator should evaluate the effectiveness of the contraceptive method in relationship to the first dose of study intervention.

The investigator is responsible for review of medical history, menstrual history, and recent sexual activity to decrease the risk for inclusion of a woman with an early undetected pregnancy.

090177e1968b9275\Approved\Approved On: 18-Mar-2021 07:58 (GMT)

10.4.3. Woman of Childbearing Potential

A woman is considered fertile following menarche and until becoming postmenopausal unless permanently sterile (see below).

If fertility is unclear (eg, amenorrhea in adolescents or athletes) and a menstrual cycle cannot be confirmed before the first dose of study intervention, additional evaluation should be considered.

Women in the following categories are not considered WOCBP:

1. Premenopausal female with 1 of the following:
   - Documented hysterectomy;
   - Documented bilateral salpingectomy;
   - Documented bilateral oophorectomy.

For individuals with permanent infertility due to an alternate medical cause other than the above, (eg, mullerian agenesis, androgen insensitivity), investigator discretion should be applied to determining study entry.

Note: Documentation for any of the above categories can come from the site personnel's review of the participant's medical records, medical examination, or medical history interview. The method of documentation should be recorded in the participant's medical record for the study.

2. Postmenopausal female:
   - A postmenopausal state is defined as no menses for 12 months without an alternative medical cause. In addition, a
     - high FSH level in the postmenopausal range must be used to confirm a postmenopausal state in women under 60 years of age and not using hormonal contraception or HRT.
     - Female on HRT and whose menopausal status is in doubt will be required to use one of the nonestrogen hormonal highly effective contraception methods if they wish to continue their HRT during the study. Otherwise, they must discontinue HRT to allow confirmation of postmenopausal status before study enrollment.

090177e1968b9275\Approved\Approved On: 18-Mar-2021 07:58 (GMT)

10.4.4. Contraception Methods

Contraceptive use by men or women should be consistent with local availability/regulations regarding the use of contraceptive methods for those participating in clinical trials.

1. Implantable progestogen-only hormone contraception associated with inhibition of ovulation.[a]

2. Intrauterine device.

3. Intrauterine hormone-releasing system.

4. Bilateral tubal occlusion.

5. Vasectomized partner:

   - Vasectomized partner is a highly effective contraceptive method provided that the partner is the sole sexual partner of the woman of childbearing potential and the absence of sperm has been confirmed. If not, an additional highly effective method of contraception should be used. The spermatogenesis cycle is approximately 90 days.

6. Combined (estrogen- and progestogen-containing) hormonal contraception associated with inhibition of ovulation:

   - Oral;
   - Intravaginal[a];
   - Transdermal[a];
   - Injectable.[a]

7. Progestogen-only hormone contraception associated with inhibition of ovulation:

   - Oral[a];
   - Injectable.[a]

[a] Unapproved in Japan.

090177e1968b9275\Approved\Approved On: 18-Mar-2021 07:58 (GMT)

8. Sexual abstinence:

   - Sexual abstinence is considered a highly effective method only if defined as refraining from heterosexual intercourse during the entire period of risk associated with the study intervention. The reliability of sexual abstinence needs to be evaluated in relation to the duration of the study and the preferred and usual lifestyle of the participant.

9. Progestogen-only oral hormonal contraception where inhibition of ovulation is not the primary mode of action.[a]

10. Male or female condom with or without spermicide.

11. Cervical cap,[a] diaphragm,[a] or sponge with spermicide.[a]

12. A combination of male condom with either cervical cap,[a] diaphragm,[a] or sponge with spermicide[a] (double-barrier methods).

[a] Unapproved in Japan.

090177e1968b9275\Approved\Approved On: 18-Mar-2021 07:58 (GMT)

10.5. Appendix 5: Genetics

Not applicable.

10.6. Appendix 6: Liver Safety: Suggested Actions and Follow-up Assessments

Potential Cases of Drug-Induced Liver Injury

Humans exposed to a drug who show no sign of liver injury (as determined by elevations in transaminases) are termed "tolerators," while those who show transient liver injury, but adapt are termed "adaptors." In some participants, transaminase elevations are a harbinger of a more serious potential outcome. These participants fail to adapt and therefore are "susceptible" to progressive and serious liver injury, commonly referred to as DILI. Participants who experience a transaminase elevation above 3 × ULN should be monitored more frequently to determine if they are an "adaptor" or are "susceptible."

LFTs are not required as a routine safety monitoring procedure in this study. However, should an investigator deem it necessary to assess LFTs because a participant presents with clinical signs/symptoms, such LFT results should be managed and followed as described below.

In the majority of DILI cases, elevations in AST and/or ALT precede TBili elevations (>2 × ULN) by several days or weeks. The increase in TBili typically occurs while AST/ALT is/are still elevated above 3 × ULN (ie, AST/ALT and TBili values will be elevated within the same laboratory sample). In rare instances, by the time TBili elevations are detected, AST/ALT values might have decreased. This occurrence is still regarded as a potential DILI. Therefore, abnormal elevations in either AST OR ALT in addition to TBili that meet the criteria outlined below are considered potential DILI (assessed per Hy's law criteria) cases and should always be considered important medical events, even before all other possible causes of liver injury have been excluded.

The threshold of laboratory abnormalities for a potential DILI case depends on the participant's individual baseline values and underlying conditions. Participants who present with the following laboratory abnormalities should be evaluated further as potential DILI (Hy's law) cases to definitively determine the etiology of the abnormal laboratory values:

- Participants with AST/ALT and TBili baseline values within the normal range who subsequently present with AST OR ALT values >3 × ULN AND a TBili value >2 × ULN with no evidence of hemolysis and an alkaline phosphatase value <2 × ULN or not available.

- For participants with baseline AST **OR** ALT **OR** TBili values above the ULN, the following threshold values are used in the definition mentioned above, as needed, depending on which values are above the ULN at baseline:

  - Preexisting AST or ALT baseline values above the normal range: AST or ALT values >2 times the baseline values AND >3 × ULN; or >8 × ULN (whichever is smaller).

090177e1968b9275\Approved\Approved On: 18-Mar-2021 07:58 (GMT)

- Preexisting values of TBili above the normal range: TBili level increased from baseline value by an amount of at least 1 × ULN or if the value reaches >3 × ULN (whichever is smaller).

Rises in AST/ALT and TBili separated by more than a few weeks should be assessed individually based on clinical judgment; any case where uncertainty remains as to whether it represents a potential Hy’s law case should be reviewed with the sponsor.

The participant should return to the investigator site and be evaluated as soon as possible, preferably within 48 hours from awareness of the abnormal results. This evaluation should include laboratory tests, detailed history, and physical assessment.

In addition to repeating measurements of AST and ALT and TBili for suspected cases of Hy’s law, additional laboratory tests should include albumin, CK, direct and indirect bilirubin, GGT, PT/INR, total bile acids, and alkaline phosphatase. Consideration should also be given to drawing a separate tube of clotted blood and an anticoagulated tube of blood for further testing, as needed, for further contemporaneous analyses at the time of the recognized initial abnormalities to determine etiology. A detailed history, including relevant information, such as review of ethanol, acetaminophen/paracetamol (either by itself or as a coformulated product in prescription or over-the-counter medications), recreational drug, supplement (herbal) use and consumption, family history, sexual history, travel history, history of contact with a jaundiced person, surgery, blood transfusion, history of liver or allergic disease, and potential occupational exposure to chemicals, should be collected. Further testing for acute hepatitis A, B, C, D, and E infection and liver imaging (eg, biliary tract) and collection of serum samples for acetaminophen/paracetamol drug and/or protein adduct levels may be warranted.

All cases demonstrated on repeat testing as meeting the laboratory criteria of AST/ALT and TBili elevation defined above should be considered potential DILI (Hy’s law) cases if no other reason for the LFT abnormalities has yet been found. **Such potential DILI (Hy’s law) cases are to be reported as SAEs, irrespective of availability of all the results of the investigations performed to determine etiology of the LFT abnormalities.**

A potential DILI (Hy’s law) case becomes a confirmed case only after all results of reasonable investigations have been received and have excluded an alternative etiology.

090177e1968b9275\Approved\Approved On: 18-Mar-2021 07:58 (GMT)

090177e1968b9275\Approved\Approved On: 18-Mar-2021 07:58 (GMT)

10.7. Appendix 7: Abbreviations

The following is a list of abbreviations that may be used in the protocol.

Abbreviation	Term
2019-nCoV	novel coronavirus 2019
Abs	absolute
AE	adverse event
AESI	adverse event of special interest
ALT	alanine aminotransferase
AST	aspartate aminotransferase
β-hCG	beta-human chorionic gonadotropin
BUN	blood urea nitrogen
CBER	Center for Biologics Evaluation and Research
CFR	Code of Federal Regulations
CI	confidence interval
CIOMS	Council for International Organizations of Medical Sciences
CK	creatine kinase
CONSORT	Consolidated Standards of Reporting Trials
COVID-19	coronavirus disease 2019
CRA	clinical research associate
CRF	case report form
CRO	contract research organization
CSR	clinical study report
DILI	drug-induced liver injury
DMC	data monitoring committee
DNA	deoxyribonucleic acid
EC	ethics committee
ECG	electrocardiogram
ECLIA	electrochemiluminescence immunoassay
eCRF	electronic case report form
e-diary	electronic diary
EDP	exposure during pregnancy
EMA	European Medicines Agency
EU	European Union
EudraCT	European Clinical Trials Database
FDA	Food and Drug Administration
FSH	follicle-stimulating hormone
GCP	Good Clinical Practice
GGT	gamma-glutamyl transferase
GM	geometric mean
GMFR	geometric mean fold rise
GMT	geometric mean titer
GPSP	Good Postmarketing Study Practice

Abbreviation	Term
HBV	hepatitis B virus
HCV	hepatitis C virus
HIPAA	Health Insurance Portability and Accountability Act
HIV	human immunodeficiency virus
HR	heart rate
HRT	hormone replacement therapy
IB	investigator’s brochure
ICD	informed consent document
ICH	International Council for Harmonisation
ID	identification
IgG	immunoglobulin G
IMP	investigational medicinal product
IND	investigational new drug
INR	international normalized ratio
IP manual	investigational product manual
IPAL	Investigational Product Accountability Log
IRB	institutional review board
IRT	interactive response technology
IV	intravenous(ly)
IWR	interactive Web-based response
LFT	liver function test
LLOQ	lower limit of quantitation
LNP	lipid nanoparticle
MCH	mean corpuscular hemoglobin
MCHC	mean corpuscular hemoglobin concentration
MCV	mean corpuscular volume
MedDRA	Medical Dictionary for Regulatory Activities
MERS	Middle East respiratory syndrome
MHLW	Ministry of Health, Labour and Welfare
modRNA	nucleoside-modified messenger ribonucleic acid
N	nucleoprotein
N/A	not applicable
NIMP	noninvestigational medicinal product
non-S	nonspike protein
P2 S	SARS-CoV-2 full-length, P2 mutant, prefusion spike glycoprotein
PI	principal investigator
PPE	personal protective equipment
PT	prothrombin time
RBC	red blood cell
RBD	receptor-binding domain
RCDC	reverse cumulative distribution curve
RNA	ribonucleic acid

090177e1968b9275\Approved\Approved On: 18-Mar-2021 07:58 (GMT)

Abbreviation	Term
RSV	respiratory syncytial virus
S1	spike protein S1 subunit
SAE	serious adverse event
SAP	statistical analysis plan
SARS	severe acute respiratory syndrome
SARS-CoV-2	severe acute respiratory syndrome coronavirus 2
SoA	schedule of activities
SOP	standard operating procedure
SRSD	single reference safety document
SUSAR	suspected unexpected serious adverse reaction
TBili	total bilirubin
ULN	upper limit of normal
uRNA	unmodified messenger ribonucleic acid
US	United States
vax	vaccination
WBC	white blood cell
WHO	World Health Organization
WOCBP	woman/women of childbearing potential

090177e1968b9275\Approved\Approved On: 18-Mar-2021 07:58 (GMT)

11. REFERENCES

[1] World Health Organization. WHO Director-General's opening remarks at the media briefing on COVID-19. Available from: https://www.who.int/dg/speeches/detail/who-director-general-s-opening-remarks-at-the-media-briefing-on-covid-19---11-march-2020. Published: 11 Mar 2020. Accessed: 01 Apr 2020.

[2] World Health Organization. Coronavirus disease 2019 (COVID-19) situation report - 70. In: Data as reported by national authorities by 10:00 CET 30 March 2020. Geneva, Switzerland: World Health Organization; 2020.

[3] Centers for Disease Control and Prevention. Coronavirus disease 2019 (COVID-19): information for clinicians on investigational therapeutics for patients with COVID-19. Available from: https://www.cdc.gov/coronavirus/2019-ncov/hcp/therapeutic-options.html. Updated: 25 Apr 2020. Accessed: 26 Jun 2020.

[4] Rauch S, Jasny E, Schmidt KE, et al. New vaccine technologies to combat outbreak situations. Front Immunol 2018;9:1963.

[5] Sahin U, Karikó K, Türeci Ö. mRNA-based therapeutics—developing a new class of drugs. Nat Rev Drug Discov 2014;13(10):759-80.

[6] BioNTech RNA Pharmaceuticals GmbH. CorVAC/BNT162 Investigator’s Brochure. Mainz, Germany: BioNTech RNA Pharmaceuticals GmbH; 25 Mar 2020.

[7] Feldman RA, Fuhr R, Smolenov I, et al. mRNA vaccines against H10N8 and H7N9 influenza viruses of pandemic potential are immunogenic and well tolerated in healthy adults in phase 1 randomized clinical trials. Vaccine 2019;37(25):3326-34.

[8] US Food and Drug Administration. Guidance for industry: toxicity grading scale for healthy adult and adolescent volunteers enrolled in preventive vaccine clinical trials. Rockville, MD: Center for Biologics Evaluation and Research; September 2007.

[9] Miettinen O, Nurminen M. Comparative analysis of two rates. Stat Med 1985;4(2):213-26.

090177e1968b9275\Approved\Approved On: 18-Mar-2021 07:58 (GMT)

Document Approval Record

**Document Name:**	C4591005 Protocol Amendment 3, Clean Copy, 17Mar2021
**Document Title:**	A PHASE 1/2, PLACEBO-CONTROLLED, RANDOMIZED, AND OBSERVER-BLIND STUDY TO EVALUATE THE SAFETY, TOLERABILITY, AND IMMUNOGENICITY OF A SARS-COV-2 RNA VACCINE CANDIDATE AGAINST COVID-19 IN HEALTHY JAPANESE ADULTS

Signed By:	Date(GMT)	Signing Capacity
[redacted]	18-Mar-2021 02:33:20	Final Approval
[redacted]	18-Mar-2021 07:58:52	Business Line Approver

090177e1968b9275\Approved\Approved On: 18-Mar-2021 07:58 (GMT)
