# Supplementary material for: A randomized study to evaluate safety and immunogenicity of the BNT162b2 COVID-19 vaccine in healthy Japanese adults
Source: Nat Commun. 2021 Dec 14;12:7105. doi: 10.1038/s41467-021-27316-2 (PMC8671474; doi:10.1038/s41467-021-27316-2)
Supplement: Supplementary file 2 — Reporting Summary [file 41467_2021_27316_MOESM2_ESM.pdf]

nature portfolio

Corresponding author(s): James Baber

Last updated by author(s): Sep 28, 2021

Reporting Summary

Nature Portfolio wishes to improve the reproducibility of the work that we publish. This form provides structure for consistency and transparency in reporting. For further information on Nature Portfolio policies, see our Editorial Policies and the Editorial Policy Checklist.

Statistics

For all statistical analyses, confirm that the following items are present in the figure legend, table legend, main text, or Methods section.

n/a	Confirmed	
☐	☒	The exact sample size (*n*) for each experimental group/condition, given as a discrete number and unit of measurement
☐	☒	A statement on whether measurements were taken from distinct samples or whether the same sample was measured repeatedly
☒	☐	The statistical test(s) used AND whether they are one- or two-sided<br>*Only common tests should be described solely by name; describe more complex techniques in the Methods section.*
☒	☐	A description of all covariates tested
☒	☐	A description of any assumptions or corrections, such as tests of normality and adjustment for multiple comparisons
☐	☒	A full description of the statistical parameters including central tendency (e.g. means) or other basic estimates (e.g. regression coefficient) AND variation (e.g. standard deviation) or associated estimates of uncertainty (e.g. confidence intervals)
☒	☐	For null hypothesis testing, the test statistic (e.g. *F*, *t*, *r*) with confidence intervals, effect sizes, degrees of freedom and *P* value noted<br>*Give P values as exact values whenever suitable.*
☒	☐	For Bayesian analysis, information on the choice of priors and Markov chain Monte Carlo settings
☒	☐	For hierarchical and complex designs, identification of the appropriate level for tests and full reporting of outcomes
☒	☐	Estimates of effect sizes (e.g. Cohen's *d*, Pearson's *r*), indicating how they were calculated

Our web collection on statistics for biologists contains articles on many of the points above.

Software and code

Policy information about availability of computer code

Data collection	e-diary: Trial manager version 6.0, CRF: InForm Ver6.3,
Data analysis	SAS Ver9.4

For manuscripts utilizing custom algorithms or software that are central to the research but not yet described in published literature, software must be made available to editors and reviewers. We strongly encourage code deposition in a community repository (e.g. GitHub). See the Nature Portfolio guidelines for submitting code & software for further information.

Data

Policy information about availability of data

All manuscripts must include a data availability statement. This statement should provide the following information, where applicable:

- Accession codes, unique identifiers, or web links for publicly available datasets
- A description of any restrictions on data availability
- For clinical datasets or third party data, please ensure that the statement adheres to our policy

Upon request, and subject to certain criteria, conditions and exceptions (see https://www.pfizer.com/science/clinical-trials/trial-data-and-results for more information), Pfizer will provide access to individual de-identified participant data from Pfizer-sponsored global interventional clinical studies conducted for medicines, vaccines and medical devices (1) for indications that have been approved in the US and/or EU or (2) in programs that have been terminated (i.e., development for all indications has been discontinued). Pfizer will also consider requests for the protocol, data dictionary, and statistical analysis plan. Data may be requested from Pfizer trials 24 months after study completion. The de-identified participant data will be made available to researchers whose proposals meet the research criteria and other conditions, and for which an exception does not apply, via a secure portal. To gain access, data requestors must enter into a data access agreement with Pfizer.

Field-specific reporting

Please select the one below that is the best fit for your research. If you are not sure, read the appropriate sections before making your selection.

- [x] Life sciences
- [ ] Behavioural & social sciences
- [ ] Ecological, evolutionary & environmental sciences

For a reference copy of the document with all sections, see nature.com/documents/nr-reporting-summary-flat.pdf

Life sciences study design

All studies must disclose on these points even when the disclosure is negative.

Sample size	The study sample size is not based on any statistical hypothesis testing as end points were descriptive
Data exclusions	All available safety and immunogenicity data through the primary analysis time point were included; additional data will be available after study completion
Replication	There was no attempt at replication of study findings
Randomization	Participants were randomized (3:1) to receive 2 doses, 21 days apart, of 30 µg BNT162b2 or placebo using an interactive web-based response system
Blinding	In this observer-blinded study, the study staff receiving, storing, dispensing, preparing, and administering the study interventions were unblinded. All other study and site personnel, including the investigator, investigator staff, and participants, were blinded to study intervention. To facilitate rapid review of data in real time, the majority of sponsor staff were unblinded to study intervention allocation for all participants. All laboratory testing personnel performing serology assays were blinded to study intervention assigned/received throughout the study.

Reporting for specific materials, systems and methods

We require information from authors about some types of materials, experimental systems and methods used in many studies. Here, indicate whether each material, system or method listed is relevant to your study. If you are not sure if a list item applies to your research, read the appropriate section before selecting a response.

Materials & experimental systems

n/a	Involved in the study	
x		Antibodies
	x	Eukaryotic cell lines
x		Palaeontology and archaeology
x		Animals and other organisms
	x	Human research participants
	x	Clinical data
x		Dual use research of concern

Methods

n/a	Involved in the study	
x		ChIP-seq
x		Flow cytometry
x		MRI-based neuroimaging

Eukaryotic cell lines

Policy information about cell lines

Cell line source(s)	Vero cells (for assay, ATCC) Vero E6 for virus propagation
Authentication	Vero cells were authenicated for identity, purity and genetic stability by BioReliance. Vero E6 have not been characterized.
Mycoplasma contamination	Each master bank vial is tested for mycoplasma contamination
Commonly misidentified lines (See ICLAC register)	None

Human research participants

Policy information about studies involving human research participants

Population characteristics	160 individuals were randomized at 2 sites in Japan to receive 2 doses of 30 µg BNT162b2 (n=119) or placebo (n=41) 21 days apart. All participants were Japanese, 51% were male, and the mean age was 46 years (range 20–76 years). The most commonly reported comorbidities across all participants were dyslipidemia (4/119 [3.4%] BNT162b2 recipients; 2/41 [4.9%] placebo recipients) and hypertension (2/119 [1.7%] BNT162b2 recipients; 2/41 [4.9%] placebo recipients).
Recruitment	Participants were recruited from healthy volunteer databases. Adults 20-85 years of age, including those with stable preexisting disease were enrolled. Participants with known infection with hepatitis B virus or hepatitis C virus, or HIV, a history of severe allergic reactions associated with vaccination, with previous confirmed COVID-19, with diagnosis of an immunocompromising or immunodeficiency disorder, and those who were pregnant or breastfeeding were excluded. Receipt of medicines intended to prevent COVID-19, previous vaccination with any coronavirus vaccine, and treatment with immunosuppressive therapy were also exclusion criteria.
Ethics oversight	This study was conducted in accordance with the study protocol and principles derived from international guidelines including the International Council for Harmonisation Guidelines for Good Clinical Practice, the Declaration of Helsinki, and applicable laws and regulations including privacy laws. The study protocol, informed consent documents, and other relevant documents were prospectively approved by institutional review board/ethics committees at each study site. Written informed consent was obtained from all participants before enrollment and before participation in any study-related procedures.

Note that full information on the approval of the study protocol must also be provided in the manuscript.

Clinical data

Policy information about clinical studies

All manuscripts should comply with the ICMJEguidelines for publication of clinical research and a completedCONSORT checklist must be included with all submissions.

Clinical trial registration	NCT04588480
Study protocol	The protocol has been submitted
Data collection	Data were collected in 1 hospital and 1 clinic. To evaluate vaccine-associated acute reactions, participants were observed at the study sites for 30 minutes after each vaccination. Local reactions or systemic events for 7 days after each dose were collected by electronic diary. Adverse events were collected from 1 month through 1 month after dose 2 (12 months after dose 2 for serious adverse events). Hematology and clinical chemistry laboratory parameters up to 7 days after dose 2 were assessed in the first 24 participants. Immunogenicity assessments on sera were conducted before doses 1 and 2, and up to 1 month after dose 2.
Outcomes	The primary safety objective was to describe the safety and tolerability of 2 doses of BNT162b2. Safety endpoints included assessment of reactogenicity, adverse events, serious adverse events, and hematology and clinical chemistry laboratory parameters. The primary immunogenicity objective was to describe the immune responses elicited by BNT162b2. Primary immunogenicity endpoints were geometric mean titers of SARS-CoV-2 neutralization 1 month after dose 2, and geometric mean fold rises of neutralizing titers from baseline to 1 month after dose 2. Secondary immunogenicity endpoints were geometric mean titers at time points up to 12 months after dose 2 (the study is ongoing).
